# Glycopolymer Inhibitors of Galectin-3 Suppress the Markers of Tissue Remodeling in Pulmonary Hypertension

**DOI:** 10.1021/acs.jmedchem.4c00341

**Published:** 2024-06-03

**Authors:** Antonín Sedlář, David Vrbata, Kateřina Pokorná, Kristýna Holzerová, Jakub Červený, Olga Kočková, Markéta Hlaváčková, Martina Doubková, Jana Musílková, Vladimír Křen, František Kolář, Lucie Bačáková, Pavla Bojarová

**Affiliations:** † Laboratory of Biomaterials and Tissue Engineering, Institute of Physiology of the Czech Academy of Sciences, Vídeňská 1083, Prague 4 CZ-142 00, Czech Republic; ‡ Laboratory of Biotransformation, Institute of Microbiology of the Czech Academy of Sciences, Vídeňská 1083, Prague 4 CZ-142 00, Czech Republic; § Laboratory of Developmental Cardiology, Institute of Physiology of the Czech Academy of Sciences, Vídeňská 1083, Prague 4 CZ-142 00, Czech Republic; ∥ Department of Analytical Chemistry, Faculty of Science, Charles University, Hlavova 8, Prague 2 CZ-128 43, Czech Republic; ⊥ Laboratory of Analytical Chemistry, Institute of Macromolecular Chemistry of the Czech Academy of Sciences, Heyrovského nám. 1888, Prague 6 CZ-162 00, Czech Republic; # Department of Health Care Disciplines and Population Protection, Faculty of Biomedical Engineering, Czech Technical University in Prague, nám. Sítná 3105, Kladno CZ-272 01, Czech Republic

## Abstract

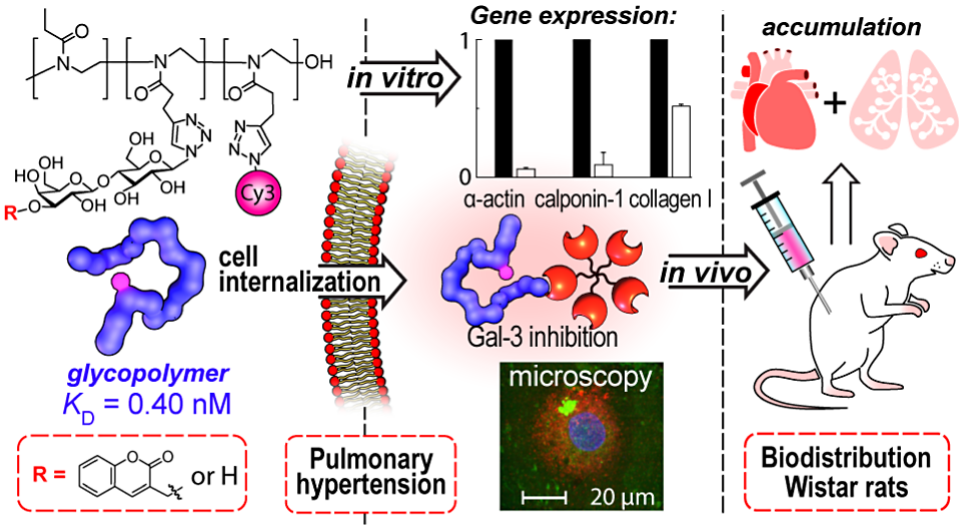

Pulmonary hypertension is a cardiovascular disease with a low survival rate. The protein galectin-3 (Gal-3) binding β-galactosides of cellular glycoproteins plays an important role in the onset and development of this disease. Carbohydrate-based drugs that target Gal-3 represent a new therapeutic strategy in the treatment of pulmonary hypertension. Here, we present the synthesis of novel hydrophilic glycopolymer inhibitors of Gal-3 based on a polyoxazoline chain decorated with carbohydrate ligands. Biolayer interferometry revealed a high binding affinity of these glycopolymers to Gal-3 in the subnanomolar range. In the cell cultures of cardiac fibroblasts and pulmonary artery smooth muscle cells, the most potent glycopolymer **18** (Lac-high) caused a decrease in the expression of markers of tissue remodeling in pulmonary hypertension. The glycopolymers were shown to penetrate into the cells. In a biodistribution and pharmacokinetics study in rats, the glycopolymers accumulated in heart and lung tissues, which are most affected by pulmonary hypertension.

## Introduction

Galectin-3 (Gal-3) is a β-galactosyl-binding lectin that specifically interacts with carbohydrate ligands presented, *e.g*., on the cell surface.^1^ This pleiotropic molecule has a wide range of functions *in vivo*, ranging from wound healing,^2^ osteogenesis, and chondrogenesis^3^ to endothelial tube formation.^4^ However, it is also implicated in the onset and progression of cancer^5^ and cardiovascular diseases, namely heart failure, atherosclerosis, and systemic and pulmonary hypertension.^6−8^

Pulmonary hypertension is a life-threatening disease, which affects pulmonary vessels and the heart. Its pathophysiology is marked by a progressive increase in pulmonary vascular resistance and remodeling, leading to right ventricular hypertrophy and, eventually, to heart failure. Pulmonary hypertension affects *ca.* 1% of the global population. In those aged over 65, its prevalence is estimated to be around 10%.^9^ Despite certain therapeutic advances, the long-term prognosis of patients with pulmonary hypertension is still poor.

There is evidence that Gal-3 is closely associated with vascular and myocardial remodeling and pathogenesis of heart failure of various origins. It has been shown that Gal-3 is a strong and independent prognostic biomarker of pulmonary hypertension regardless of etiology.^10^ Gal-3 has been reported to act as an important profibrotic agent that promotes the proliferation of α-smooth muscle actin (αSMA)-positive cells. These cells include vascular smooth muscle cells (VSMCs) located in the tunica media of the blood vessel wall,^6^ fibroblasts in the tunica adventitia,^11^ cardiac fibroblasts,^12^ and endothelial cells after their mesenchymal transition.^13^ Both VSMCs and fibroblasts can produce Gal-3, and its expression at mRNA and protein levels is increased in blood vessels during hypoxic pulmonary hypertension both *in vitro*(6,14) and *in vivo*.^6,13^

Several studies have shown that pharmacological inhibition or genetic disruption of Gal-3 has beneficial effects on cardiovascular diseases including hypoxic pulmonary hypertension.^15^ Similarly, the inhibition of Gal-3 expression by siRNA in chronically hypoxic mice reduced pulmonary hypertension, attenuated hypoxia-induced proliferation of pulmonary VSMCs, and suppressed their switching from a contractile to a synthetic phenotype.^14^ Gal-3 inhibition with disaccharide *N*-acetyllactosamine ameliorated hypoxic pulmonary hypertension and pulmonary vascular remodeling in rats.^11^

Gal-3 can be efficiently inhibited or scavenged by synthetic carbohydrates and glycomimetics. Its simplest ligands are disaccharides such as lactose (Galβ1,4Glc), LacNAc (*N*-acetyllactosamine; Galβ1,4GlcNAc), LacdiNAc (GalNAcβ1,4GlcNAc), or thiodigalactoside (Galβ1,1-S-Gal), which can be attached to various biocompatible carriers^16−18^ or chemically modified affording glycomimetics.^19^ In our earlier studies, poly-LacNAc-based oligosaccharide ligands of Gal-3 were bound to a scaffold of bovine serum albumin^5^ or attached to hydrophilic *N*-(2-hydroxypropyl)methacrylamide (HPMA) copolymer.^20,21^ The latter copolymer scaffold was also used for the multivalent presentation of thiodigalactoside-based glycomimetics.^17^ Our previous work with both nature-like oligosaccharides^16,18,20−22^ and glycomimetics^17,23^ repeatedly proved that by means of multivalent presentation, the resulting glycoconjugate affinity to galectins can be increased by several orders of magnitude, reaching low nanomolar or even picomolar levels. Thereby, each multivalent scaffold had its specific architectural features, and for maximizing the resulting affinity, it is vital to balance the ligand-scaffold synergy in view of hydrophobicity, sterical properties, flexibility, *etc.* Generally, as a rule of thumb, the molar content of an active oligosaccharide ligand may reach up to *ca.* 20 mol % in a multivalent system^21^ whereas with hydrophobic glycomimetics, a molar content higher than *ca.* 8 mol % usually leads to deteriorated properties and also lower affinity of the resulting glycoconjugate.^17,23^ Generally, synthetic polymers serve as versatile and tunable scaffolds for the multivalent presentation of carbohydrates. Their synthesis via living polymerizations affords highly defined materials tailored for specific applications. Among nonionic hydrophilic polymers such as poly(ethylene oxide) (PEO) and HPMA, polyoxazoline (POx) polymers have recently emerged as an attractive option due to their chemical versatility and low *in vivo* toxicity.^24^ In contrast to their HPMA and PEO counterparts, POx polymers are metabolized and excreted through the renal system without a significant accumulation in other organs.^25^ POx polymers are also thermoresponsive (except for polymethyloxazoline), *i.e*., they act as hydrophobes at a certain temperature termed lower critical solution temperature (LCST). By altering the monomer composition and derivatization of the POx backbone, the LCST can be tuned, which is often exploited in biomedical applications.^26^ Recently, POx polymers have been utilized as modulators of protein and drug delivery^27^ or hydrogels for tissue engineering applications.^28^ However, to our knowledge, POx polymers have never been used in combination with tailored carbohydrate ligands targeting a specific biomedical application. Advantageously, they can be deposited in specific tissues in the body. They can accumulate significantly in the lungs and heart, *i.e*. the tissues most affected by pulmonary hypertension.^29,30^

The present work encompasses the synthesis and characterization of novel glycopolymers based on the POx backbone bearing natural disaccharides or glycomimetics. The glycopolymers reversed Gal-3-induced changes in rat cardiac fibroblasts and VSMCs *in vitro* and accumulated in rat lung and heart tissue, highlighting their potential use for pulmonary hypertension therapy.

## Results and Discussion

### Synthesis and Characterization of Gal-3 Inhibitors

**Scheme 1:**
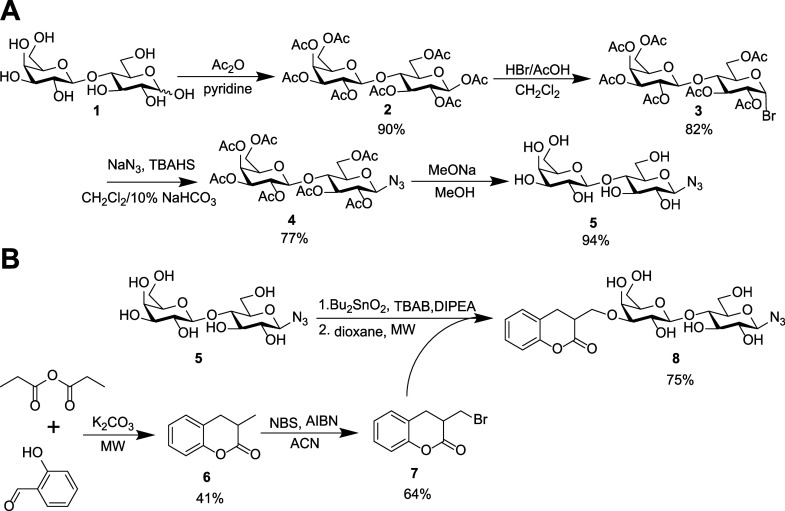
(A) Synthesis of Lactosyl Azide **5** and (B) Coumarylmethyllactosyl Glycomimetic **8**, Including Isolated Yields of Prepared Compounds

To demonstrate the effect of Gal-3 inhibition on the biological processes accompanying pulmonary hypertension, we prepared novel Gal-3 inhibitors based on carbohydrate-loaded POx polymers. Their low toxicity, immunogenicity, and chemical versatility make POx suitable carriers for carbohydrate-based drugs.^24^ Lactosyl (**5**; Scheme 1) and a specific glycomimetic based on 3-*O*-coumarylmethyllactosyl (**8**) azides were selected as carbohydrate ligands for multivalent presentation. Besides a higher affinity to Gal-3,^31−33^ the C-3 substitution on the galactosyl ring should ensure a prolonged half-life *in vivo*, though redeemed by a limited water solubility. Monovalent ligands **5** and **8** were prepared in good yields by a modification of the previously published procedures.^33^ Characterization of these ligands by HPLC and HRMS is shown in Figures S1–S4.

**Scheme 2:**
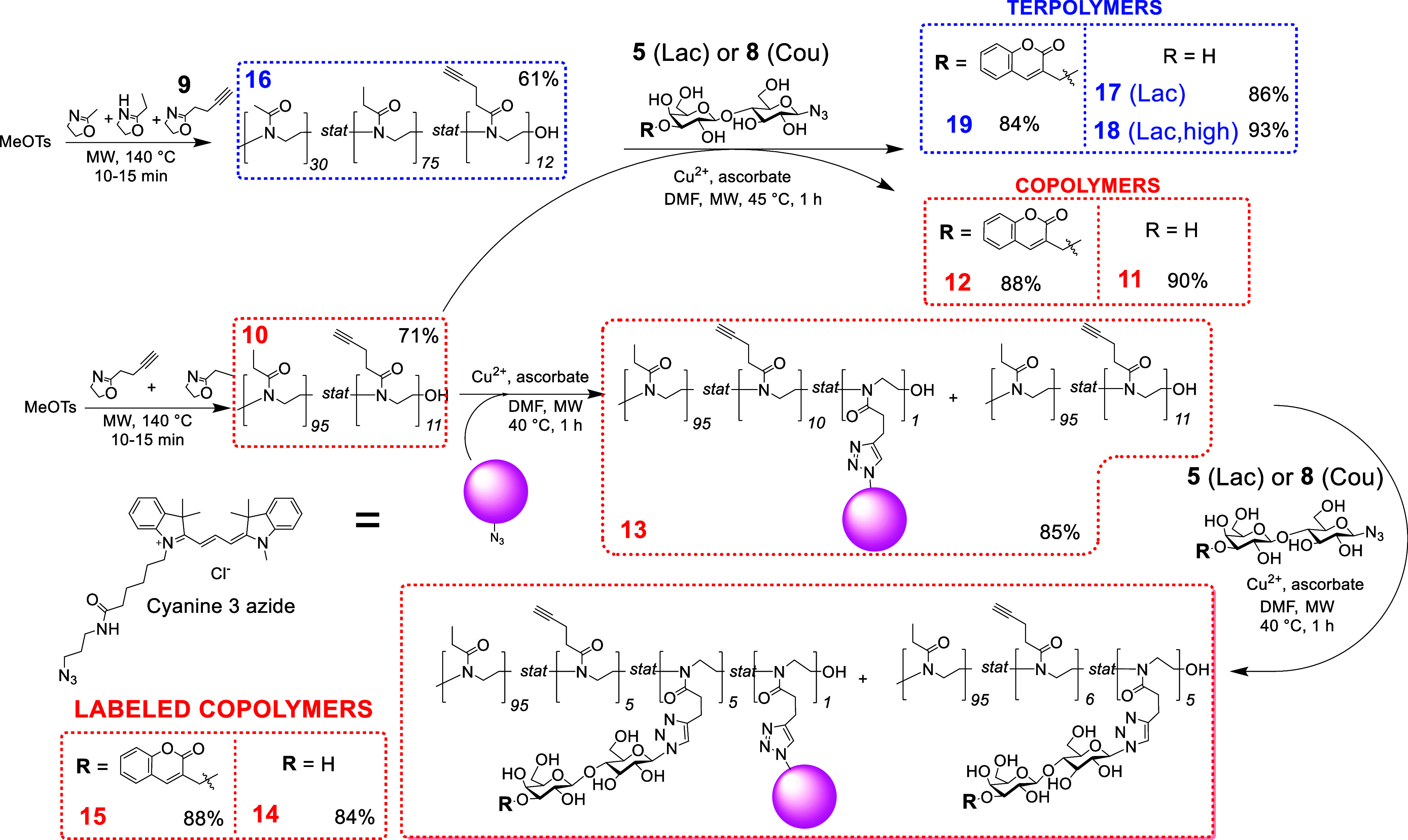
Overall Procedure for the Synthesis of POx-Based Glycopolymers Decorated with Lactose (**5**; Lac) or 3-*O*-coumarylmethyllactosyl (**8**; Cou). Isolated Yields of Glycopolymers are Given in Each Box

Monomer 2-(but-3-yn-1-yl)-4,5-dihydrooxazole (**9**) was synthesized according to a published procedure^34^ with some modifications, in an overall isolated yield of 32%. The HPLC and HRMS characterizations are shown in Figures S5 and S6. The statistical cationic ring-opening copolymerization of 2-ethyl-2-oxazoline and **9** was initiated by methyl *p*-toluensulfonate and proceeded in acetonitrile at 140 °C under microwave irradiation (Scheme 2). The molar ratio of the comonomer feed was *ca.* 0.87 EtOx and 0.13 BuOx; the resulting polymer had only a slightly different molar ratio (EtOx/BuOx, 0.89:0.11) as calculated by the end-group integration from the ^1^H NMR spectrum. The target molecular weight of the copolymer was 1.30 × 10^4^ g·mol^–1^ at 100% conversion; however, the polymerization was intentionally stopped at a lower conversion (71%, determined according to the isolated yield) to avoid chain transfer upon monomer depletion. The resulting POx copolymer precursor **10** had *M*_n_ = 10 630 g·mol^–1^, *M*_w_ = 13 030 g·mol^–1^, and dispersity *Đ* = 1.23 as determined by SEC-MALLS. The slightly enhanced dispersity may have been caused by chain termination (especially by water) at later stages of polymerization, which was detectable as a small low-molecular-weight shoulder on the SEC-MALLS chromatogram (Figure S8). To address the issue of POx thermoresponsiveness by modulating LCST, we also synthesized analogous terpolymers (Scheme 2) with the addition of MeOx monomer. The molar ratio of the comonomer feed for terpolymer **16** was MeOx/EtOx/BuOx, 33:55:11, and the target molecular weight at full conversion was 1.30 × 10^4^ g·mol^–1^. The polymerization was terminated at 61% conversion, and the final ratio of monomeric units determined by ^1^H NMR was MeOx/EtOx/BuOx, 25:65:10. The molecular properties and characteristics of the two prepared POx carriers are listed in Table 1 and Figures S7, S8, S19, and S20. The postpolymerization modification of cores **10** and **16** with carbohydrate ligands lactosyl **5** and 3-*O*-coumarylmethyllactosyl **8** azides (Table S1) produced respective copolymers **11** (Lac) and **12** (Cou), and terpolymers **17** (Lac), **18** (Lac-high), and **19** (Cou) in high yields. The conversions of click reactions were confirmed by ^1^H NMR; the molecular weight characteristics and dispersities of the resulting glycopolymers were evaluated by SEC-MALLS (Table 1). The reactions proceeded without significant precursor degradation as apparent from their properties given in Table 1 and Figures S9–S12 and S21–S26. The basic carbohydrate ligand content of 4–5 mol % was derived from previous thorough studies on the interaction of Gal-3 with various HPMA glycopolymers.^17^

**Table 1:** Physico-Chemical Parameters of POx-Based Glycopolymers Prepared in this Work

sample	*M*_n_^a^ [g·mol^–1^] × 10^3^	*M*_n_^b^ [g·mol^–1^] × 10^3^	*M*_w_^b^ [g·mol^–1^] × 10^3^	*Đ*^b^	ligand^c^	DS^d^ [mol %]
**10** (copolymer precursor)	10.8	10.6	13.0	1.23	-	-
**11** (Lac)	12.2	12.1	14.9	1.23	**5**	3.9
**12** (Cou)	13.4	11.5	14.9	1.29	**8**	4.0
**13** (Cy3-labeled precursor)^e^	10.9	n.a.	n.a.	1.10	-	-
**14** (Lac-Cy3)^e^	12.4	n.a.	n.a.	1.13	**5**	4.2
**15** (Cou-Cy3)^e^	13.5	n.a.	n.a.	1.10	**8**	4.8
**16** (terpolymer precursor)	11.3	8.7	10.8	1.25	-	-
**17** (Lac)	12.6	12.0	14.4	1.20	**5**	3.5
**18** (Lac-high)	14.4	16.0	19.1	1.19	**5**	8.4
**19** (Cou)	13.4	12.6	13.4	1.25	**8**	4.5

a Number-average molecular weights (*M*_n_) were obtained by end-group calculation from ^1^H NMR.

b Molecular weight characteristics (*M*_n_, *M*_w_, *Đ*) were obtained from SEC-MALLS measurements using refractive index increment d*n*/d*c* calculated from 100% mass recovery of the injected polymer sample.

c Azido-functionalized ligand coupled to the co/terpolymer precursor.

d DS, degree of substitution, indicating the molar content of ligand.

e Polymers labeled with cyanine 3 (Cy3) azide fluorescence marker (0.2 mol %).

The glycopolymers **12** (Cou) and **19** (Cou) decorated with the poorly soluble glycomimetic **8** were evaluated for the LCST parameter. In both cases, a significant drop in LCST compared to the respective parent polymer cores was observed: 17 °C vs 74 °C for **12** (Cou) and core **10**, respectively, and 8 °C vs >100 °C for **19** (Cou) and core **16**,^35^ respectively. We expected that terpolymer **19** (Cou) containing the “hydrophilic” MeOx monomeric units would have a significantly higher LCST after coupling with **8** compared to the copolymer. However, as can be seen from Table 1, the exact amount of attached **8** apparently influences LCST in **19** rather than the monomeric unit composition. The LCST is an important factor in further biological measurements as the polymers with a lower LCST may tend to aggregate in aqueous solutions; moreover, it may also ensure a higher bioavailability. To shed further light on this issue, the aggregation behavior of glycopolymers **12** (Cou) and **19** (Cou) was assessed by DLS in varying concentrations, times, and environments (water, DMEM culture medium and HBSS solution). The aggregation profiles of **12** (Cou) and **19** (Cou) were different. For glyco-copolymer **12** at *t* = 0 h, the intensity-weighted distribution showed a shift to lower particle sizes, *z*-averages, and lower PDI with decreasing concentration (Figure S29, S30), probably due to its slower aggregation. After 24 h, the solution was in pseudoequilibrium, containing particles of several hundred nanometers formed by further aggregation. In contrast, glyco-terpolymer **19** formed quite stable aggregates in sub-100 nm range with similar intensity–weighted distribution profiles over 24 h, especially at concentrations lower than 10^–8^ M, which indicates populations of stable particles. In the DMEM culture medium for *in vitro* assays, strong aggregation of glycopolymers was observed due to its increased ionic strength, in contrast to water or the low-ionic-strength HBSS solution (Table S3). For more details, see the Supporting Information Section 4.2.

Therefore, for the fluorescence labeling and the *in vivo* biodistribution experiment, we chose to work with the copolymer backbones based on **10**, which had a higher LCST after conjugation with **8** and therefore better solubility in aqueous media compared to **19**. Modification of **10** by the fluorescent label Cy3-azide *via* CuAAC (Table S1) proceeded quantitatively at a mild temperature (45 °C), and the microwave irradiation significantly accelerated the reaction (1 h). The quantitative conversion of CuAAC and the molar content of the fluorescent label were confirmed by ^1^H NMR. Once the fluorescent label was attached, the labeled copolymers **13, 14**, and **15** could not be characterized by SEC-MALLS as they exhibited artifact molecular weights *ca.* 16-fold higher than their nonlabeled counterparts (Table 1). During SEC-MALLS measurement, the Cy3 label emitted fluorescence of the same wavelength as the incident laser light, which artificially increased time-averaged scattering intensity by a factor of *ca.* 16. This phenomenon is described in detail in the Supporting Information Section 4.1.

Interestingly, after the substitution, the overall dispersity of the fluorescently labeled copolymer **13** decreased to 1.10 due to the purification by gel chromatography during the isolation step as the low-molecular-weight polymers retained on the LH20 stationary phase longer than the product. The fluorescently labeled copolymer **13** was further modified with carbohydrate azides **5** and **8** to produce fluorescent glycopolymers **14** (Lac) and **15** (Cou) with 5 mol % content of carbohydrate ligands (Figures S13–S18).

### Affinity of Glycopolymer Inhibitors to Gal-3

Biolayer interferometry was used to evaluate the binding affinity of the prepared glycopolymers. This label-free optical method, utilized for analyzing biomolecular interactions, examines the interference pattern arising from white light reflected off two surfaces: an immobilized protein layer and an internal reference layer. In this study, an *in vivo* monobiotinylated AVI-tagged Gal-3 construct was immobilized on a biosensor via the biotin–streptavidin interaction. Recombinant Gal-3-AVI was produced in *E. coli* BL21 (λDE3) following the previously established protocols.^16,22^ The obtained kinetic data were analyzed by steady-state analysis, and the equilibrium dissociation constant *K*_D_ was extracted. For comparison, the data were fitted to the one-to-one Langmuir kinetic model that presumes the same affinity of all carbohydrate ligands on the glycopolymer, *i.e*., a high homogeneity of the multivalent compound. The evaluation of lactosylated glycopolymers **11** and **17** revealed satisfactory overlaps with the experimental curves. However, for the highly lactosyl-loaded glycopolymer **18** and the coumaryl-loaded glycopolymers **12** and **19**, the fits were less satisfactory. This may have been caused by their nonideal binding behavior attributed either to hydrophobic stacking (in the case of planar coumaryl cores with a high π-electron density) or statistical rebinding on the closely adjacent lactosyl ligands (in the case of highly lactosylated **18**). Hence, for these reasons, we consider the steady-state analysis as the more robust evaluation method, especially for coumaryl-loaded glycopolymers. The kinetic data obtained from both evaluation methods are listed in Table 2 and Figures S32 and S33. The strongest interactions were observed with coumaryl-loaded glycopolymers (**19**, *K*_D_ = 0.40 ± 0.01 nM; **12**, *K*_D_ = 0.85 ± 0.2 nM) and the highly lactosylated glycopolymer **18** (*K*_D_ = 0.48 ± 0.02 nM). Relatively weaker interactions were found with the lactosylated glycopolymers **11** (*K*_D_ = 3.16 ± 0.1 nM) and **17** (*K*_D_ = 4.6 ± 0.1 nM). Notably, there were no significant differences in affinity between both polymer backbones in the copolymer and terpolymer configuration.

**Table 2:** Kinetic Parameters of Interaction of Glycopolymers with Gal-3-AVI

	steady-state	Langmuir 1:1 kinetic model		
compound	*K*_D_ × 10^–9^ [mol·L^–1^]	*k*_a_ × 10^6^ [L.mol^–1^.s^–1^]	*k*_d_ × 10^–4^ [s^–1^]	*K*_D_ × 10^–9^ [mol·L^–1^]
**11** (Lac)	3.16 ± 0.1	3.4 ± 0.7	3.9 ± 1.1	1.1 ± 0.7
**12** (Cou)	0.85 ± 0.2	2.4 ± 1.0	5.8 ± 2.1	0.23 ± 0.1
**17** (Lac)	4.6 ± 0.1	1.2 ± 0.5	16.3 ± 10	1.35 ± 0.4
**18** (Lac-high)	0.48 ± 0.02	1.45 ± 0.4	2.13 ± 0.8	0.15 ± 0.09
**19** (Cou)	0.40 ± 0.01	4.98 ± 2.2	18.1 ± 4.4	0.36 ± 0.04

### Rat Model of Hypoxic Pulmonary Hypertension

Rats exposed to intermittent hypobaric hypoxia developed pulmonary hypertension and right ventricular hypertrophy compared with the control group kept at normoxia (Figure S34). Tissue was collected from the right ventricle and lungs, and the expression of Gal-3, smooth muscle contractile proteins α-actin and calponin, and the extracellular matrix protein collagen I was detected. PCR results and western blot showed an increase in all of the above proteins in both cardiac and lung tissue (Figure S35), although the hydroxyproline assay showed no significant difference in the total collagen content between hypoxic and normoxic tissues. These data support the role of Gal-3 in the pathogenesis of the disease and also suggest a direct involvement of collagen deposition and overexpression of contractile proteins in the pathophysiological changes of lungs and myocardium during pulmonary hypertension. Increased Gal-3 production in pulmonary hypertension has been demonstrated by us and in previous studies.^6,12,36^

### Effect of Gal-3 Inhibitors on Rat Cardiac Fibroblasts and Pulmonary Artery Smooth Muscle Cells *In Vitro*

The results of the analysis of tissues from a rat model of pulmonary hypertension prompted us to test the synthesized polymers in *in vitro* cultures of cardiac fibroblasts and pulmonary artery smooth muscle cells (PASMCs) of rats suffering from pulmonary hypertension. The cells were isolated by explantation and subsequently cultured under hypoxic conditions with a 2.5% O_2_ atmosphere.

**Figure 1:**
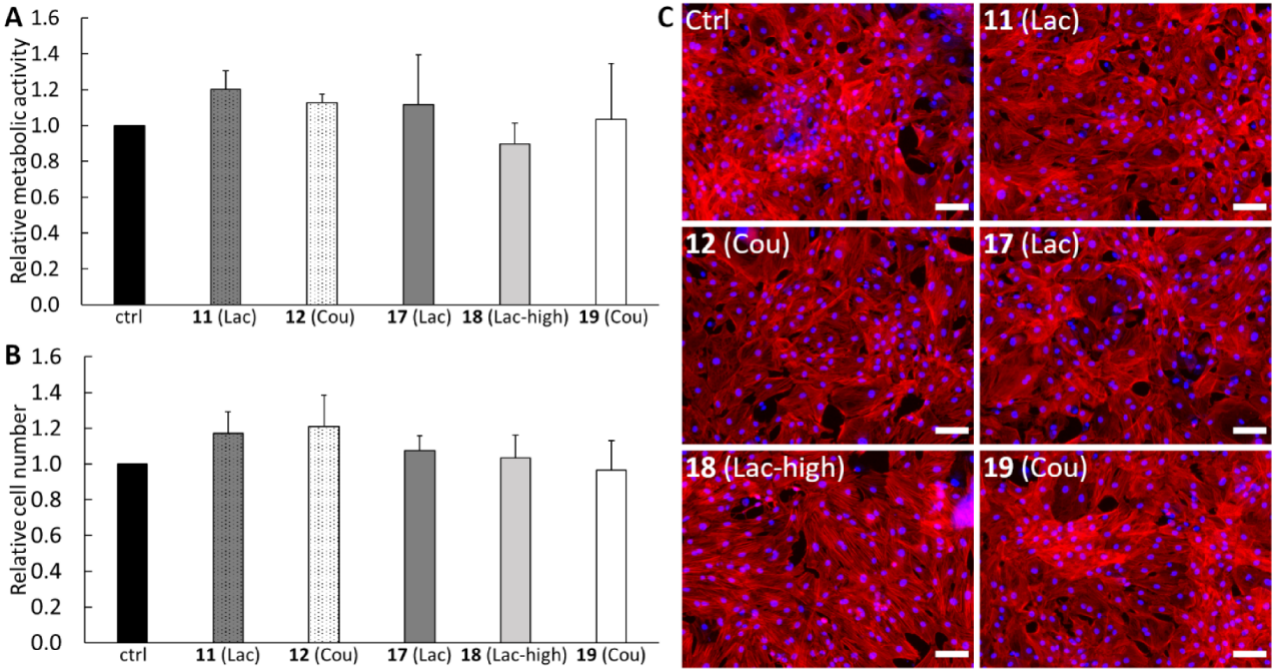
Glycopolymers do not affect cell proliferation. Metabolic activity (A), cell number (B), and fluorescence images (C) of hypoxic rat cardiac fibroblasts treated with polymers. Metabolic activity (A) and cell number (B) were evaluated in cultures on day 3 after the addition of polymers into the cell culture medium. Mean + SD from three independent experiments. One-way ANOVA. No significant difference compared with control was detected (*p* ≤ 0.05). Values were normalized to the control sample in every experiment. Microphotographs of hypoxic rat cardiac fibroblasts (C) stained for F-actin cytoskeleton (red) and nuclei (blue). The scale bar represents 100 μm.

In previous studies, PEtOx and PMeOx polymers of various molecular weights have been shown to be cytocompatible and nontoxic.^37,38^ Our first aim was to verify that the prepared POx polymers conjugated with Gal-3 ligands are not cytotoxic in the cultures of cardiac fibroblasts. Copolymer precursor **10** composed of 2-ethyl-2-oxazoline monomers and terpolymer precursor **16** composed of 2-ethyl-2-oxazoline and 2-methyl-2-oxazoline monomers were used for these experiments. On day 3 after the addition of the glycopolymers to the culture medium (at a final concentration of 100 μM), the metabolic activity of the cells was detected and the number of cells was also determined by direct counting of cell nuclei. The results show that neither lactosyl-loaded **11** and **17** nor coumaryl-loaded **12** and **19** had a negative effect on the cell number and metabolic activity. The increase in the molar content of the carbohydrate portion (**17** – 4 mol % Lac, **18** – 8 mol % Lac) in the terpolymer had no significant effect on the metabolic activity or the cell number either (Figure 1).

**Figure 2:**
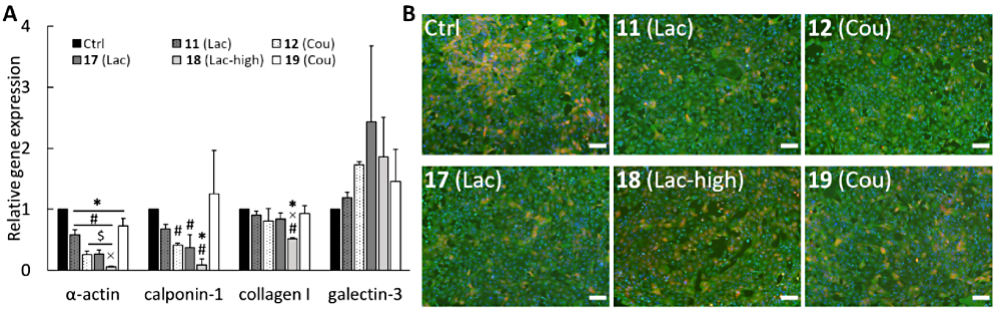
Effect of glycopolymers on the expression of markers related to fibrotic processes of pulmonary hypertension in hypoxic rat cardiac fibroblasts *in vitro*. The expression of α-actin, calponin-1, collagen I, and Gal-3 was evaluated at the mRNA level by qPCR (day 1 after adding polymer, A) and at the protein level by immunofluorescence staining of smooth muscle α-actin (green) and calponin-1 (red). Cell nuclei (blue) (day 3 after adding polymer, B). Mean + SD from three independent experiments. One way ANOVA, Student–Newman–Keuls test. * significant difference compared to Ctrl, $ significant difference compared to **11** (Lac), × significant difference compared to **17** (Lac), # significant difference compared to **19** (Cou); (*p* ≤ 0.05). The scale bar represents 200 μm.

Pharmacological inhibition of Gal-3 by carbohydrate ligands can suppress the expression of specific proteins associated with the pathophysiological remodeling of cardiac and pulmonary artery tissues in pulmonary hypertension.^6,12^ The next step was to investigate the biological activity of the inhibitors in the respective primary cell cultures *in vitro*. For these purposes, we monitored the expression of smooth muscle α-actin and calponin-1 (proteins of the smooth muscle contractile apparatus associated with fibroblast-to-myofibroblast activation and markers of differentiation/phenotypic maturation in smooth muscle cells), collagen I (a marker of tissue fibrotization), and the pro-fibrotic Gal-3 in cell cultures. Expression assays by PCR and immunofluorescence staining (Figure 2) showed that the addition of lactosyl-loaded copolymer **11** into the cell culture medium led to a decrease in the expression of α-actin and calponin by 42% and 32%, respectively. The coumaryl-loaded copolymer **12** showed to be more potent, reducing the α-actin and calponin expression by 74% and 59%, respectively. Both copolymers did not affect collagen I expression and slightly elevated the expression of Gal-3 in cell cultures. After the treatment with lactosyl-loaded terpolymer **17**, a larger decrease in α-actin and calponin-1 expression could be observed (decrease by 73% and 62%, respectively) than that with its copolymer counterpart **11**. Increasing the lactosyl content in the polymer molecule (**18**; Lac-high) caused further amplification of the observed inhibitory effect (decrease in α-actin and calponin-1 expression by 94% and 91%, respectively). In the case of collagen I, only the more efficient **18** (Lac-high) caused a decrease in its expression by approximately 50%. On the other hand, both **17** (Lac) and **18** (Lac-high) caused an elevated Gal-3 expression. Similar results with the most potent **18** (Lac-high) were also obtained in PASMCs (Figure S36).

Intriguingly, in contrast to its copolymer counterpart **12** (Cou), coumaryl-loaded terpolymer **19** (Cou) did not significantly alter the expression of these markers. Based on fast aggregation and precipitation of coumaryl-loaded glycopolymers in high-ionic-strength DMEM shown by DLS (Supporting Information Section 4.2) and also observed under the microscope (Figure S37), we repeated the cell assays in low-ionic-strength HBSS solution for both **12** and **19**. The results were comparable in both DMEM and HBSS (Figure S38). Whereas terpolymer **19** (Cou) did not significantly alter protein expression compared to the control, copolymer **12** (Cou) considerably lowered the expression of α-actin and calponin as expected from its potent interaction with recombinant Gal-3 measured by BLI (Table 2). We hypothesize that the difference between **12** (Cou) and **19** (Cou) is given by the different morphology of these polymers—whereas **12** is a purely statistical copolymer, terpolymer **19** probably exhibits a slight gradient nature caused by higher polymerization rates of MeOx monomer compared to EtOx/BuOx.^39^ Terpolymer **19** (Cou) may tend to behave as an amphiphilic molecule where the hydrophobic coumarylmethyllactosyl ligands form a hydrophobic core stabilized by MeOx-rich chain ends, which decreases the glycopolymer interaction with Gal-3 and subsequent effects on marker expression in cell cultures. This phenomenon could have been eliminated by the administration of *ca.* 5% *v*/*v* DMSO, which, however, already had a negative effect on the cell proliferation.

**Figure 3:**
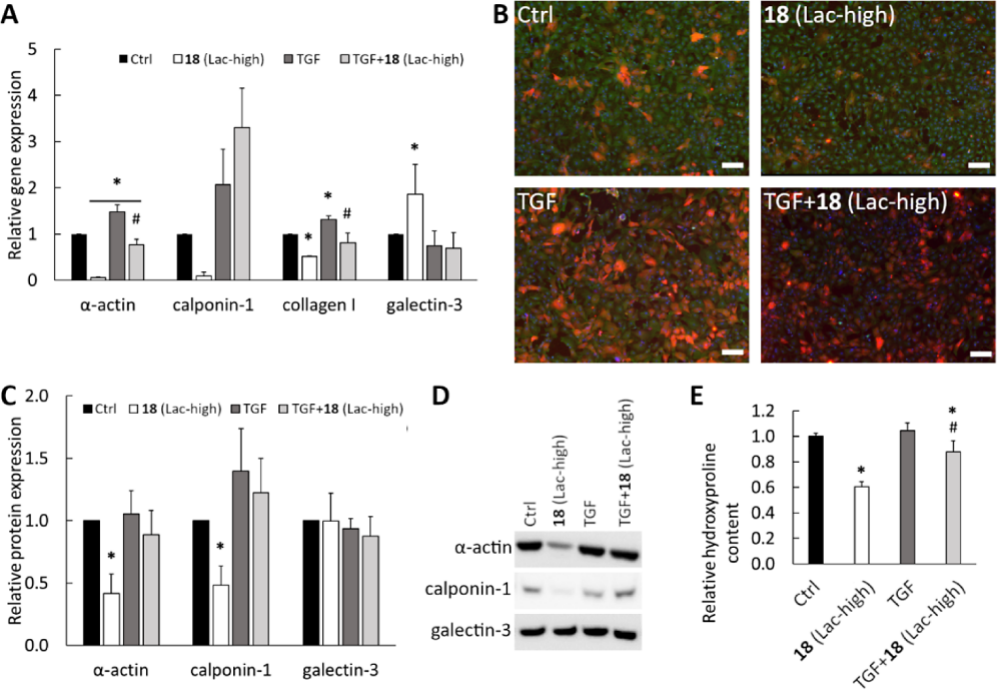
Glycopolymer **18** (Lac-high) partially inhibits TGFβ signaling in hypoxic rat cardiac fibroblasts. **18** (Lac-high) was added to the cell culture medium of unaffected or TGFβ-stimulated cells. The qPCR analysis of gene expression of α-actin, calponin-1, collagen I, and Gal-3 on day 1 after adding the polymer (A). Immunofluorescence staining of smooth muscle α-actin (green) and calponin-1 (red) on day 3 after adding the polymer. Cell nuclei (blue) (B); scale bar represents 200 μm. Western blot analysis of protein expression of α-actin, calponin-1, and Gal-3 on day 3 after adding the polymer (C). Representative picture of western blots (D). Collagen content determined by hydroxyproline assay on day 6 after adding the polymer (E). Mean + SD from three independent experiments. One way ANOVA, Student–Newman–Keuls test. * significant diference compared to Ctrl, # significant difference compared to TGFβ only (*p* ≤ 0.05).

Activation of the TGFβ signaling pathway is a well-known process inducing myocardial and vascular remodeling, which causes activation of fibroblasts into myofibroblasts that express contractile and extracellular matrix proteins responsible for tissue fibrosis.^40^ TGFβ signaling also participates in the development of pulmonary hypertension.^41^ The addition of TGFβ to the cell culture medium resulted in an increased expression of α-actin, calponin, and collagen I, as detected by PCR (Figure 3A) and immunofluorescence staining (Figure 3B). The presence of the most potent glycopolymer **18** (Lac-high) in control cells without TGFβ led to a significant decrease in the expression of α-actin, calponin, and collagen not only at mRNA but also at the protein level, as proved by western blot (Figures 3C,D) and the hydroxyproline assay (Figure 3E). The addition of **18** (Lac-high) to TGFβ-stimulated cells partially suppressed the expression of smooth muscle α-actin, *i.e*., only at the mRNA level, while at the protein level, the suppression was not statistically significant. In contrast, calponin showed a decreased expression only at the protein level, while its expression was even increased at the mRNA level after the addition of **18** (Lac-high) to TGFβ-stimulated cells. These contradictory results indicate that inhibition of Gal-3 cannot completely abrogate the effect stimulated by TGFβ, suggesting that Gal-3 is also involved in other signaling cascades that are TGFβ-independent, such as the Wnt/β-catenin pathway.^8^ Only in the case of collagen, **18** (Lac-high) caused a marked decrease in the expression in TGFβ-stimulated cells as determined both by PCR and the hydroxyproline assay. Analogous experiments were also performed with normoxic cardiac fibroblasts (cultivated under a 21% O_2_ atmosphere) from healthy control rats and hypoxic and normoxic PASMCs. The results from PCR and immunofluorescence staining showed effects identical to hypoxic cardiac fibroblasts (Figure S36).

### Cellular Uptake of Polyoxazoline Polymers

Intracellular uptake is an important aspect that should be considered when designing a new drug delivery strategy. Carbohydrate-based drugs (*i.e*., small hydrophilic molecules) do not usually readily cross the cytoplasmic membrane, resulting in a very low bioavailability and hindering drug action in cells. Cellular internalization of drugs can be enhanced by conjugation with various polymers.^42^ To observe the cellular uptake of the prepared glycopolymers *in vitro* and also their biodistribution *in vivo*, POx carriers were labeled with an orange fluorescent dye Cy3 via an azide linker. Here, 2-ethyl-2-oxazoline copolymer **10** was used. The experiment was aimed to determine whether our glycopolymers penetrate into the cells and whether the internalization is mediated through the binding of the carbohydrate moiety of the polymer to the extracellular Gal-3 on the cell surface.

**Figure 4:**
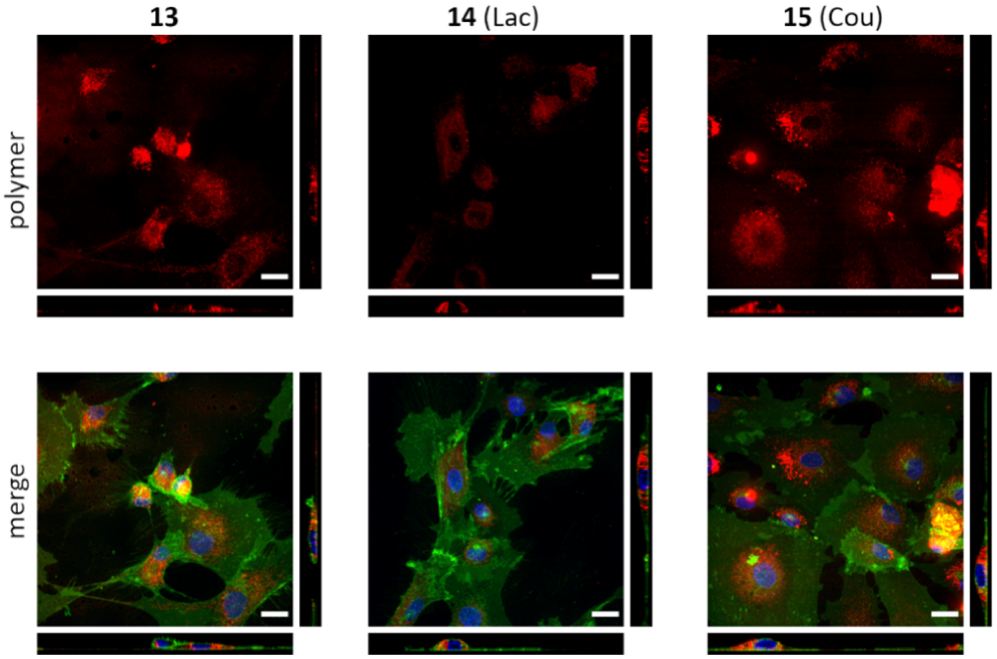
Cellular uptake of POx polymers into hypoxic rat cardiac fibroblasts. Cells were incubated with the respective fluorescently labeled polymer, and after 24 h, confocal images were taken. Polymer—red. Merge—cell membranes were stained with CellMask Deep Red cell membrane staining (green) and nuclei with Hoechst 33324 (blue); fluorescent polymer (red). The scale bar represents 20 μm. Main image: horizontal cell view, right and bottom: vertical cell sections. A Dragonfly 503 scanning disc confocal microscope was used.

The localization of fluorescently labeled polymers **13** (polymer precursor), **14** (Lac), and **15** (Cou) was monitored in cells. Microphotographs taken using a confocal microscope showed cell internalization of all tested polymers (Figure 4), accumulating in the cytoplasm of the cells, especially in the perinuclear region where the cell has a greater thickness. The presence of the polymer was not detected in the nucleus. The polymers penetrated into the cell even when they carried no carbohydrate ligands of Gal-3 (*cf*. precursor **13**), suggesting that cellular uptake is mediated by the physicochemical properties of the POx polymer chain itself, not by the carbohydrate portion. To rule out the possibility that the polymer penetration into cells was mediated by carbohydrate interaction with Gal-3 associated with the cytoplasmic membrane, the cells were incubated with fluorescent polymers in the presence of lactose (100 mM), a well-known Gal-3 inhibitor. As observed on confocal images, the addition of lactose did not prevent the polymers from penetrating into the cells (Figure S39). Our results are consistent with the previously reported POx-mediated internalization of doxorubicin into cancer cells.^43^ Similar to **12** and **19**, we observed the formation of aggregates in the cell culture medium with coumaryl-loaded **15** (Figure S37).

### Biodistribution and Pharmacokinetics Study in Rats

**Figure 5:**
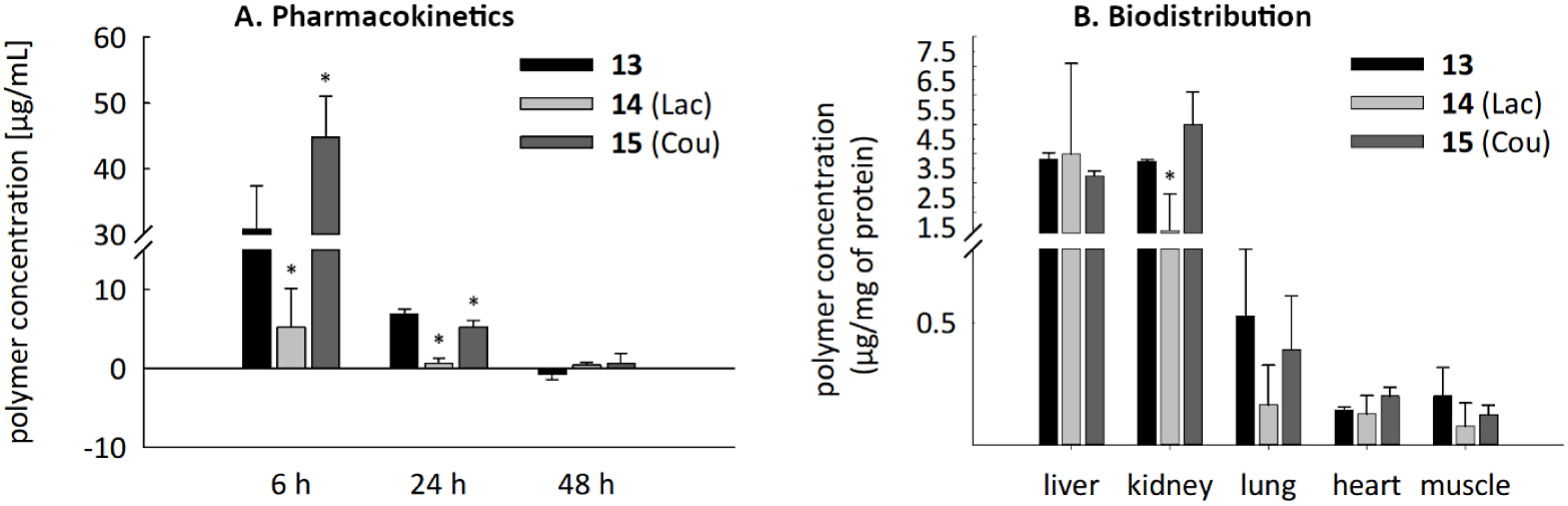
Pharmacokinetics (A) and biodistribution (B) of fluorescently labeled POx polymers in rats. The fluorescence signal was determined in tissue homogenates from rat organs 48 h after peritoneal administration of fluorescently labeled POx polymers conjugated with Gal-3-binding lactosyl (**14**, Lac) or glycomimetic (**15**, Cou). A carbohydrate-free POx carrier without a Gal-3 ligand (**13**) was used as a control. In rat plasma, the signal was detected at 6, 24, and 48 h after administration. Mean + SD, *n* = 3. One way ANOVA, Student–Newman–Keuls test. * significant difference compared to **13** (*p* ≤ 0.05).

One of the disadvantages of carbohydrate-based drugs is their generally short half-life in the systemic circulation, ranging from minutes to hours at most.^44^ For example, glycomimetic TD139, a drug used to treat Gal-3-mediated pulmonary fibrosis, had a half-life of only 8 h.^45^ Low-molecular-weight drugs are usually excreted very rapidly from the bloodstream in the kidneys by passing through the glomerular filtration membranes. A suitable polymeric carrier can significantly improve the bioavailability and circulation time of a drug by increasing its size and molecular weight. The molecular weight threshold for renal filtration for PEGylated drugs ranges between 20 and 50 kDa.^46^ Our Cy3-labeled POx glycopolymers sized *ca.* 10–15 kDa were also tested for their biodistribution and pharmacokinetic properties in a pilot experiment in a rat model. 25 mg of polymer dissolved in a mixture of ethanol/normal saline was administered *i.p.* (*n* = 3) and the concentration of polymer in plasma (6, 24, and 48 h after administration) and in internal organs (48 h after administration) was evaluated (Figure 5). Six hours after polymer administration, the concentration of polymers in plasma ranged from 44 μg·mL^–1^ for **15** (Cou) and 30 μg·mL^–1^ for **13** (sugar-free precursor) to only 5 μg·mL^–1^ for **14** (Lac) (Figure 5A). There was a significant decrease in the polymer concentration in the plasma after 24 h (concentration lower than 7 μg·mL^–1^; **15** (Cou) and **14** (Lac) dropped to approximately 1/8 and **13** (carbohydrate-free precursor) to 1/4 of its concentration at 6 h). By 48 h after administration, the polymers had almost disappeared from the plasma. This points to a relatively fast blood clearance rate of our polymers due to their relatively low molecular weight of 15 kDa, which is below the renal threshold of 40 kDa for PEtOx.^30^ Importantly, however, despite their small size, the conjugation to the POx carrier definitely prolonged the half-life of the carbohydrate-based Gal-3 inhibitors *in vivo*, which confirms the potential of this concept. Notably, coumaryl-loaded glycopolymer **15** showed similar retention rates in blood circulation to POx precursor **13**, which were considerably higher than those for lactosyl-loaded glycopolymer **14**. In the organs, the greatest polymer accumulation was observed in the liver and kidneys (units of μg·mg^–1^ of total protein in the tissue, Figure 5B). To a lesser extent, polymer accumulation also occurred in the lungs, heart, and skeletal muscle. In the lungs, the accumulation of precursors **13** and **15** (Cou) was more pronounced than that of **14** (Lac). In contrast, in the heart tissue, the polymers accumulated in similar concentrations (the coumaryl-loaded **15** may be slightly better). Generally, the lowest concentrations in almost all organs and plasma were detected in the case of the lactosyl-loaded glycopolymer **14**. We hypothesize that the high hydrophilicity of this glycopolymer in combination with its decoration by lactosyl, a carbohydrate commonly recognized by various receptors *in vivo*,^45^ may lead to its faster clearance from the bloodstream and a lower retention in organs.

This is not the case of glycopolymer **15** (Cou) because the coumaryl-derived glycomimetic, in addition to being more hydrophobic, is specifically designed to target Gal-3 and is not expected to be recognized by other human lectins/enzymes.^47^ Moreover, polymers or conjugates of amphiphilic character are known to form micellar nanoparticles in the body, which can then accumulate in some tissues through other mechanisms, such as enhanced permeability and retention effect.^48^ In our case, we observed the formation of aggregates in **12** (Cou), **15** (Cou), and **19** (Cou) in the *in vitro* tests in cell cultures (Figure S37).

## Conclusion

In this work, we have developed a novel targeting system for the potential treatment of pulmonary hypertension based on POx carriers conjugated with carbohydrate-based Gal-3 ligands. Novel POx-based glycopolymers decorated with lactosyl or coumarylmethyllactosyl ligands in different molar proportions were synthesized and characterized. Their affinity and kinetics of binding to Gal-3 were determined by biolayer interferometry, showing high affinities up to the picomolar range. The glycopolymers were tested *in vitro* on the cultures of rat cardiac fibroblasts and pulmonary artery smooth muscle cells isolated from rats suffering from pulmonary hypertension and also from healthy control rats. The cytocompatibility of the glycopolymers, their penetration into cells, and their intracellular localization were demonstrated in cell cultures. The glycopolymers were also shown to strongly suppress the expression of α-actin, calponin, and collagen I, *i.e*., proteins contributing to myocardial and vascular remodeling in pulmonary hypertension. We evaluated the glycopolymers for their pharmacokinetic properties *in vivo* in rats. They not only accumulated mainly in the liver and kidneys, but also in lung and heart tissues, which are most affected by pathophysiological remodeling in pulmonary hypertension.

The combination of these results demonstrated that copolymerization of MeOx with EtOx/BuOx does not achieve the expected increase in LCST for analogous systems and could actually preclude ligand interaction in the case of hydrophobic glycomimetics due to its semigradient nature. The hydrophobicity of the glycomimetic ligand in a molar content as low as 5% also greatly reduced the LCST of polyethyloxazolines. Importantly, however, the results also suggested that the glycopolymer LCST below the body temperature does not necessarily mean that is unsuitable for *in vivo* use. Although **12** (Cou) aggregated in aqueous solutions, it manifested potent Gal-3 inhibition by BLI and significantly reduced *in vitro* gene expression of markers relevant to pulmonary hypertension. Additionally, glycomimetic-loaded **15** (Cou) outperformed soluble lactosyl counterpart **14** (Lac) *in vivo* in terms of its slower clearance rate from blood and higher accumulation in organs.

The present targeting system based on POx carriers appears to be promising for the potential treatment of pulmonary hypertension or other cardiovascular diseases. The *in vivo* biodistribution study indicates the possibility of using the proposed system in clinical applications, considering the tunable pharmacokinetic properties of the polyoxazoline carrier.

## Experimental Section

### Analytical Methods

Detailed experimental procedures for nuclear magnetic resonance (NMR), high-performance liquid chromatography (HPLC), size-exclusion chromatography–multiangle laser light scattering (SEC-MALLS), lower critical solution temperature (LCST) measurements, and dynamic light scattering measurements (DLS) are described in the Supporting Information Section 1. All compounds were >95% pure determined by HPLC/SEC-MALLS.

### β-d-Galactopyranosyl-(1→4)-β-d-glucopyranosyl Azide (**5**)

The synthesis was performed as described previously^49^ (Scheme 1A). Lactose (**1**; 5.6 g, 16.4 mmol) was peracetylated with acetic anhydride (16 mL, 169.3 mmol) in pyridine (10 mL), and the resulting peracetylate **2** was obtained as white powder in 90% yield. After thorough drying, **2** was subjected to bromination by 33% solution of HBr/acetic acid (14 mL) in dry dichloromethane (20 mL), which afforded lactosyl bromide **3** as a white powder in 82% yield. The azide moiety at C-1 was introduced by nucleophilic substitution of **3** in a mixture of dichloromethane (20 mL) and 10% aq. NaHCO_3_ (20 mL) with tetrabutylammonium hydrogensulfate (4.26 g, 12.6 mmol, TBAHS) as a phase-transfer catalyst. The product was extracted and purified on a SiO_2_ column using CH_2_Cl_2_/MeOH/30% aq. NH_4_OH (83:15:2, *v*/*v*/*v*) as a mobile phase. The peracetylated lactosyl azide **4** was obtained as a clear oil in an isolated yield of 77%. Deprotection was performed according to Zemplén, and after lyophilization, the title compound **5** was obtained as a white very hygroscopic powder in 94% yield. The product was analyzed with HRMS, HPLC (95% purity; see the Figures S1 and S2), and NMR; the results were in accord with the literature.^49^

### 3′-*O*-(Coumarylmethyl)-β-d-galactopyranosyl-(1→4)-β-d-glucopyranosyl Azide (**8**)

Compound **8** was synthesized by a modified procedure based on our previous publication^31^ (Scheme 1B). Methyl coumarin **6** was prepared according to Floreková et al.^50^ Anhydrous K_2_CO_3_ (113 mg, 0.89 mmol) was weighed into a 5 mL microwave vial with a magnetic stirrer. The vial was sealed with a rubber septum and was briefly flushed with argon before the addition of 1.61 g (13.2 mmol) of salicyl aldehyde and 2.76 g (21.2 mmol) of propionic anhydride. The reaction mixture was placed in a microwave and heated to 185 °C under medium irradiation for 1 h. Reaction completion was checked by TLC on SiO_2_ (cHex/EtOAc, 9:1 *v*/*v*). The reaction mixture was poured on crushed ice and carefully neutralized with a saturated solution of NaHCO_3_. The resulting yellow chunks were collected and recrystallized from cHex/EtOAc, 3:1, *v*/*v*, yielding **6** as white needle-like crystals (0.88 g, 41%).

Compound **6** was weighed (0.80 g, 4.93 mmol) into a 50 mL round-bottom flask with a magnetic stirrer and dissolved in 20 mL of CH_3_CN. The flask was equipped with a condenser and heated to 85 °C and *N*-bromosuccinimide (NBS; 0.89 g, 5.00 mmol) was added along with 2,2-azobis(isobutyronitrile) (AIBN; 25 mg, 0.13 mmol). The reaction mixture was briefly flushed with argon, sealed with a rubber septum and covered with an aluminum foil. After 8 h, another portion of AIBN (25 mg, 0.13 mmol) and NBS (0.44 g, 2.47 mmol) were added, and the reaction was left overnight. The reaction completion was checked by TLC on SiO_2_ (CH_2_Cl_2_/cHex, 3:1 *v*/*v)*. The reaction mixture was dissolved in 100 mL of CH_2_Cl_2_ and extracted three times with saturated NaHCO_3_ (3 × 100 mL), two times with saturated Na_2_S_2_O_3_ (2× 50 mL), and finally with saturated saline (100 mL). Organic phases were collected and evaporated at reduced pressure, and the residue was purified by column chromatography on SiO_2_ using CH_2_Cl_2_/cHex, 4:1, *v*/*v* as a mobile phase, affording **7** as a white powder (0.77 g, 64%).

Compound **5** (119 mg, 0.32 mmol) was weighed in a 5 mL microwave vial along with 241 mg (0.97 mmol) of Bu_2_SnO_2_ and 104 mg (0.32 mmol) of TBAB, and the vial was sealed with a rubber septum and flushed with argon. The solids were suspended in 3 mL of dry dioxane, and the suspension was heated to 65 °C for 30 min prior to the addition of 280 μL of DIPEA (1.62 mmol). The temperature was increased to 80 °C and left for 2 h, then cooled down, frozen, and lyophilized overnight. To the dry reaction mixture, 234 mg (0.97 mmol) of compound **7** was added, and the microwave vial was sealed with a septum and flushed with argon for at least 5 min. Subsequently, 3 mL of dry dioxane was added and the resulting yellow suspension was placed into a microwave oven for 2 h at 85 °C under medium irradiation. The reaction completion was checked by TLC on SiO_2_ (CH_2_Cl_2_/MeOH/30% aq. NH_4_OH, 89:10:1, *v*/*v*/*v*) and the reaction mixture was evaporated at reduced pressure, adsorbed to a pad of SiO_2_, and purified by column chromatography on SiO_2_ using the same mobile phase as that for TLC. Compound **8** was obtained as a white powder (128 mg, 75%). The product was analyzed with HRMS, HPLC (99% purity), and NMR (see Figures S3 and S4); the results were in accord with the literature.^33^

### Monomer 2-(But-3-yn-1-yl)-4,5-dihydrooxazole (BuOx) (**9**)

The title compound was synthesized according to a published procedure with slight modifications.^34^ Briefly, to the freshly prepared lithium diisopropylamide (25.7 mmol) solubilized in 40 mL of dry THF at −78 °C under an argon atmosphere, 2.10 g (24.7 mmol) of dry 2-methyl-2-oxazoline were added dropwise. The reaction was left stirring for 1 h, followed by a dropwise addition of 2.80 mL (25.0 mmol) of propargyl bromide solution (80% *v*/*v* in toluene) while keeping the temperature under −70 °C. The reaction mixture was left for 2 h at room temperature and was quenched with saturated aq. solution of NH_4_Cl. The aqueous phase was extracted with Et_2_O (4 × 30 mL). The combined organic phases were dried over anhydrous Na_2_SO_4_ and evaporated at reduced pressure. The organic residue was applied on a small pad of silica and eluted with Et_2_O/CH_2_Cl_2_, 1:1, *v*/*v*. During the isolation, it was crucial to use low-boiling solvents, since the product tended to coevaporate with high-boiling solvents (*e.g*., ethyl acetate) and sublimated at ambient temperature at a pressure as high as 400 mbar. Therefore, the resulting product was resublimated twice to afford **9** as white needle-like crystals (32%). ^1^H NMR (700 MHz, CDCl_3_-*d*) δ ppm 1.99 (t, *J* = 2.56 Hz, 1 H) 2.48–2.56 (m, 4 H) 3.84 (t, *J* = 9.60 Hz, 2 H) 4.25 (t, *J* = 9.62 Hz, 2 H). Analysis by HRMS and HPLC (95% purity) is shown in the Figures S5 and S6. The measured data are in agreement with the results published earlier.^34^

### Copolymer Precursor **10**

All glassware and stirrers used for copolymerization were oven-dried, and glassware was further silanized prior to use. First, a 20 mL microwave vial was preheated to 105 °C for at least 20 min and cooled down under a continuous stream of argon. The vial was then loaded with 0.30 g (2.44 mmol) of freshly sublimed BuOx (**9**) and resealed with a rubber septum, flushed with argon for 5 min, and 27 mg of methyl-*p*-toluene sulfonate was added using a stainless-steel syringe. 2-Ethyl-2-oxazoline (1.61 g, 16.2 mmol) was added to the copolymerization feed and was finally dissolved in 3 mL of dry ACN. The copolymerization reaction was heated to 130 °C for 15 min and was terminated by a mixture of 1 M NaOH/MeOH (1:1, *v*/*v*). The termination step was left stirring at 60 °C for 1 h. The copolymerization feed was precipitated into Et_2_O, dried, redissolved in CH_2_Cl_2_, and reprecipitated twice to Et_2_O. The white solid was redissolved in water and was lyophilized overnight. The resulting copolymer **10** was obtained as a glassy white powder (1.33 g, 71%).

### Terpolymer Precursor **16**

The procedure for the synthesis of terpolymer **16** was identical to the copolymer precursor, but with a different molar ratio in comonomer feed, MeOx/EtOx/BuOx/MeOTs, 44:74:15:1, and a shorter polymerization time (12 min). After isolation, terpolymer **16** was obtained as a white glassy powder (0.84 g, 61%).

### Glycopolymers and Labeled Polymers

For coupling of the prepared monovalent azido-functionalized inhibitors **5** or **8** or cyanine 3 azide to the polymeric scaffolds with pendant alkyne groups **10** or **16**, we utilized the Cu^I^ catalyzed alkyne azide cycloaddition (CuAAC) – “click” reaction, using CuSO_4_ as the source of Cu^II^, which were *in situ* reduced by ascorbic acid. All click reactions ran under microwave assistance at 45 °C in DMF at reaction times ranging between 1 and 4 h with an addition of (NH_4_)_2_CO_3_, to prevent the formation of bistriazoles, and unwanted cross-linking of polymer species. The molecular weight characteristics and the degree of substitution of the prepared glycopolymers were evaluated by ^1^H NMR and SEC-MALLS. For all affinity and biological experiments, the *M*_n_ values calculated from NMR evaluation were used (Table 1).

### General Procedure for CuAAC Reaction

The polymer precursor was weighed into a 5 mL microwave vial with a stirrer and dissolved in DMF. To this solution, 0.25 eq (relative to the number of azide groups involved in coupling) of CuSO_4_, followed by 0.25 eq of ascorbic acid were added along with 1 eq of (NH_4_)_2_CO_3_. Finally, 1 eq of the respective azide was added, and the vial was sealed and placed into a microwave reactor. The reaction completion was indicated by the disappearance of the azide spot in TLC. The residual copper ions were quenched by excess quinolinol and the glycopolymer was purified by gel chromatography on Sephadex LH20 (Cytiva Life Sciences) in MeOH/H_2_O, 4:1, *v*/*v,* as a mobile phase. The methanolic solution was evaporated at a reduced pressure, and the residual aqueous solution was lyophilized. Molar amounts of reactants, degrees of substitution, solvent volume, and reaction times are specified in Table S1.

### Affinity of Glycopolymers to Gal-3 – Biolayer Interferometry (BLI)

The affinity of Gal-3-AVI to glyco-copolymers **11** (Lac) and **12** (Cou), and glyco-terpolymers **17** (Lac), **18** (Lac-high), and **19** (Cou) was assessed by biolayer interferometry (BLI) under identical conditions (25 ± 0.1 °C, 850 rpm) using an Octet Red96e interferometry device (FortéBio, Fremont, CA, USA). For kinetic measurements, Gal-3-AVI was diluted to a concentration of 1.8 μg·mL^–1^ in PBS buffer with 0.05% Tween 20 and immobilized on a streptavidin biosensor (Octet SA Biosensors, Sartorius, Goettingen, Germany) through biotin–streptavidin interaction until a spectral shift of 0.6 nm was achieved. Following the Gal-3-AVI immobilization step (150 s), the interactions between Gal-3-AVI and serially diluted glycopolymers (1.95 nM – 2 μM) were monitored for 1050 s during the association (450 s) and dissociation (600 s) phases. It was ensured that the galectin activity remained unaffected by the immobilization on the biosensor. The acquired BLI data were analyzed using Octet Analysis software (FortéBio, Fremont, CA, USA). The nonspecific interaction background (a maximum of 10% of the total response) and the sensor drift for all ligands were subtracted using a double-reference method. Kinetic data for all ligands were evaluated using the Langmuir one-to-one kinetic model and steady-state analysis.

### Experimental Animals

Our model of hypoxic pulmonary hypertension has been described in detail in an earlier study.^51^ Briefly, adult male Wistar rats (200–250 g body weight, *n* = 5) were exposed to intermittent hypobaric hypoxia for 8 h/day, 5 days/week. Barometric pressure (pB) was lowered stepwise so that the level equivalent to an altitude of 7000 m (*p*B = 41 kPa; and *p*O_2_ = 8.6 kPa) was reached after 13 exposures. The total number of exposures was 25. The control normoxic group of animals (*n* = 5) was kept for the same period at pB equivalent to an altitude of 200 m (*p*B = 99 kPa, *p*O_2_ = 20.7 kPa). The study was conducted in accordance with the Guide for the Care and Use of Laboratory Animals published by the US National Institutes of Health (NIH Publication, eighth edition, revised 2011). The experimental protocols were approved by the Animal Care and Use Committee of the Institute of Physiology of the Czech Academy of Sciences (Authorisation to use experimental animals no. 44856/2019-MZE-18134; Prof. Kolář - registration number of the certificate of competence under the Animal Welfare Act no. CZ01823).

### Measurement of Right Ventricular Systolic Pressure

Rats were anesthetized with 2% isoflurane (Aerrane, Baxter, SA, USA) and underwent right ventricular catheterization through the right jugular vein using a curved microtip pressure transducer SPR-513 (Millar, Houston, TX, USA). Data were acquired using the MPVS 300 (Millar, Houston, TX, USA) and PowerLab 8/30 (ADInstruments, Oxford, UK). Right ventricular systolic pressure (RVSP) was averaged from pressure recordings over 3 breathing cycles using LabChart Pro (ADInstruments, Oxford, UK). The animals were then killed by cervical dislocation and their hearts and lungs were excised.

### Isolation and Expansion of Cells from Pulmonary Arteries and Right Ventricle

Vascular smooth muscle cells from the intima-media complex of the pulmonary artery and cardiac fibroblasts from the right ventricle were isolated by an explantation method as described in our earlier study.^52^ Briefly, the tissues from rats exposed to hypobaric hypoxia and control normoxic rats were removed under sterile conditions, cut into small fragments (0.5 mm^3^ or less), and digested by 0.1% collagenase (Worthington) in Dulbecco’s modified Eagle medium (DMEM) at 37 °C for 1 h. The explants were then seeded in plastic flasks (TPP, Trasadingen, Switzerland, the cultivation area of 25 cm^2^; tissue from each animal and of each type in a separate flask) into 2 mL of high-glucose DMEM supplemented with 10% fetal bovine serum (FBS) and gentamicin (40 μg·mL^–1^). The same medium was then used for the cell expansion. The cells isolated from normoxic rats were cultivated under a humidified atmosphere with 21% O_2_ and 5% CO_2_ (the cells are termed normoxic). The cells from rats exposed to hypoxia were cultivated under a humidified atmosphere with only 2.5% O_2_ and 5% CO_2_ (hypoxic cells).

### Cell Seeding

Cell experiments were performed on cell cultures at the second or third passage. Cells were seeded in DMEM with 10% FBS. In the experiments aimed at testing metabolic activity, cell number, immunofluorescence staining, and confocal microscopy, cells were seeded at a concentration of 5000 cells in 200 μL of medium per well in 96-well glass-bottom plates (Cellvis, P96–1.5H–N). For mRNA isolation and subsequent qPCR, cells were seeded at the same concentration in 96-well TCP plates (TPP, cat. no. 92096). For western blotting, cells were seeded at a concentration of 350 000 cells/Petri dish 60 mm in 3 mL of culture medium (Gama Group, cat. no. V400928). For collagen content determination by hydroxyproline assay, cells were seeded at a concentration of 50 000 cells/well in 2 mL of medium on a 12-well culture plate (TPP, cat. no. 92012). To study the effect of Gal-3 inhibitors on cell cultures *in vitro*, 24 h after seeding, samples were replaced with a fresh DMEM medium or HBSS solution containing 2% FBS, or also containing the Gal-3 inhibitor (100 μM) and/or TGFβ1 (10 ng.mL^–1^, Abcam, cat. no. ab50036).

### Biochemical Methods

Detailed experimental procedures for metabolic activity assay, immunofluorescence staining and visualization, isolation of RNA and qPCR, western blotting, hydroxyproline assay, and confocal microscopy are described in Supporting Information Sections 6.1.–6.6.

### *In Vivo* Biodistribution and Pharmacokinetics Study

The normoxic rats were injected intraperitoneally with fluorescently labeled polyoxazoline polymers. Each polymer was injected into 3 rats. The polymers were administered at a concentration of 25 mg·mL^–1^ in a solution of ethanol/normal saline, 1:3, *v*/*v*. At 6, 24, and 48 h after administration, 0.5 mL of blood was collected from the rat tail. Biodistribution was assessed in organs 48 h after polymer administration. Organs from sacrificed rats were frozen and ground in liquid nitrogen followed by Potter-Elvehjem homogenization. Fluorescence intensity in plasma and tissue homogenates was detected in the Synergy HT Multi-Mode Microplate reader. Excitation/emission was set at 530/590 nm. Polymer concentrations were determined from calibration curves constructed by spiking defined concentrations of polymer to tissue homogenates/plasma of control untreated rats. In tissue homogenates, polymer concentration was calculated per mg of total protein as determined by a Pierce BCA protein assay kit (Thermo Fisher Scientific 23227).

### Statistical Analysis

The data are presented as mean + SD if not indicated otherwise. The statistical comparison was made with the use of Student‘s *t* test (*p* ≤ 0.05) or one way ANOVA, Student–Newman–Keuls test, (*p* ≤ 0.05). Statistical analysis was performed in SigmaPlot 14.0 software (Systat Software Inc., USA).

## Supporting Information Available

Additional experimental data, tables, and figures detailing the synthetic procedures, compound characterization, binding assays, and cell culture studies (PDF)

## ABBREVIATIONS

αSMA: α-smooth muscle actin
BLI: biolayer interferometry
Cy3: cyanine3
DIPEA: *N*,*N*-diisopropylethylamine
DMEM: Dulbecco’s modified Eagle medium
DS: degree of substitution
EGTA: egtazic acid
FBS: fetal bovine serum
Gal-3: galectin-3
SEC-MALLS: size-exclusion chromatography–multiangle laser light scattering
HBSS: Hank’s balanced salt solution
HPLC: high-performance liquid chromatography
HPMA: *N*-(2-hydroxypropyl)methacrylamide
HRMS: high resolution mass spectrometry
LCST: low critical solution temperature
NMR: nuclear magnetic resonance
PASMC: pulmonary artery smooth muscle cell
PEO: poly(ethylene oxide)
POx: polyoxazoline
siRNA: small interfering ribonucleic acid
TBAHS: tetrabutylammonium hydrogensulfate
TCP: tissue culture polystyrene
TGFβ: transforming growth factor β
VSMC: vascular smooth muscle cell
