# Supplementary material for: Glycopolymer Inhibitors of Galectin-3 Suppress the Markers of Tissue Remodeling in Pulmonary Hypertension
Source: J Med Chem. 2024 Jun 3;67(11):9214–26. doi: 10.1021/acs.jmedchem.4c00341 (PMC11181325; doi:10.1021/acs.jmedchem.4c00341)
Supplement: Supplementary file 1 — jm4c00341_si_001.pdf [file jm4c00341_si_001.pdf]

Supporting Information

Glycopolymer Inhibitors of Galectin-3 Suppress the Markers of Tissue Remodeling in Pulmonary Hypertension

Antonín Sedlář[†,*], David Vrbata[‡], Kateřina Pokorná[†], Kristýna Holzerová[§], Jakub Červený[‡,#], Olga Kočková[‖], Markéta Hlaváčková[§], Martina Doubková[†], Jana Musílková[†], Vladimír Křen[‡], František Kolář[§], Lucie Bačáková[†] and Pavla Bojarová[‡,⊥,*]

[†] Laboratory of Biomaterials and Tissue Engineering, Institute of Physiology of the Czech Academy of Sciences, Vídeňská 1083, CZ-142 00 Prague 4, Czech Republic
[‡] Laboratory of Biotransformation, Institute of Microbiology of the Czech Academy of Sciences, Vídeňská 1083, CZ-142 00 Prague 4, Czech Republic
[§] Laboratory of Developmental Cardiology, Institute of Physiology of the Czech Academy of Sciences, Vídeňská 1083, CZ-142 00 Prague 4, Czech Republic
[#] Department of Analytical Chemistry, Faculty of Science, Charles University, Hlavova 8, CZ-128 43 Prague 2, Czech Republic
[‖] Laboratory of Analytical Chemistry, Institute of Macromolecular Chemistry of the Czech Academy of Sciences, Heyrovského nám. 1888, CZ-162 00 Prague 6, Czech Republic
[⊥] Department of Health Care Disciplines and Population Protection, Faculty of Biomedical Engineering, Czech Technical University in Prague, nám. Sítná 3105, CZ-272 01 Kladno, Czech Republic

* Corresponding authors: Pavla Bojarová, bojarova@biomed.cas.cz; Antonín Sedlář, antonin.sedlar@fgu.cas.cz

Contents

1. Analytical methods **3**

1.1. Nuclear magnetic resonance (NMR) 3

1.2. High-performance liquid chromatography (HPLC) 3

1.3. Size exclusion chromatography – multi-angle laser light scattering (SEC-MALLS) 4

1.4. Lower critical solution temperature (LCST) measurements 4

1.5 Dynamic light scattering (DLS). 5

2. Synthesis of galectin-3 inhibitors **6**

3. Structural characterization of prepared compounds **8**

4. Behavior of glycopolymers in solution **21**

4.1 SEC-MALLS analysis of fluorescent glycopolymers 21

4.2. DLS characterization of coumaryl-loaded glycopolymers 22

5. Affinity and selectivity of glycopolymers to Gal-3 .... **27**

5.1. Production and purification of recombinant Gal-3 27

5.2. Biolayer interferometry (BLI) 27

6. Biological Results **29**

6.1. Metabolic activity assay 29

6.2. Immunofluorescence staining and visualization 29

6.3. Isolation of RNA and qPCR 29

6.4. Western blotting 30

6.5. Hydroxyproline assay 31

6.6. Confocal microscopy 31

7. References **36**

1. Analytical methods

1.1. Nuclear magnetic resonance (NMR)

NMR spectra of carbohydrate ligands **5** and **8**, and monomer **9** were acquired on a Bruker Avance III 400 MHz spectrometer (Bruker BioSpin, Rheinstetten, Germany) in $CDCl_3$ using standard manufacturer's software. Data were found to be in accord with our previously published characteristics.[1] ^{1}H NMR spectra of polymer compounds **17**, and **19** were measured on a Bruker Avance III 600 (600.23 MHz) spectrometer (Bruker BioSpin, Rheinstetten, Germany) at 30 °C. ^{1}H NMR spectra of polymer compounds **14** and **18** were measured on Bruker Avance III 700 (700.13 MHz) spectrometer at 30 °C. ^{1}H NMR spectra of polymer compounds **10**, **11**, **12**, **13**, **15,** and **16** were measured on Bruker Avance III 400 (399.83 MHz) at 20 °C for **10**, at 5 °C for **13** and **15** or at 30 °C for the remaining samples. All measurements were performed in D_2O and the residual solvent signal was used as the internal standard (δ_H 4.750 ppm). Number molecular weight (M_n) of parent polymers **10** and **16** was calculated by integrating the peak of end-group tosylate anions, which are present in polymer samples even after multiple ether precipitations in the amount proportional to active chain end groups before termination. These signals are easily measurable by ^{1}H NMR since no other signals interfere in the aromatic region for given samples. Therefore, the degree of polymerization *DP* was calculated according to Equation 1:

$$DP = \frac{I_{(3.55)}}{2I_{(7.29)}} \quad (1)$$

where *I* represents the integral of the peak given by the shift in ppm in parentheses. The amount of 2-ethyl-2-oxazoline monomeric units $n_{(\mathrm{EtOx})}$ was calculated according to Equation 2:

$$n_{(EtOx)} = \frac{2I_{(1.09)}}{3I_{(7.29)}} \quad (2)$$

The amount of 2-methyl-2-oxazoline monomeric units in terpolymer precursor **16** was calculated using Equation 2 with the integral of the peak at 2.10 ppm ($I_{(2.10)}$) in the nominator. The amount of 2-butynyl-2-oxazoline monomeric units $n_{(\mathrm{BuOx})}$ was determined by subtraction of these calculated amounts from *DP*. Thus, $n_{(\mathrm{BuOx})} = DP - n_{(\mathrm{EtOx})}$ for copolymer precursor **10** and $n_{(\mathrm{BuOx})} = DP - n_{(\mathrm{MeOx})} - n_{(\mathrm{EtOx})}$ for terpolymer precursor **16**. M_n was calculated by adding all the amounts of monomeric units (*n*) multiplied by the molecular weights of the given monomeric unit. For glycopolymers, the molar content of bound carbohydrate (degree of substitution; *DS*; mol.%) was calculated from the integral ratio of the polymer backbone protons $I_{(3.55)}$ to either the triazole proton $I_{(8.05)}$ or the proton at C1 of the reducing-end carbohydrate moiety $I_{(5.75)}$ as shown in Equation 3:

$$DS = 100\frac{I_{(3.55)}}{4I_{(8.05)}} \quad (3)$$

The degree of substitution was used for calculating the number of carbohydrates per chain and the resulting molecular weight of the carbohydrate ligand portion was added to polymer precursor M_n to obtain the total apparent number average molecular weight M_n of the glycopolymer. These apparent M_n values were in good accordance with GPC-MALLS measurements.

1.2. High-performance liquid chromatography (HPLC)

HPLC analyses were measured on Shimadzu Prominence LC analytical system comprising Shimadzu CBM-20A system controller, Shimadzu LC-20AD binary HPLC pump, Shimadzu

CTO-10AS column oven, Shimadzu SIL-20ACHT cooling autosampler, Shimadzu SPD-20MA diode array detector, and Shimadzu LCMS-2020 mass detector (Shimadzu, Japan). Glycomimetic **8** was analyzed on Chromolith RP-18e column (100×3 mm, monolith) preceded by Chromolith RP-18e guard column (5 × 4.6 mm, Merck, Germany), with gradient elution (A = 5% acetonitrile, 0.1% formic acid, B = 80% acetonitrile, 0.1% formic acid): 0% B for 0-2 min, 0-90% B for 2-7 min, 95% B for 7-8 min, and 90-0% B for 8-11, 11-14 min for column equilibration, flow rate 1.2 mL/min, 25 °C, injection volume 1 μL. Lactosyl azide (**5**) was analyzed on a TSK gel Amide-80 column (250 × 4.6 mm, 5 μm) preceded by TSKgel Amide-80 Guardgel (3.2 × 15 mm, Tosoh corp., Japan) in acetonitrile/water, 4:1, *v*/*v*, with gradient elution as follows (A = acetonitrile, B = water): 22% B for 0-7 min, 22-31% B for 7-16 min, 31-22% B for 16-17 min, and 22% B for 17-22 min for column equilibration; at a flow rate of 1 mL/min at 25 °C; injection volume 1 μL. Diode-array detector data were acquired at 200-380 nm and the maximum signal for the measured compound was extracted. For the analysis of 2-(but-3-yn-1-yl)-4,5-dihydrooxazole (BuOx), we used Luna NH_2 column (150 × 4.6 mm, 5 μm, Phenomenex) equipped with a guard column (NH_2, 4 × 3 mm, Phenomenex), mobile phase 82% acetonitrile, isocratic elution, flow rate 1.1 ml/min, 25 °C, injection volume 0.5 μL. The purity of ligands **5** and **8** used for conjugation to polymer precursors was > 95%, with retention times in accord with our previous work.[1, 2]

1.3. Size exclusion chromatography – multi-angle laser light scattering (SEC-MALLS)

The size-exclusion chromatography (SEC) analyses were performed using a Deltachrom SDS 030 pump (Watrex Ltd., Prague, Czech Republic) with a flow rate of 0.5 mL/min and an autosampler MIDAS (Spark Ltd., Emmen, Holland). Two PLgel 10-μm mixed B LS columns (Polymer Laboratories, Shropshire, UK) were used in a series, each separating according to the producer in the molecular weight range of approximately $5 \times 10^2 \leq M \leq 1 \times 10^7$ (related to PS standards). The following detectors were used: (*i*) a DAWN HELEOS II MALS detector (Wyatt Technology Corp.) with the laser operating at a wavelength λ = 658 nm, and (*ii*) an Optilab T-rEX differential refractometer (Wyatt Technology Corp.), respectively. *N*,*N*-dimethylformamide (≥ 99.9%, for HPLC, Honeywell, Riedel-de-Haën, Germany) with the addition of 0.1M LiCl (95%, Lachema, Czech Republic) was used as a mobile phase at ambient temperature. Data were collected and processed with Astra software (Wyatt Technology Corp.). Dispersity ($Đ$), refractive index increment (d*n*/d*c*), weight average molecular weights (M_w), and number average molecular weights (M_n) were determined from 100% mass recovery of the injected polymer sample. All compounds were > 95% pure by GPC-MALLS analysis.

1.4. Lower critical solution temperature (LCST) measurements

LCST measurements of glycopolymers **12** (Cou) and **19** (Cou) were performed in a thermostated bath in a temperature range of 1-25 °C in 1 °C increments with 5 min equilibration for each reading. The LCST was deducted as visible clouding of the solution at the given temperature. We found LCST to be 17 °C for glycopolymer **12** (Cou) and 8 °C for glycopolymer **19** (Cou). LCST measurements of polymer precursors **10** and **16** were performed in a thermostated bath with a temperature range of 60-100 °C in 1 °C increments with 5 min equilibration for each reading. Visible clouding of the solution was observed at 74 °C for copolymer precursor **10**. No cloud point for terpolymer precursor **16** appeared in the tested temperature range.

1.5 Dynamic light scattering (DLS).

DLS was measured using Zetasizer Nano S90 (Malvern Instruments Ltd., Malvern, UK) at 23 °C. The light scattered at $\theta = 173°$ from the incident light was fitted to an autocorrelation function using the method of cumulants (Malvern Instruments Ltd., Malvern, UK). Sample preparation: 1 mg of polymer **12**, **15**, or **19** was solubilized in 1 mL of deionized water at 4 °C at a starting concentration of 1 mg/mL, of which a two-fold serial dilution row was prepared. The samples were kept at 4 °C and equilibrated to 23 °C (20 min) before acquisition. These samples represent the starting point of the measurement ($t = 0$); another measurement was taken after 24 h of incubation at 23 °C. The samples were not filtered and carefully pipetted from the surface to precisely monitor the dynamics of aggregation and avoid the transfer of possible big sedimenting aggregates. The aggregating polymer solutions were highly polydisperse (PDI = 0.2-0.8) and the data required careful interpretation. All intensity-weighted particle sizing is overestimated by the presence of large sedimenting particles, and therefore it is not reported. As a result, we only monitored the trends based on the intensity-weighted distribution fit of the autocorrelation function and presented them together with autocorrelograms and concentration dependent plot of z-average with polydispersity displayed as error bars. Only the data meeting the criteria of a signal-to-noise ratio between 0.1-1 and of the derived count rate per second higher than 100 c/s (count rate recalculated to attenuation factor) were considered. Fluorescent copolymer **15** (Cou-Cy3) solutions had a too low signal-to-noise ratio promoted by sample fluorescence (Figure S31).

2. Synthesis of galectin-3 inhibitors

Table S1. Synthetic details of CuAAC click reactions.

		Glyco-copolymer **11** (Lac)			
Reactant	*M* [g/mol]	*m* [mg]	*n* [µmol]	*mol. eq*	*V* [µL]
Precursor **10**	$10.6 \cdot 10^3$	75	7.08	1	
Ligand **5**	367	13	35.4	5	
$CuSO_4$ (1.5 M)	250	-	8.90	0.25	6
Ascorbate (1 M)	176	-	8.90	0.25	9
$(NH_4)_2CO_3$	96	3	31.3	4.40	
Solvent: DMF (2 mL), MW irradiation at 45 °C for 2 h, white solid, **90% yield**					
		Glyco-copolymer **14** (Lac)			
Reactant	*M* [g/mol]	*m* [mg]	*n* [µmol]	*mol. eq*	*V* [µL]
Precursor **13**	$10.6 \cdot 10^3$	115	10.8	1	
Ligand **5**	367	20	54.3	5	
$CuSO_4$ (1.5 M)	250	-	13.6	0.25	9
Ascorbate (1 M)	176	-	13.6	0.25	14
$(NH_4)_2CO_3$	96	5	52.1	4.80	
Solvent: DMF (2 mL), MW irradiation at 45 °C for 2 h, pink fluffy solid, **84% yield**					
		Labeled co-polymer precursor **13**			
Reactant	*M* [g/mol]	*m* [mg]	*n* [µmol]	*mol. eq*	*V* [µL]
Precursor **10**	$10.6 \cdot 10^3$	640	60.4	5	-
Cy3-azide label	575	7	12.2	1	-
$CuSO_4$ (1.5 M)	250	-	3	0.25	2
Ascorbate (1 M)	176	-	3	0.25	3
$(NH_4)_2CO_3$	96	2	20.8	1.70	-
Solvent: DMF (8 mL), MW irradiation at 45 °C for 2 h, pink fluffy solid, **85% yield**					
		Labeled glyco-copolymer **15** (Cou)			
Reactant	*M* [g/mol]	*m* [mg]	*n* [µmol]	*mol. eq*	*V* [µL]
Precursor **13**	$10.6 \cdot 10^3$	215	20.3	1	
Ligand **8**	527	69	131	6.45	
$CuSO_4$ (1.5 M)	250	-	32.8	0.25	22
Ascorbate (1 M)	176	-	32.8	0.25	33
$(NH_4)_2CO_3$	96	13	135	1	
Solvent: DMF (3 mL), MW irradiation at 45 °C for 1 h, pink fluffy solid, **88% yield**					
		Labeled glyco-copolymer **12** (Cou)			
Reactant	*M* [g/mol]	*m* [mg]	*n* [µmol]	*mol. eq*	*V* [µL]
Precursor **10**	$10.6 \cdot 10^3$	70	6.60	1	
Ligand **8**	527	17	32.3	5	
$CuSO_4$ (1.5 M)	250	-	8.01	0.25	6
Ascorbate (1 M)	176	-	8.01	0.25	9
$(NH_4)_2CO_3$	96	3	32.3	5	
Solvent: DMF (3 mL)/H_2O (0.3 mL), MW irradiation at 45°C for 1 h, white solid, **88% yield**					
		Glyco-terpolymer **19** (Cou)			
Reactant	*M* [g/mol]	*m* [mg]	*n* [µmol]	*mol. eq*	*V* [µL]
Precursor **16**	$8.7 \cdot 10^3$	72	8.28	1	

Ligand **8**	527	17	33.1	4	
$CuSO_4$ (1.5 M)	250	-	8.28	1	6
Ascorbate (1 M)	176	-	8.28	1	9
$(NH_4)_2CO_3$	96	3	31.8	3.84	
Solvent: DMF (2 mL), MW irradiation at 45 °C for 2 h, white solid, **84% yield**					

Glyco-terpolymer **17** (Lac)					
Reactant	*M*[g/mol]	*m* [mg]	*n* [µmol]	*mol. eq*	*V* [µL]
Precursor **16**	$8.7 \cdot 10^3$	75	8.62	1	
Ligand **5**	367	13	35.4	4.11	
$CuSO_4$ (1.5 M)	250	-	8.85	1.03	6
Ascorbate (1 M)	176	-	8.85	1.03	9
$(NH_4)_2CO_3$	96	3	31.3	3.54	
Solvent: DMF (2 mL), MW irradiated at 45 °C for 2 h, white solid, **86% yield**					

Glyco-terpolymer **18** (Lac-high)					
Reactant	*M* [g/mol]	*m* [mg]	*n* [µmol]	*mol. eq*	*V* [µL]
Precursor **16**	$8.7 \cdot 10^3$	71	8.16	1	
Ligand **5**	367	23	62.7	7.68	
$CuSO_4$ (1.5 M)	250	-	15.7	1.92	10
Ascorbate (1 M)	176	-	15.7	1.92	15
$(NH_4)_2CO_3$	96	6	62.7	7.68	
Solvent: DMF (2 mL), MW irradiation at 45 °C for 1 h, white solid, **93% yield**					

3. Structural characterization of prepared compounds

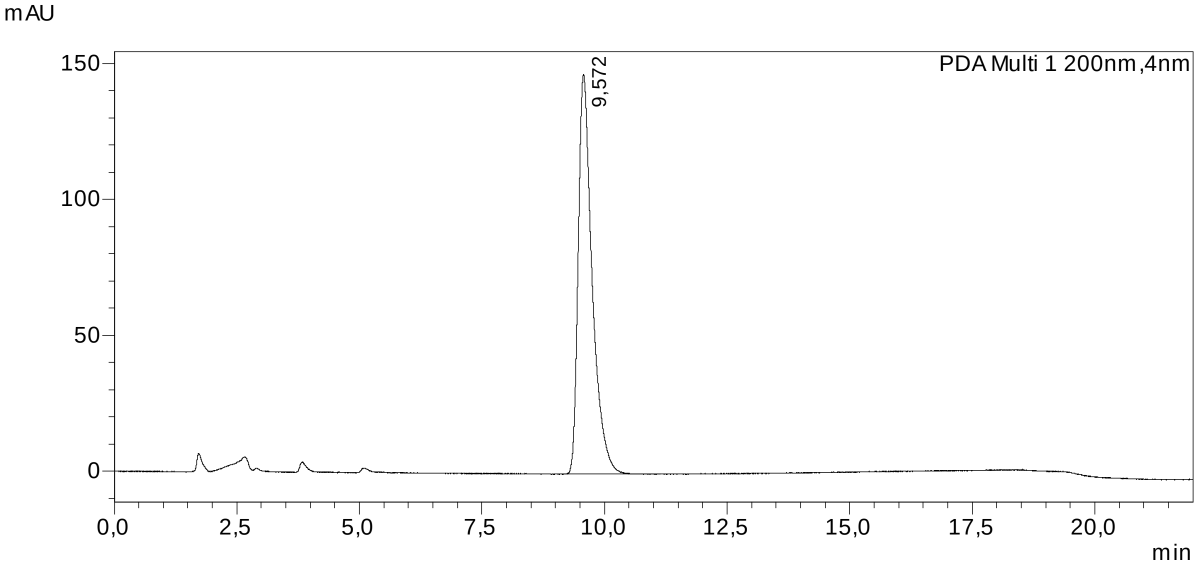

Figure S1. HPLC chromatogram of isolated compound **5** (9.572 min, 95% purity).

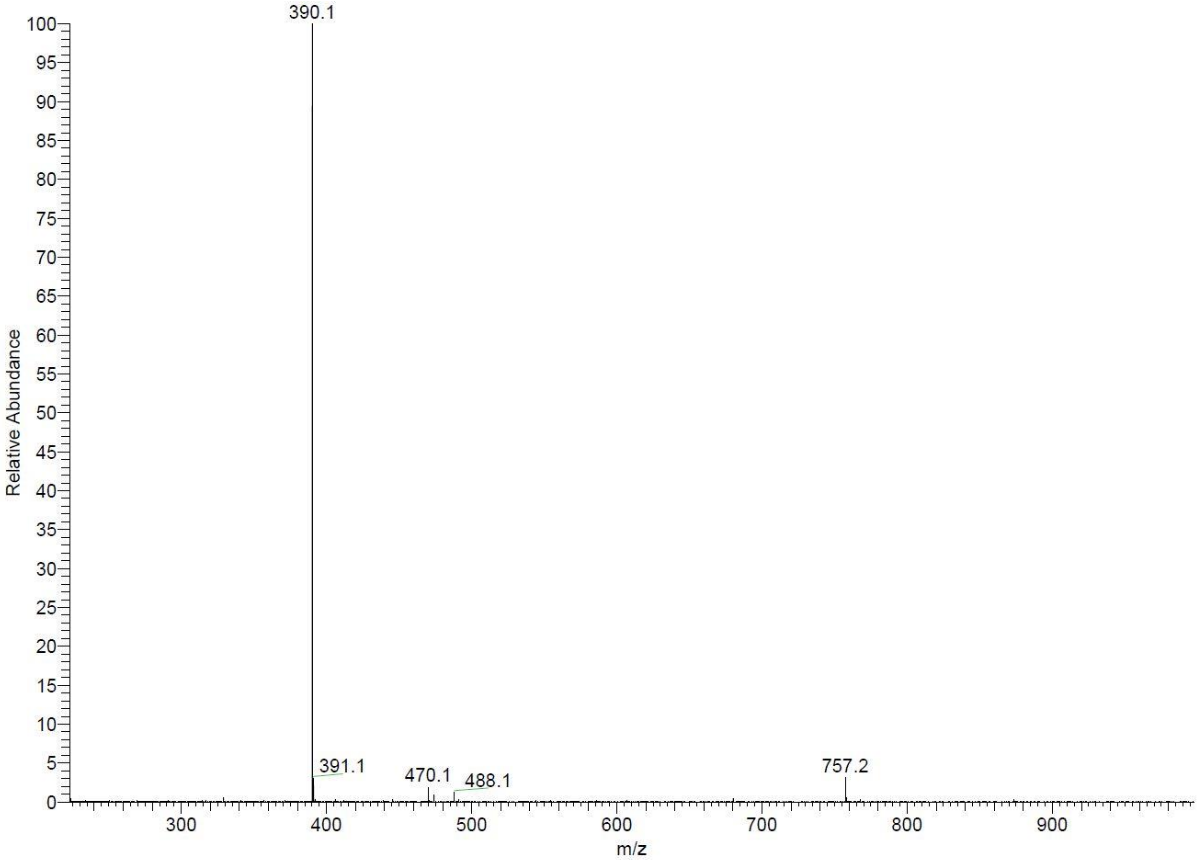

Figure S2. MS spectrum of compound **5** ($[M+Na]^+$, *m/z* 390.1). HRMS (ESI^+): calculated for $C_{12}H_{21}O_{10}N_3Na$ = 390.11191; measured 390.11187 (-0.10268 ppm).

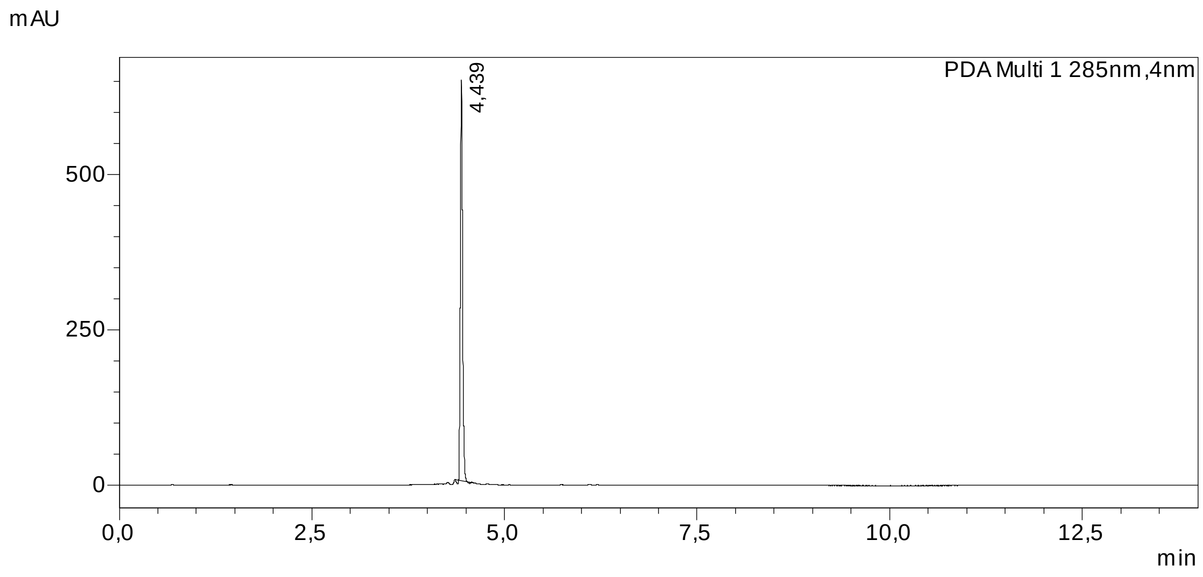

Figure S3. HPLC chromatogram of compound **8** (4.439 min, 99% purity).

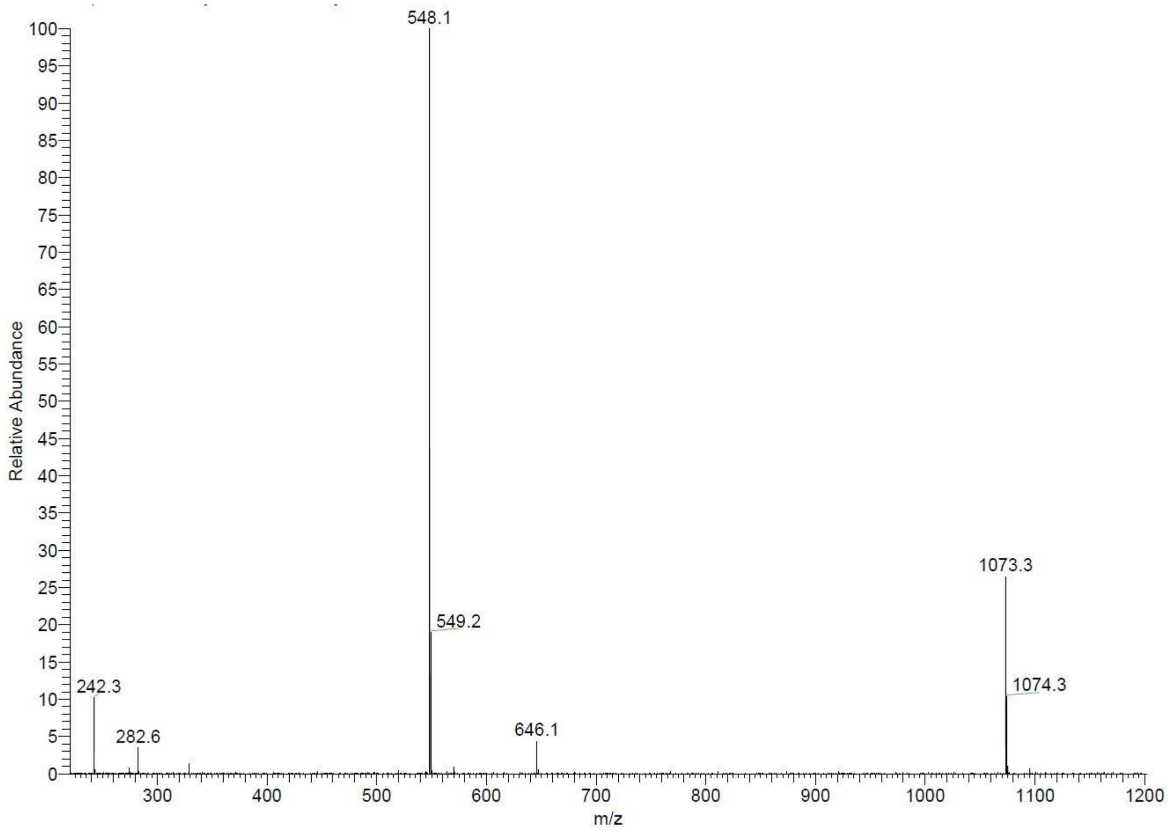

Figure S4. MS spectrum of compound **8** ($[M+Na]^+$, *m/z* 548.1). HRMS (ESI^+): calculated for $C_{22}H_{27}O_{12}N_3Na$ = 548.14869; measured 548.14854 (-0.28078 ppm).

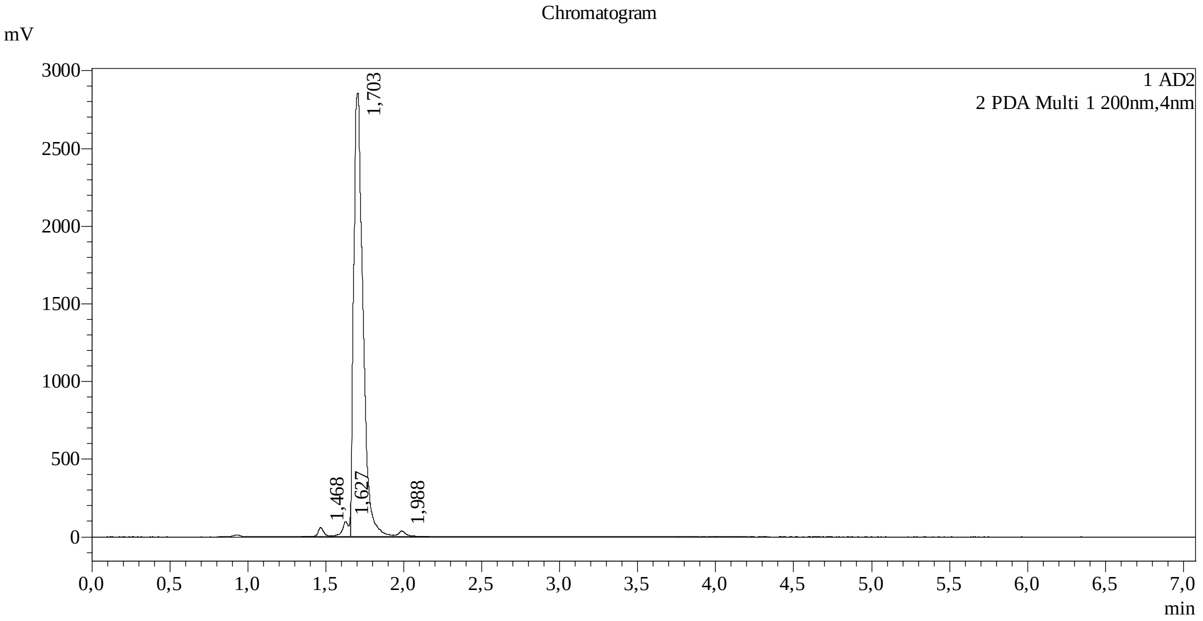

Figure S5. HPLC chromatogram of compound **9** (1.703 min, 95% purity).

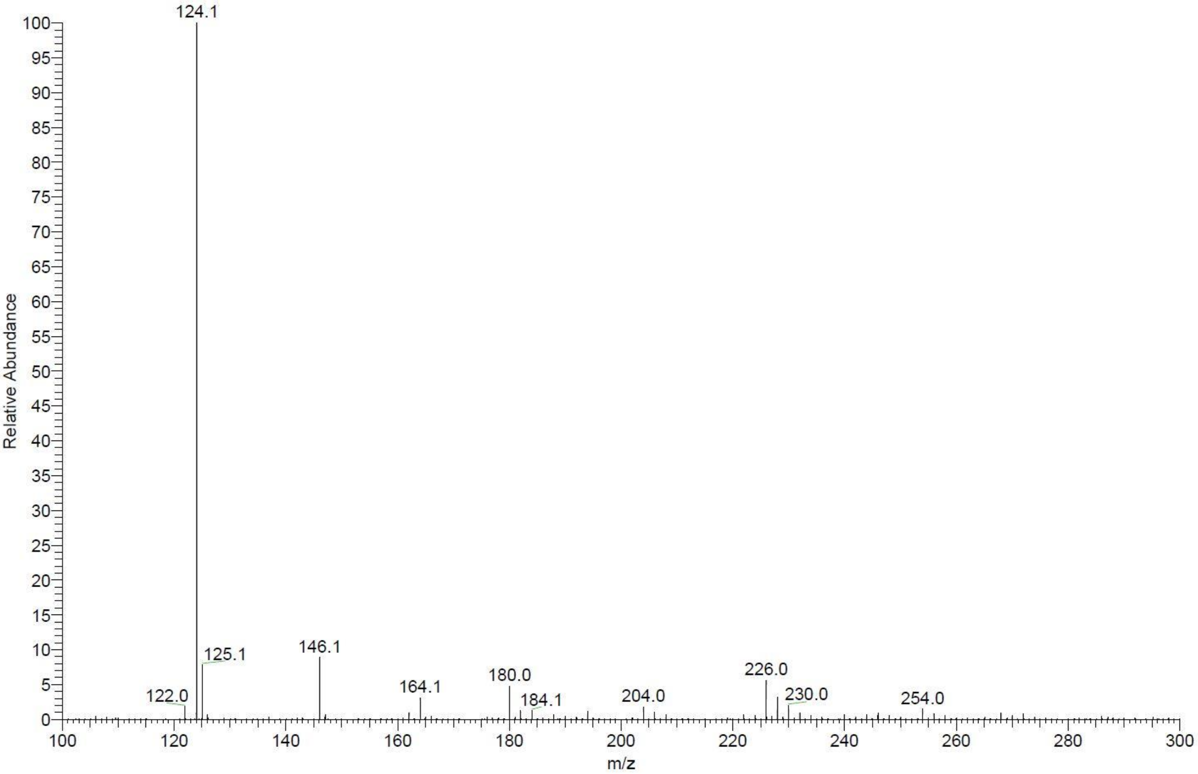

Figure S6. MS spectrum of compound **9** ($[M+Na]^+$, *m/z* 146.1; $[M+H]^+$, *m/z* 124.1). HRMS (ESI^+): calculated for $C_7H_{10}ON$ = 124.07569; measured 124.07556 (-1.06183 ppm).

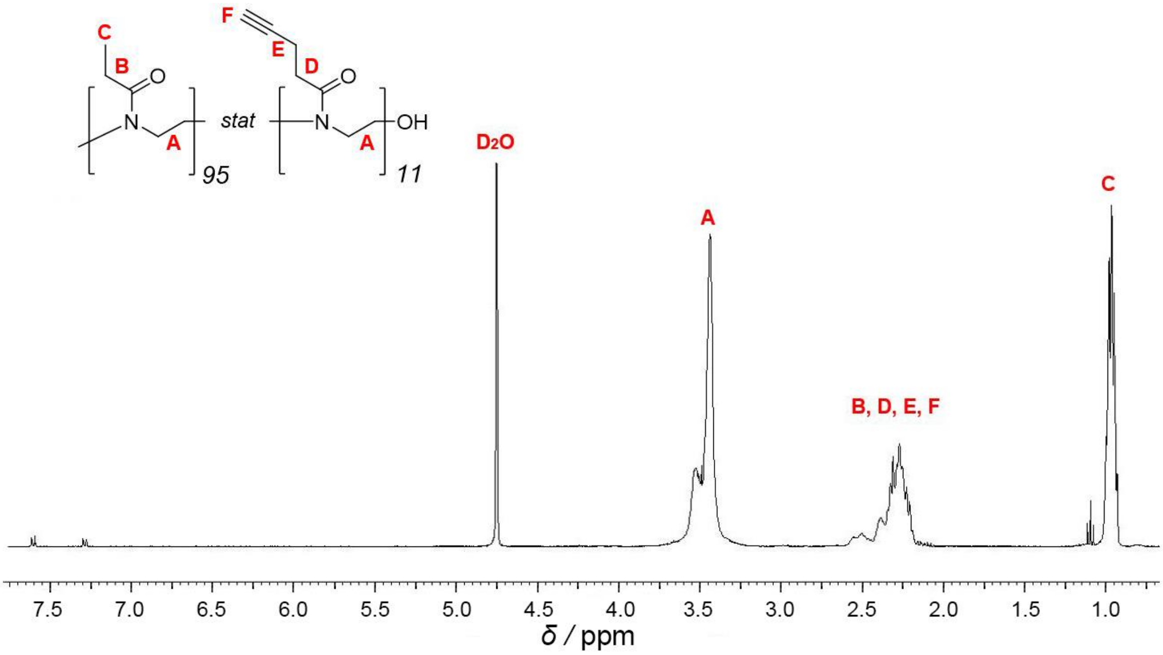

Figure S7. ^{1}H NMR spectrum of copolymer precursor **10**.

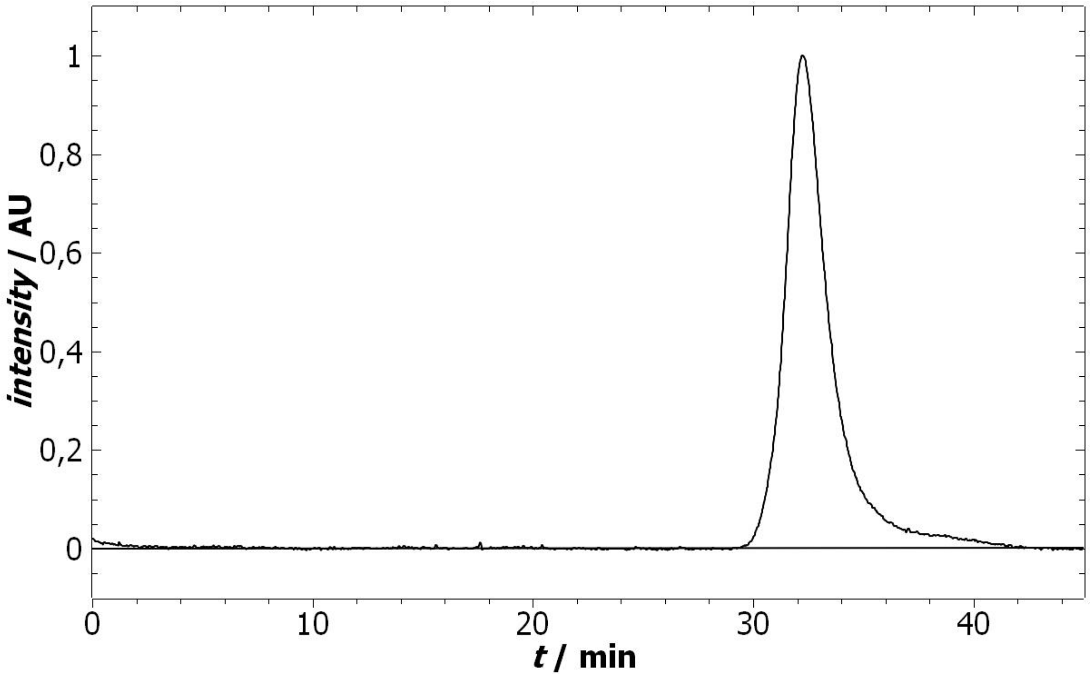

Figure S8. GPC-MALLS chromatogram of copolymer precursor **10**, t_R = 32.26 min, detection by LS.

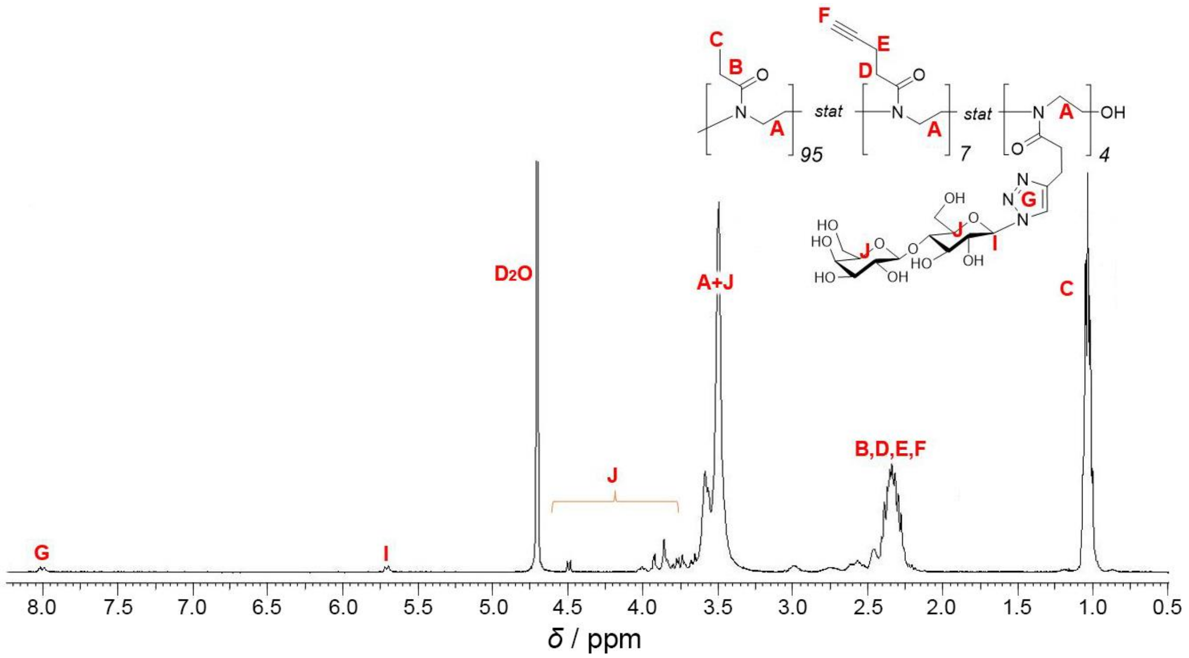

Figure S9. ^{1}H NMR spectrum of glyco-copolymer **11** (Lac).

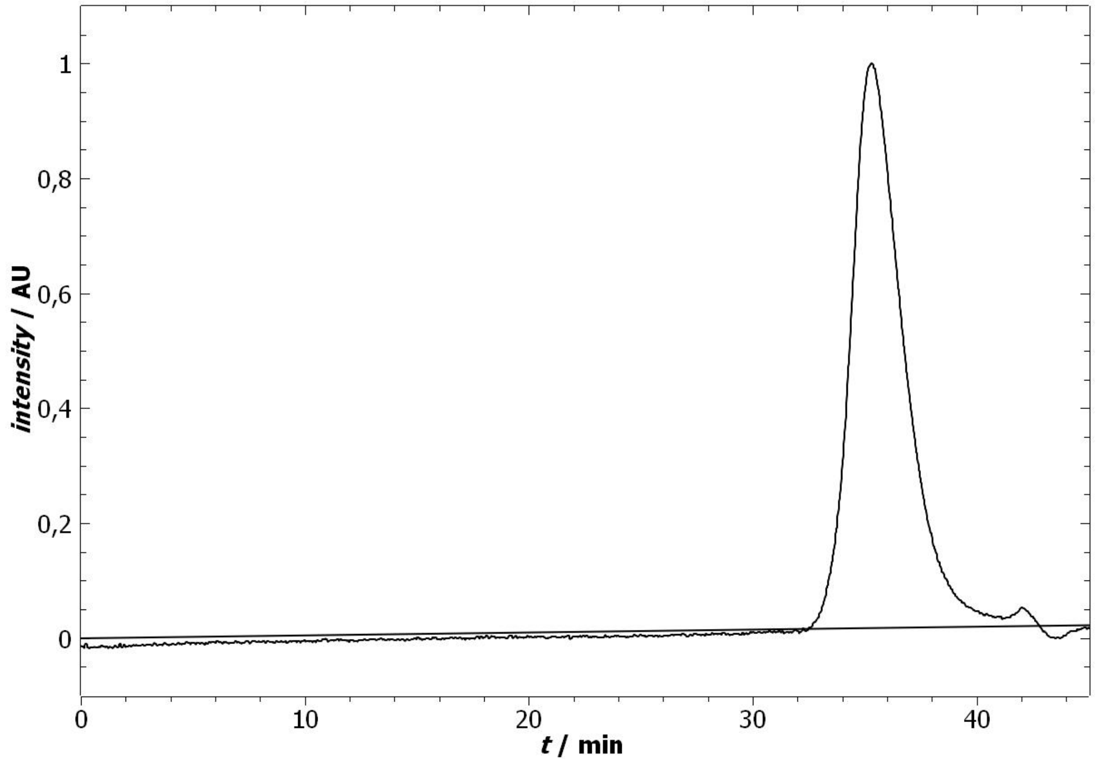

Figure S10. GPC-MALLS chromatogram of glyco-copolymer **11** (Lac), t_R = 35.32 min, detection by LS.

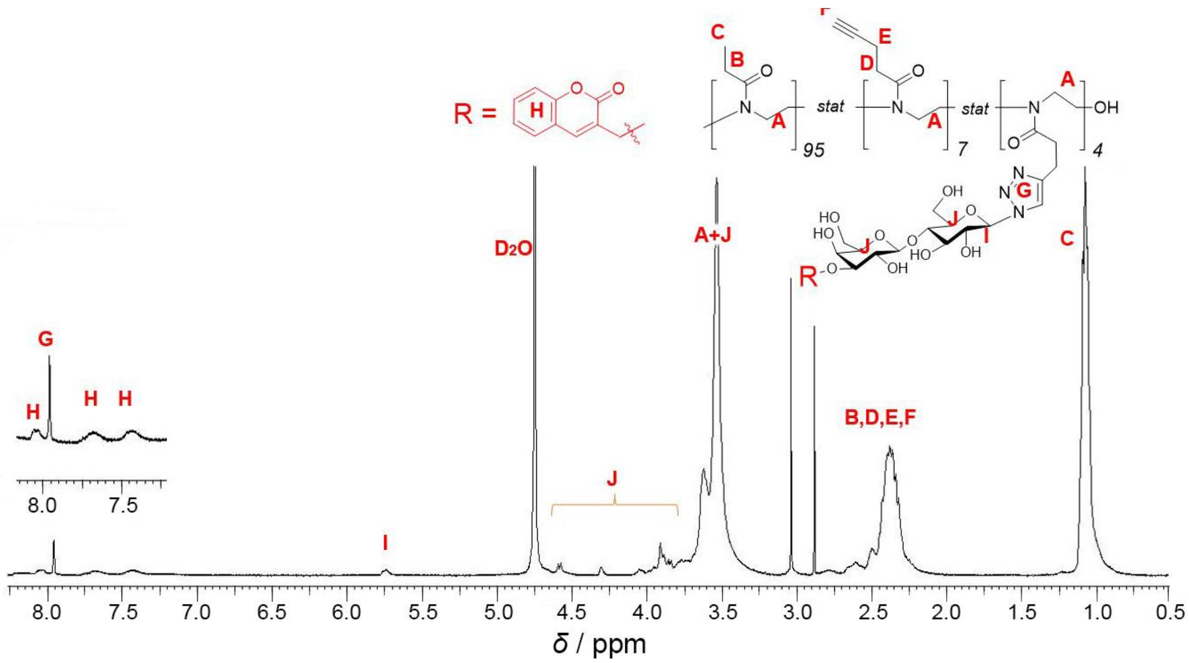

Figure S11. ^{1}H NMR spectrum of glyco-copolymer **12** (Cou).

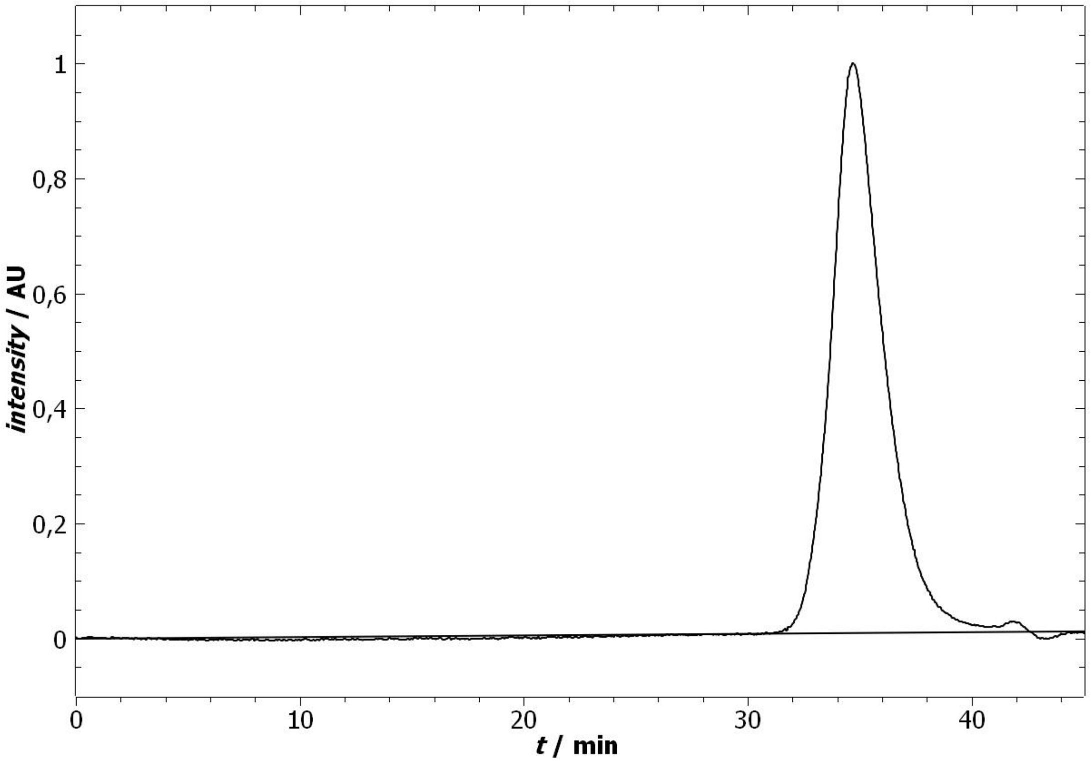

Figure S12. GPC-MALLS chromatogram of glyco-copolymer **12** (Cou), t_R = 34.68 min, detection by LS.

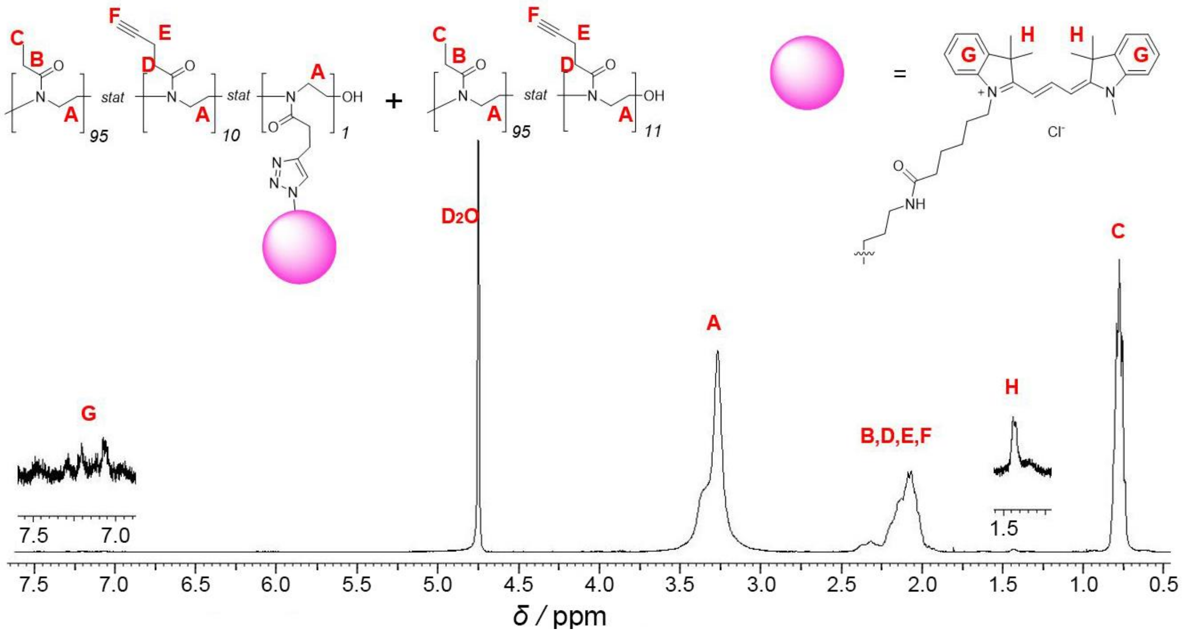

Figure S13. ^{1}H NMR spectrum of labeled copolymer precursor **13**.

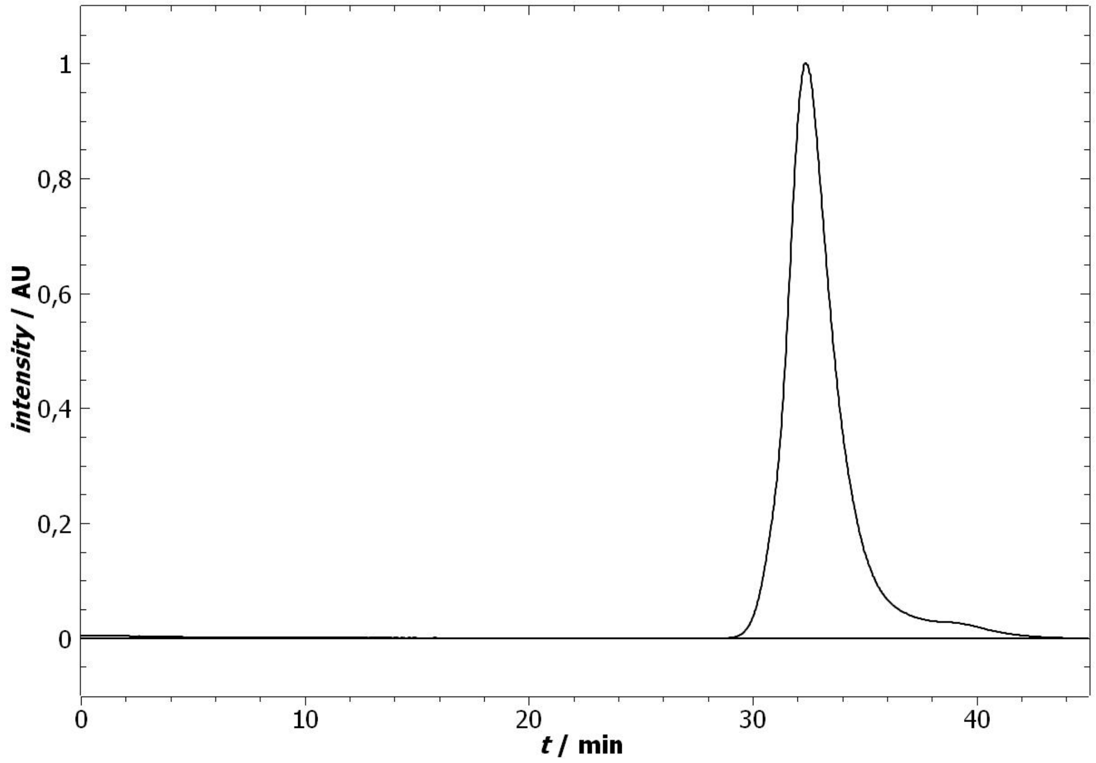

Figure S14. GPC-MALLS chromatogram of labeled copolymer precursor **13**, t_R = 32.26 min, detection by LS.

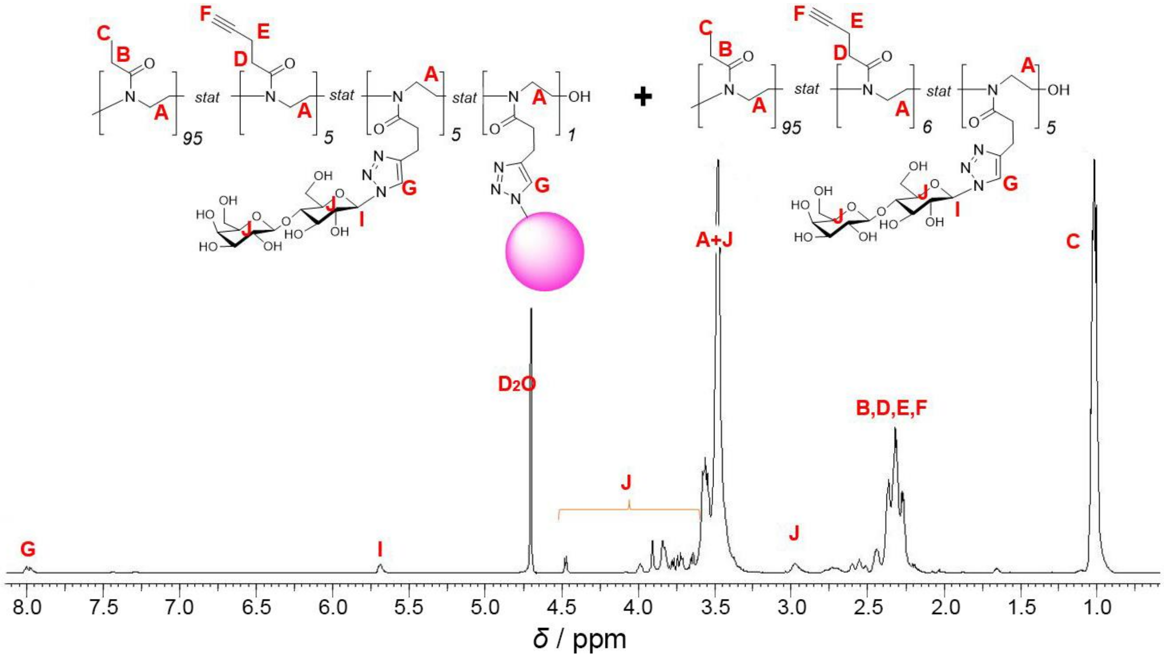

Figure S15. ^{1}H NMR spectrum of labeled glyco-copolymer **14** (Lac).

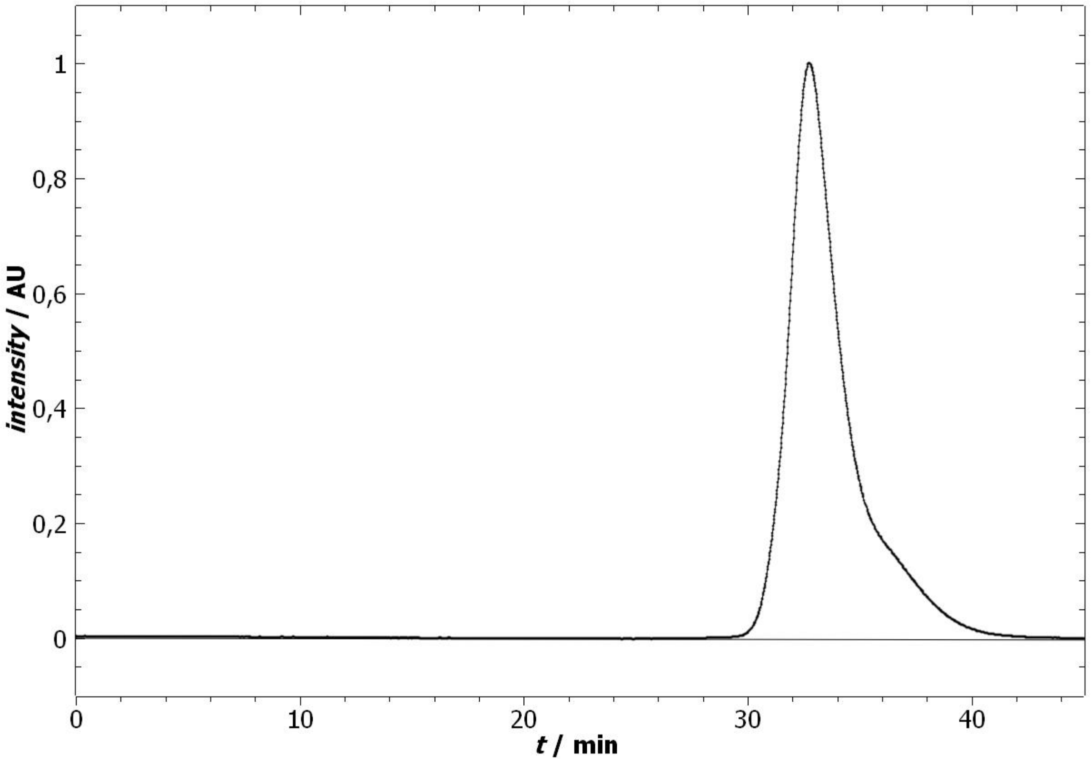

Figure S16. GPC-MALLS chromatogram of labeled glyco-copolymer **14** (Lac), t_R = 32.76 min, detection by LS.

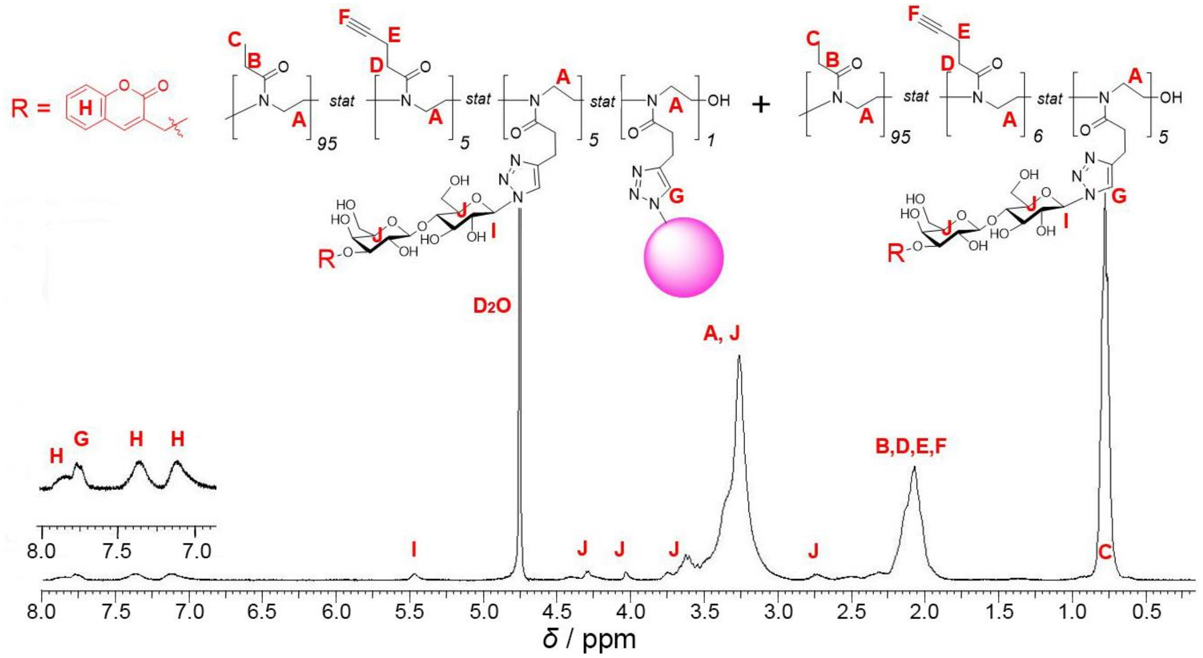

Figure S17. ^{1}H NMR spectrum of labeled glyco-copolymer **15** (Cou).

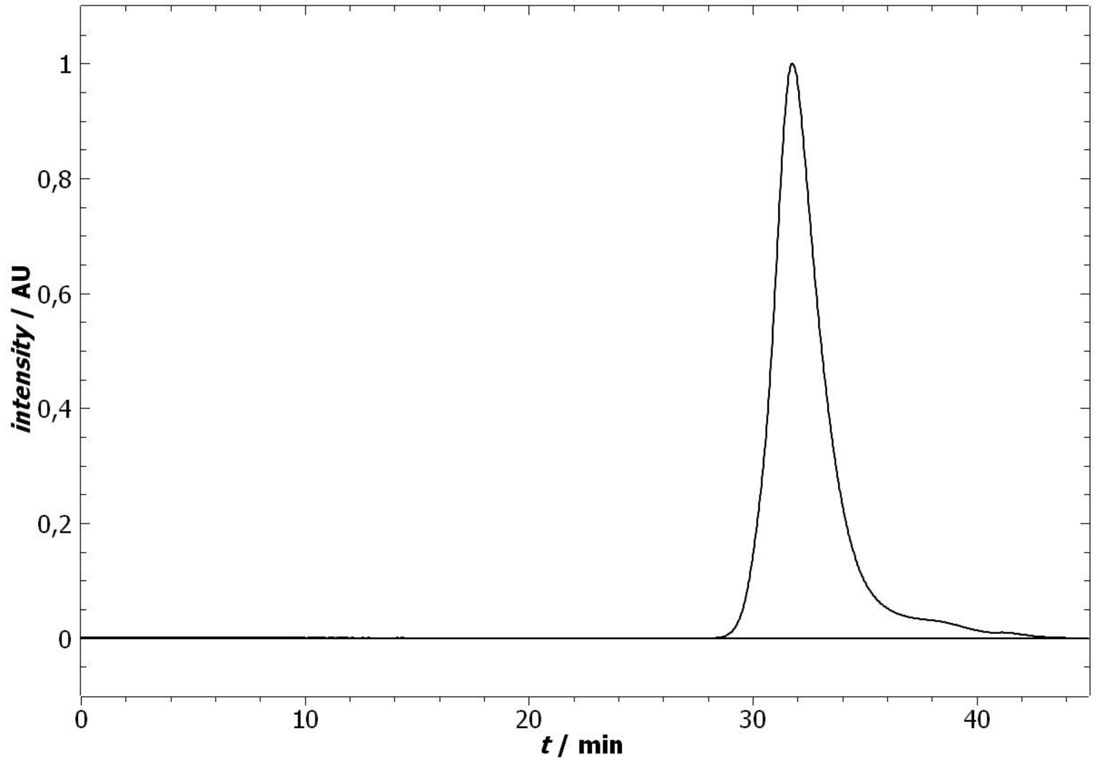

Figure S18. GPC-MALLS chromatogram of labeled glyco-copolymer **15** (Cou), t_R = 31.77 min, detection by LS.

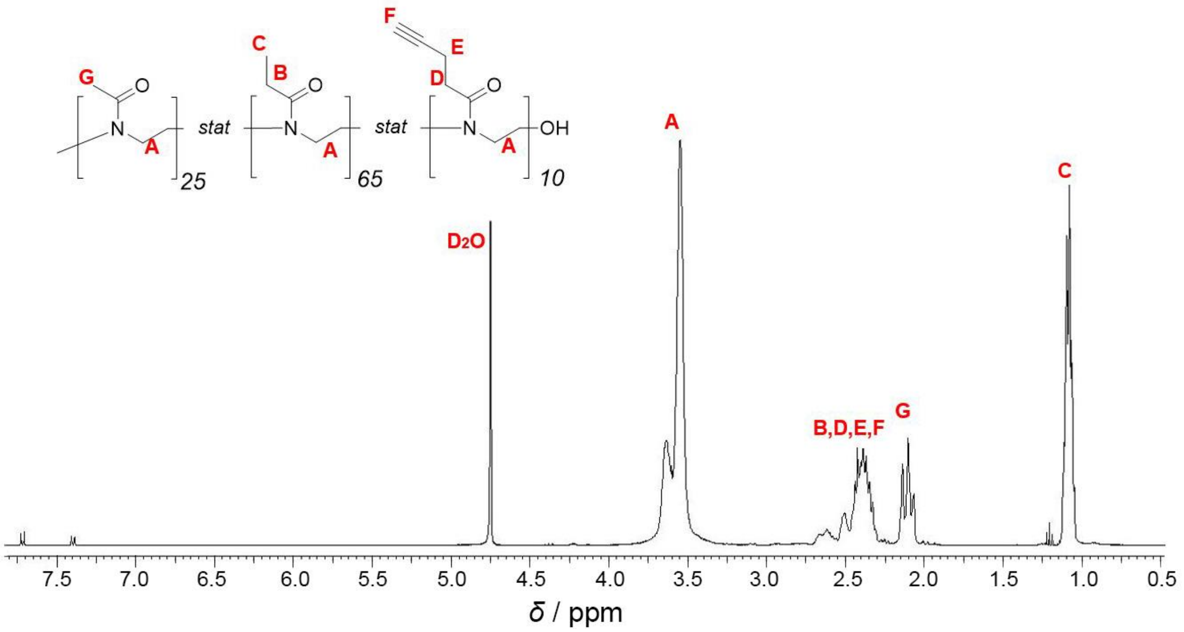

Figure S19. ^{1}H NMR spectrum of terpolymer precursor **16**.

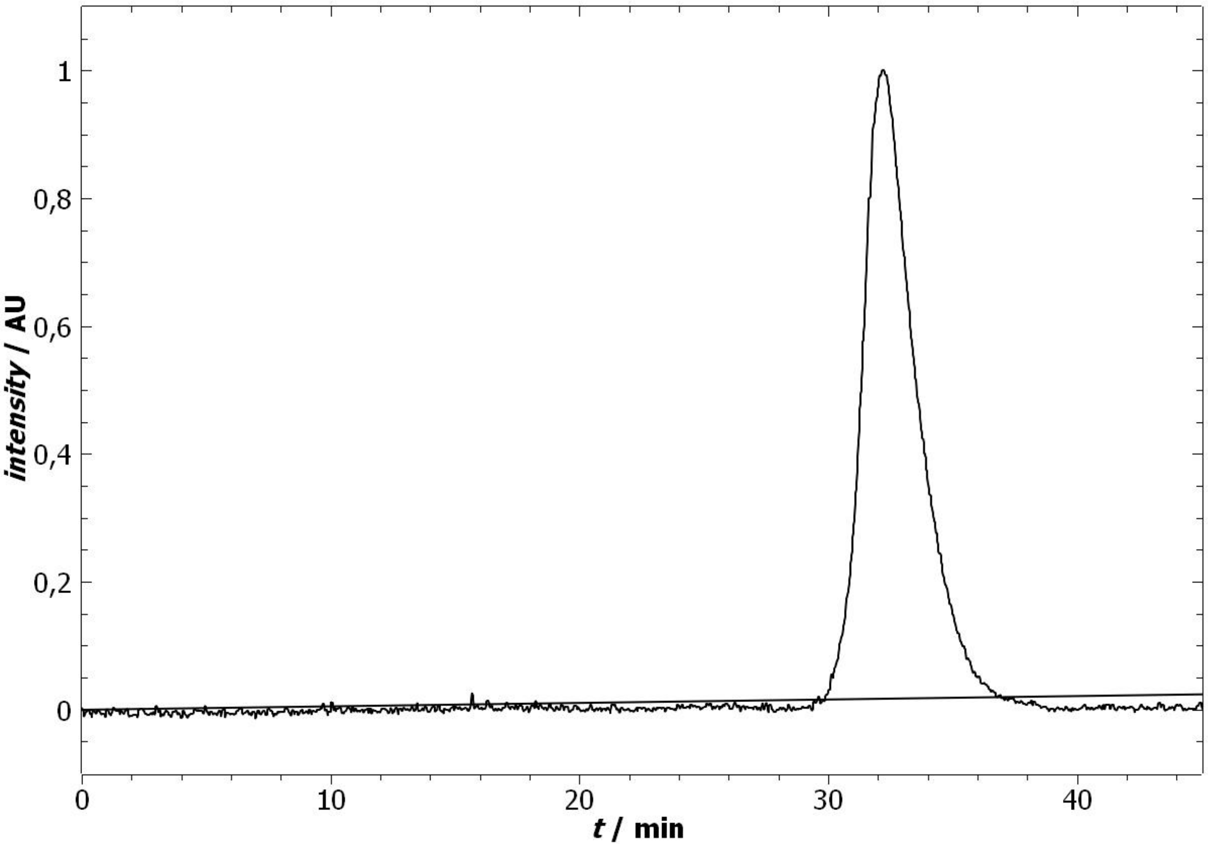

Figure S20. GPC-MALLS chromatogram of terpolymer precursor **16**, t_R = 32.46 min, detection by dRI.

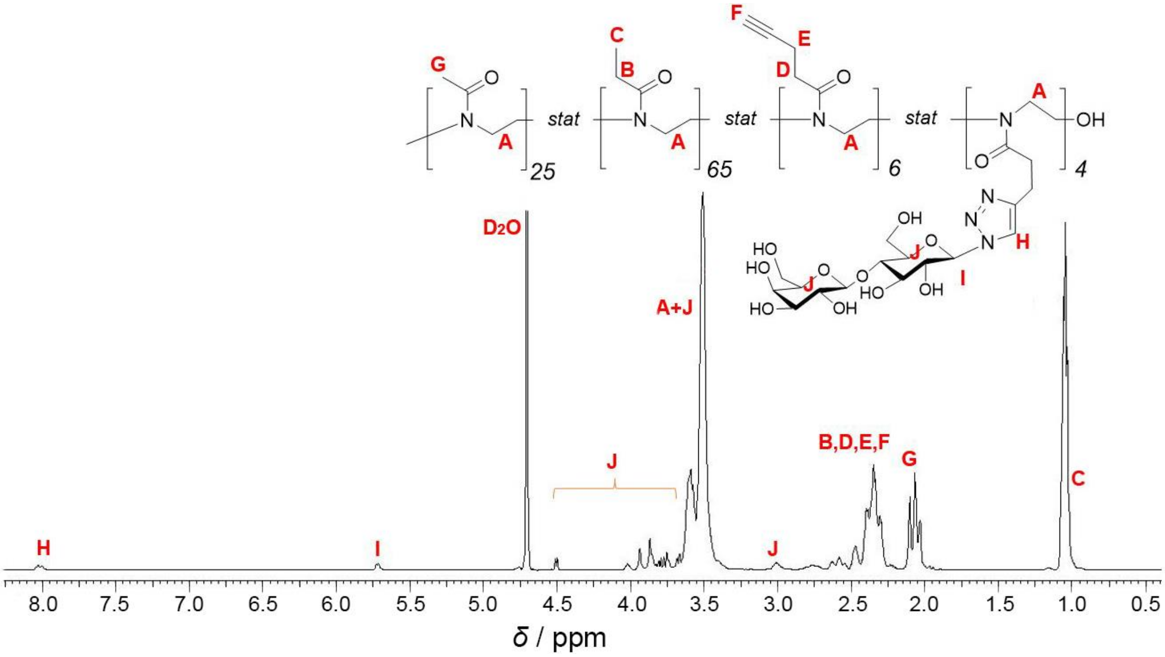

Figure S21. ^{1}H NMR spectrum of glyco-terpolymer **17** (Lac).

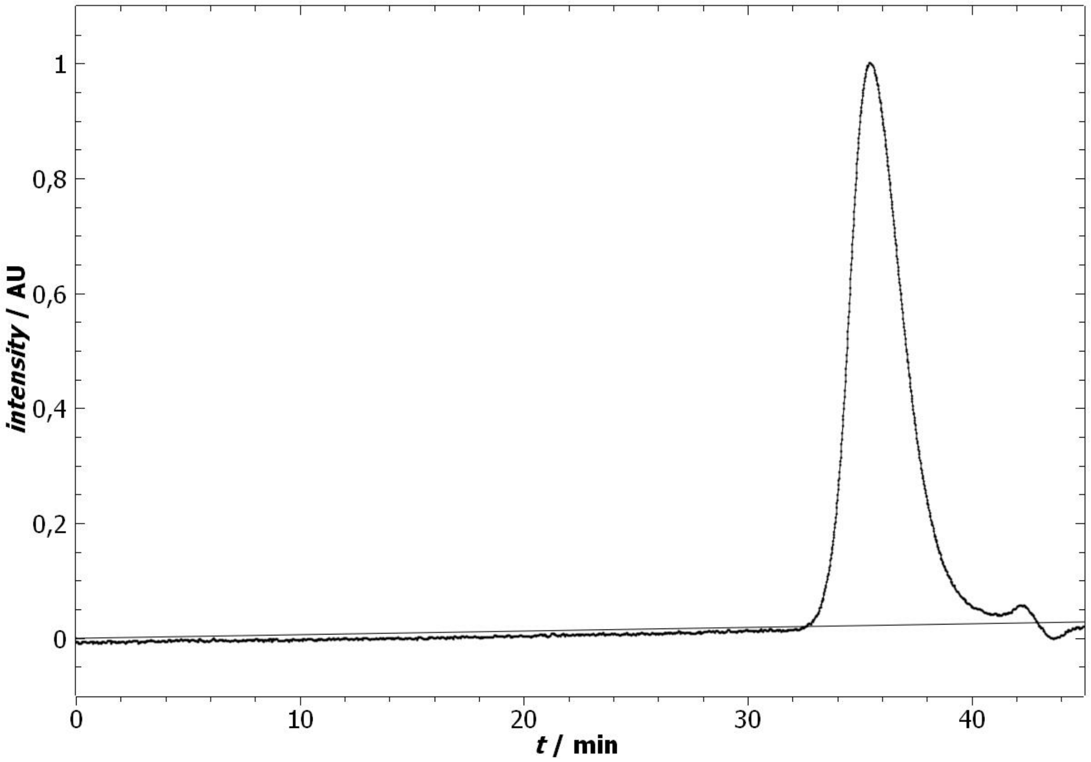

Figure S22. GPC-MALLS chromatogram of glyco-terpolymer **17** (Lac), t_R = 35.44 min, detection by LS.

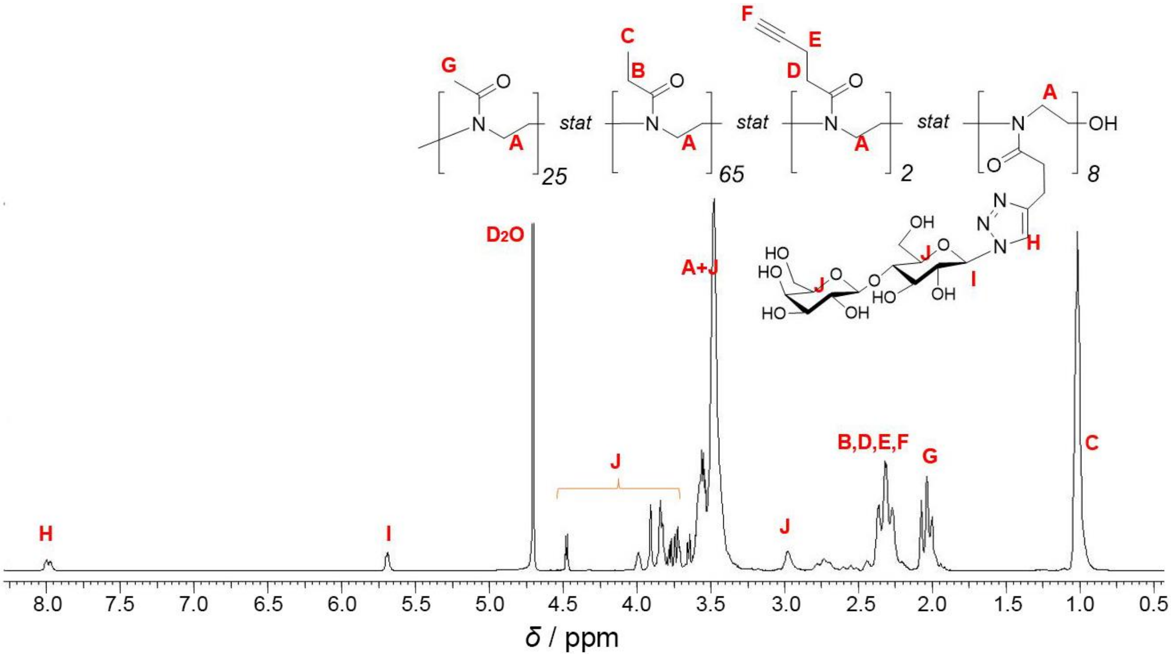

Figure S23. ^{1}H NMR spectra of glyco-terpolymer **18** (Lac-high).

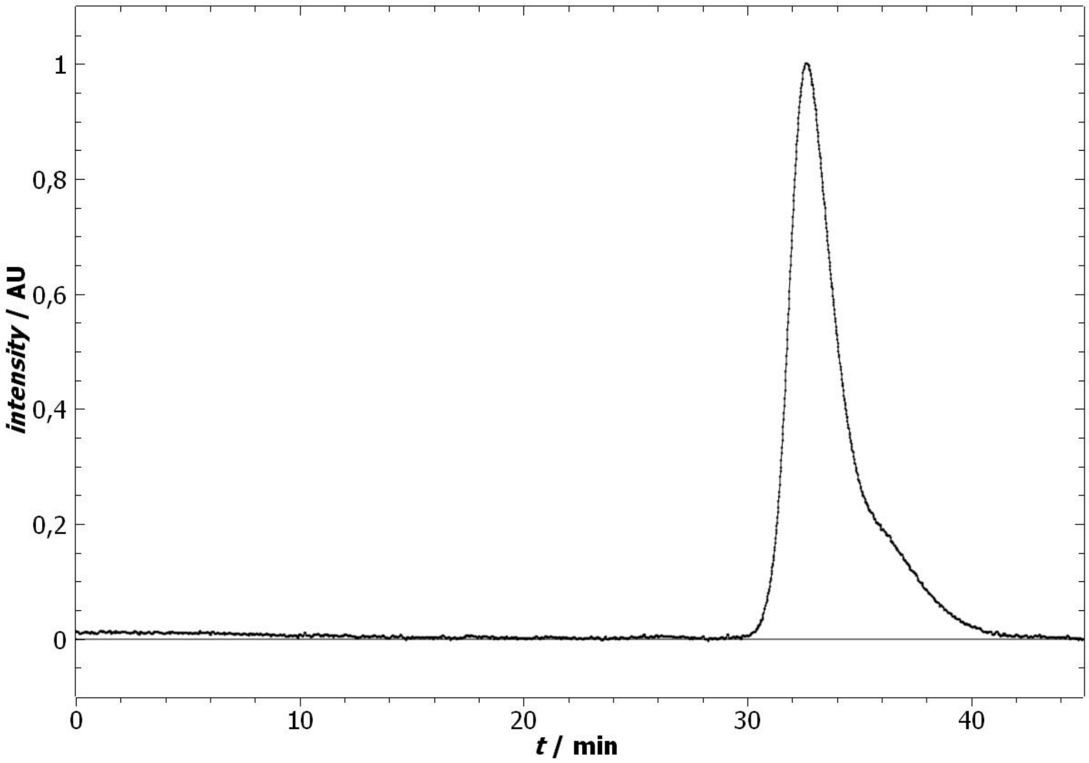

Figure S24. GPC-MALLS chromatogram of glyco-terpolymer **18** (Lac-high), t_R = 32.69 min, detection by LS.

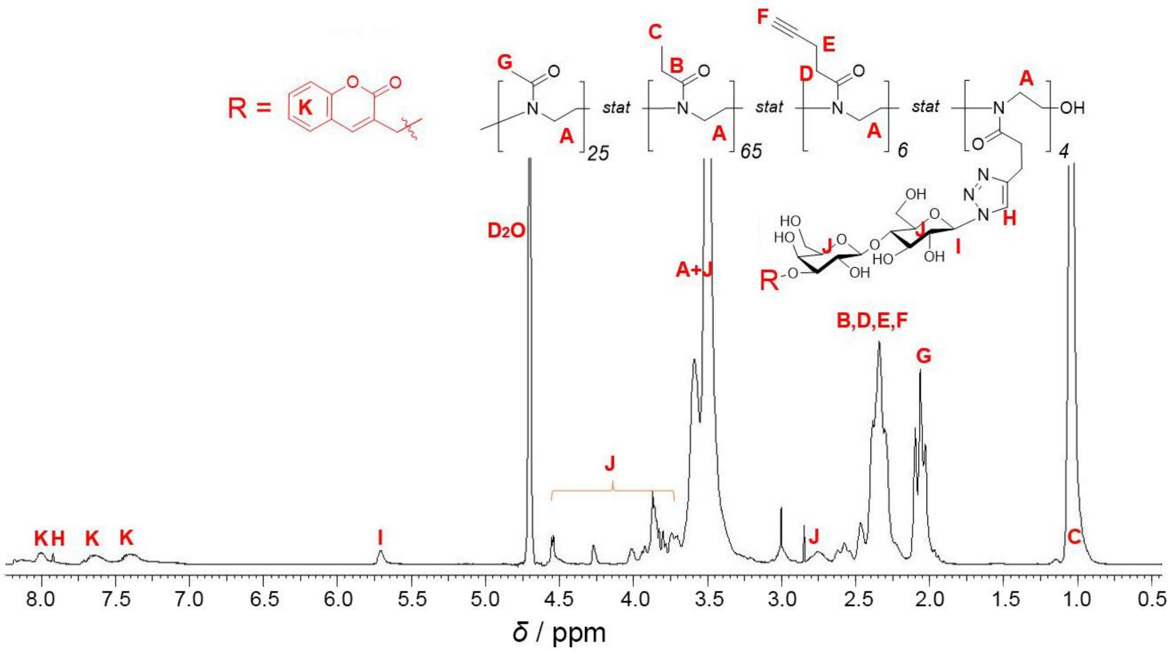

Figure S25. ^{1}H NMR spectrum of glyco-terpolymer **19** (Cou).

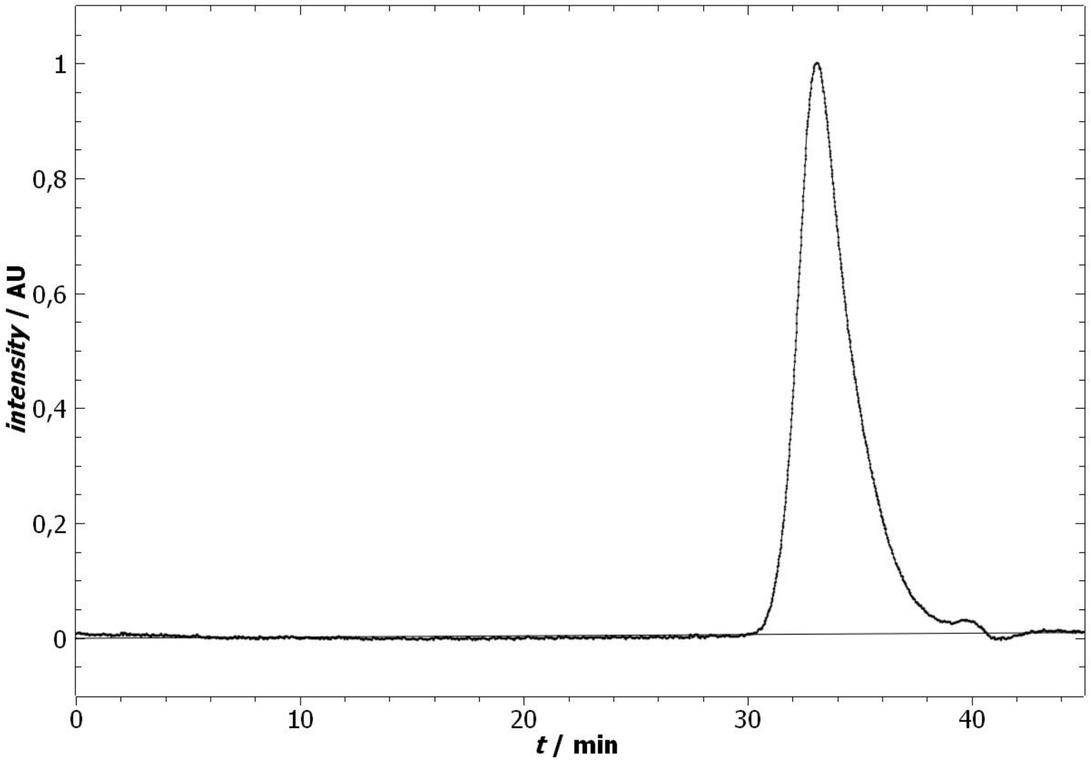

Figure S26. GPC-MALLS chromatogram of glyco-terpolymer **19** (Cou), t_R = 33.08 min, detection by LS.

4. Behavior of glycopolymers in solution

4.1 SEC-MALLS analysis of fluorescent glycopolymers

Fluorescently labeled copolymer precursor **13**, and glycopolymers **14** (Lac) and **15** (Cou) exhibited overestimated M_w (main text, Table 1). First, we ruled out the aggregation of copolymer **13** by comparing its SEC-MALLS characteristics to those of the non-fluorescent counterpart **10**. The retention times (t_R) of **10** and **13** were almost identical (Figure S27), giving almost the same molecular weights as calculated from polystyrene calibration (Table S2).

Table S2. Retention times and polystyrene (PS) molecular weight equivalents for **10** and **13**.

Sample	t_R [min]	M_w [g/mol]	M_w/M_n	dn/dc [mL/g]	$Kc/R_{\theta(q,c)}$ [a] [mol/g]	M_w [b] [g/mol]
**PS standard**	32.5	51 000	1.06	0.1600	n.a.	-
**10**	32.2	53 100	1.23	0.0674	1.00×10^{-4}	10 000
**13**	32.4	52 200	1.10	0.0755	6.25×10^{-6}	160 000

[a] Parameter shown on the Y-axis of the Zimm plot in the MALLS analysis.
[b] Molecular weight obtained as the reciprocal value of Zimm plot Y-axis intercept at $\theta = 0$.

Therefore, in the mobile phase used (DMF/10 mM LiCl), copolymer **13** did not form aggregates that would cause discrepancies in molecular weights obtained from the static light scattering detector.

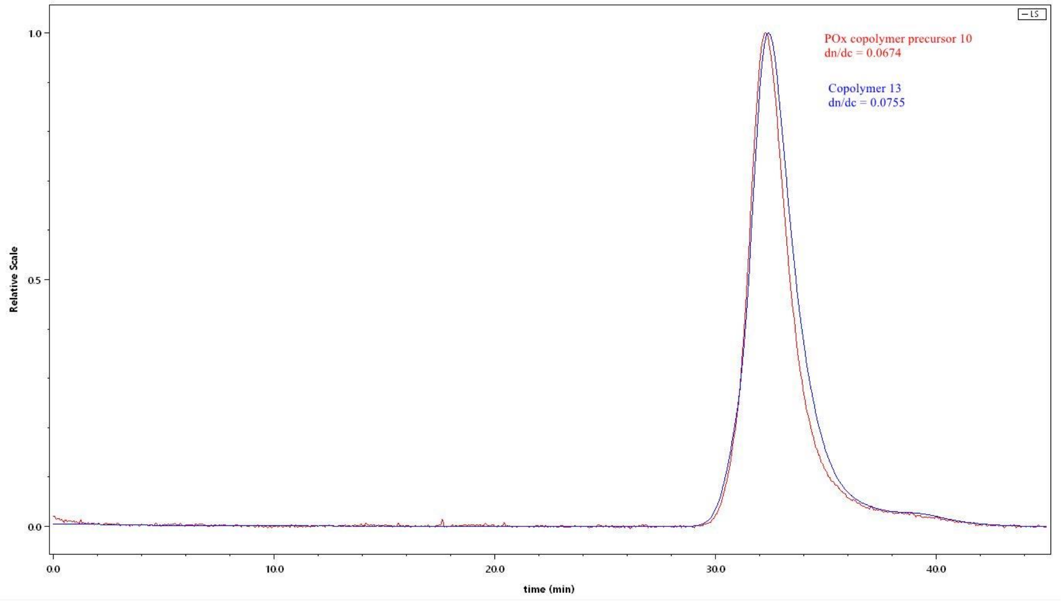

Figure S27. Overlay of SEC chromatograms of copolymers **10** and **13**.

Since the difference between the refractive index increments dn/dc determined by SEC-MALLS for each polymer was negligible (11%), it could not have been the reason for the overestimated M_w. However, we found that the ratio $Kc/R_{\theta(q,c)}$ was 16-fold lower for fluorescent copolymer **13** compared to control **10** in the same concentration. According to the Zimm equation, M_w is calculated as a reciprocal value of $Kc/R_{\theta(q,c)}$, yielding approx 16-fold higher M_w for the fluorescent polymers. This behavior is caused by the absorption of laser light (λ = 658 nm) within the boundaries of excitation and emission overlap of the fluorescent label

(Figure S28). The fluorescent molecule emits fluorescence at the same wavelength, apparently increasing the overall light scattering intensity of the sample, which leads to overestimated M_w.[3] Thus, the MALLS laser wavelength of λ = 658 nm was slightly absorbed by the Cy3-labeled samples (**13**, **14**, **15**), resulting in the emission of fluorescence at 658 nm (Figure S28). Moreover, the assessed spectra refer to the free Cy3 azide label, not accounting for the triazole formation and incorporation into the polymer lattice, which could have caused a red shift of the excitation/emission overlap of the labeled glycopolymers. Further investigation of this phenomenon is beyond the scope of this article.

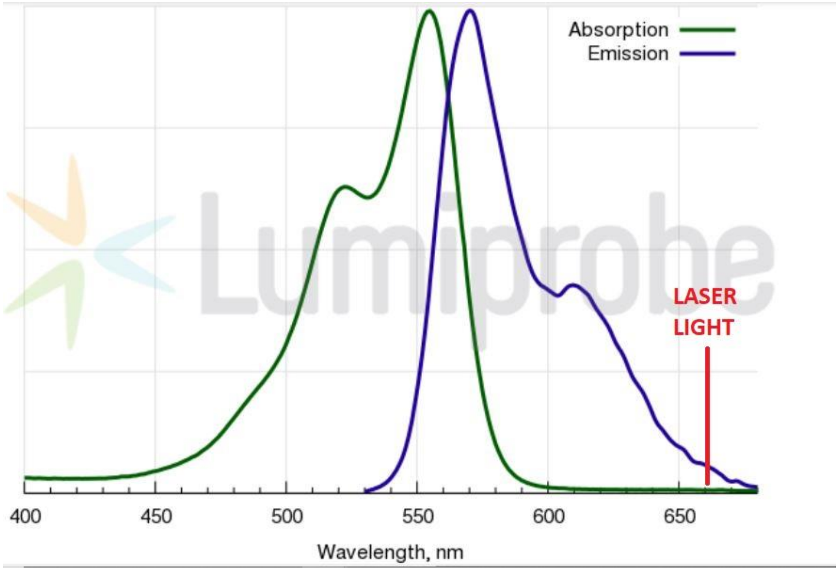

Figure S28. Overlay of emission and excitation spectra of Cy3 and laser wavelength of SEC-MALLS setup.

4.2. DLS characterization of coumaryl-loaded glycopolymers

To shed more light on the surprisingly poor *in vitro* results of glycopolymer **19** (Cou) – Figure 2 in the main text, we studied the behavior of coumaryl-loaded glycopolymers **12**, and **19** with low LCST (8-17 °C) in an aqueous solution. In the coumaryl-loaded glycopolymers, the secluded coumarylmethyllactosyl hydrophobic domains are surrounded by hydrophilic EtOx (in **12**) or EtOx/MeOx (in **19**) domains. Once the temperature rises above LCST, the hydrophobic domains expel water molecules and aggregate intra- and intermolecularly to minimize thermodynamically unfavorable interactions with solvent water. This hydrophobic interaction may produce large unstable aggregates that ultimately precipitate from the solution. Therefore, we investigated the aggregation behavior of these glycopolymers by DLS in a timeframe of 24 h.

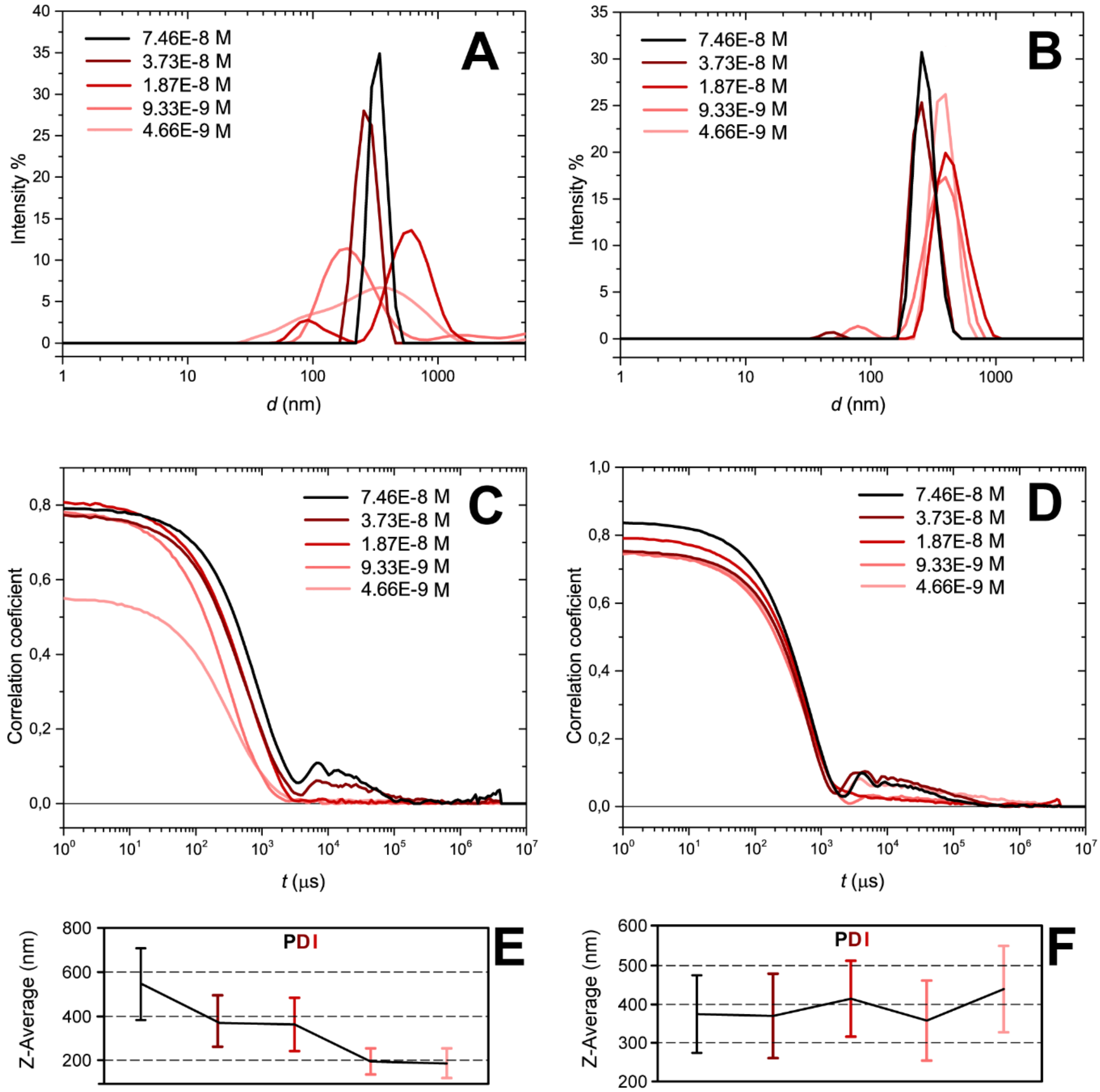

Figure S29. Analysis of glyco-copolymer **12** in an aqueous solution by DLS. Intensity-weighted distribution at t = 0 (**A**), and t = 24 h (**B**); autocorrelogram at t = 0 (**C**), and t = 24 h (**D**); z-average values for each concentration of **12** with PDI displayed as error bars at t = 0 (**E**) and t = 24 h (**F**).

From the intensity-weighted distribution, we can see that the DLS profile of glyco-copolymer **12** (Cou) at t = 0 h decreased in size with decreasing concentrations (Figure S29A), showing significant noise in slow diffusive modes of the autocorrelation function for higher concentrations (7.46×10^{-8} M and 3.73×10^{-8} M), and indicating very large sedimenting aggregates (Figure S29C). The distribution was bimodal, which also indicates the presence of smaller particles, possibly single chains. The intensity-weighted distribution after 24 h (Figure S29B) shows that large aggregates of several hundred nm were formed in all concentrations while the aggregation still proceeded (slow diffusion modes in autocorrelation function, Figure S29D). The z-average and polydispersity of 7.46×10^{-8} M **12** dropped after 24 h, indicating polymer sedimentation. However, we also observed that the z-average and PDI was quite the same for the whole concentration range after 24 h, indicating the presence of metastable particles of several hundreds of nm. For most diluted solutions, the z-average and PDI increased

after 24h, suggesting aggregation of the single chains. Therefore, at copolymer concentrations higher than 1.87×10^{-8} M, fast aggregation and sedimentation occurred, decreasing the effective concentration for galectin inhibition in cell assays while at lower concentrations, the sedimentation proceeded significantly slower.

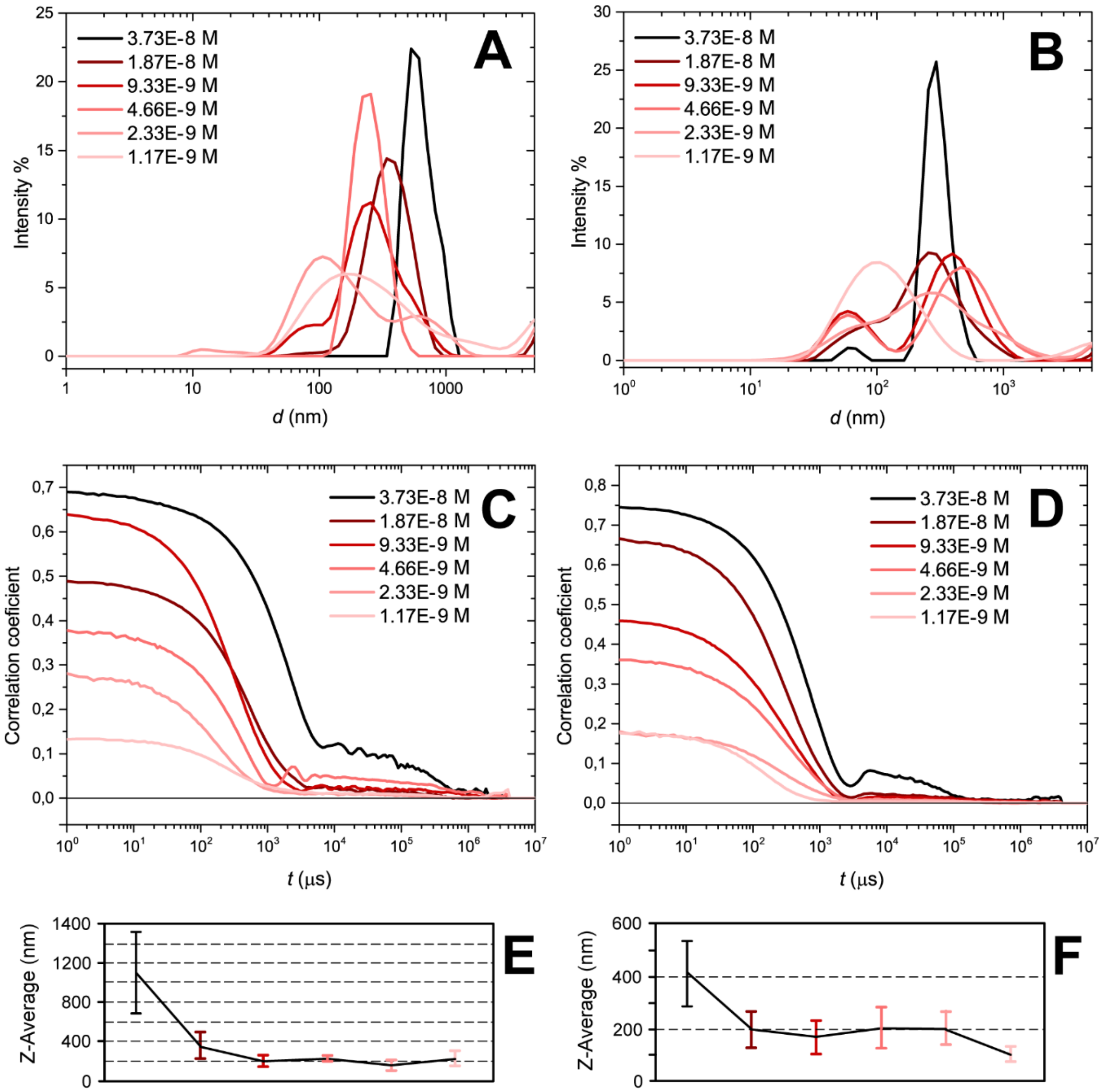

Figure S30. Analysis of glyco-copolymer **19** in an aqueous solution by DLS. Intensity weighted distribution at t = 0 (**A**) and t = 24 h (**B**); autocorrelogram at t = 0 (**C**), and t = 24 h (**D**); z-average values for each concentration of **19** with PDI displayed as error bars at t = 0 (**E**) and t = 24 h (**F**).

The intensity-weighted distribution profile for terpolymer **19** (Cou) at t = 0 h seems to decrease towards smaller sizes proportionally to the concentration (Figure S31A). Similar to **12** (Cou), we also observed high-order aggregates in slow diffusive modes in autocorrelation function for higher concentrations (Figure S30C). For concentrations under 9.33×10^{-9} M, the intensity peaks were bimodal, indicating smaller aggregates. In contrast to **12** (Cou), the intensity-weighted distribution did not increase in size after 24 h for lower concentrations (9.33×10^{-9} to 1.17×10^{-9} M) as indicated by a similar z-average and PDI in the 24 h timeframe (Figures S30E, F). Not only did these solutions maintain their parameters, but the autocorrelation intercept was also

concentration-dependent. This suggests that there may be only one population of scattering particles. The autocorrelation plot at t = 24 h (Figure S30D) also shows that slow diffusive modes are less noisy compared to **12** (Cou). At concentrations as high as 1.87×10^{-8} M, the z-average and PDI dropped even more significantly than in **12**, which indicates an even stronger sedimentation. Thus, if we solubilize **19** (Cou) at a sufficiently low concentration, single chains form stable particles, which did not precipitate in 24 h. We hypothesize that the different solution behavior of **12** and **19** is given by their different morphology; copolymer **12** is a purely statistical mixture of EtOx and BuOx in given ratios but terpolymer **19** may be of a more gradient nature since the rate of polymerization of MeOx is slightly higher compared to EtOx and BuOx. Therefore, the behavior of terpolymer **19** (Cou) in an aqueous solution has features of an amphiphilic block copolymer, forming micelle-like particles with a core of segregated coumarylmethyllactosyl hydrophobic chain ends stabilized by the MeOx-rich chain ends. Since the coumarylmethyllactosyl ligand is mostly confined within the micellar core, its efficiency in scavenging Gal-3 is deteriorated, which may reflect on the *in vitro* cellular experiments. The DLS measurement of model polymer **12** (Cou) in DMEM medium showed that the overall light scattering intensity was twice as high compared to aqueous solutions or in HBSS solution of a low ionic strength (Table S3). Except for water, the DLS data exhibited very high polydispersity, reaching PDI = 1; therefore, we only compared the overall intensities since aggregating solutions with larger particles generally sediment faster.

Table S3. Total scattering intensity and z-average of glyco-copolymer **12** (Cou) at 0 h and 24 h in different aqueous solutions.

Solvent	Time [h]	Derived count rate [cps]	z-Average [nm]
H_2O	0	5565	542
	24	6498	373
HBSS medium	0	4938	1192
	24	2769	1377
DMEM medium	0	13 069	25 000
	24	1219	2589

In the case of fluorescently labeled glyco-copolymer **15** (Cou), the time-dependent correlation of scattered light was polluted by non-coherent fluorescence, which resulted in a non-negligible noise factor and in a significantly decreased signal-to-noise ratio in the autocorrelation function. For fluorescent **15** at concentrations of up to 4 mg/mL, the signal-to-noise ratio was below 0.1, in contrast to non-fluorescent **12** with a signal-to-noise ratio of up to 0.8, even for much more diluted solutions. The autocorrelation functions for both polymer solutions are shown in Figure S31.

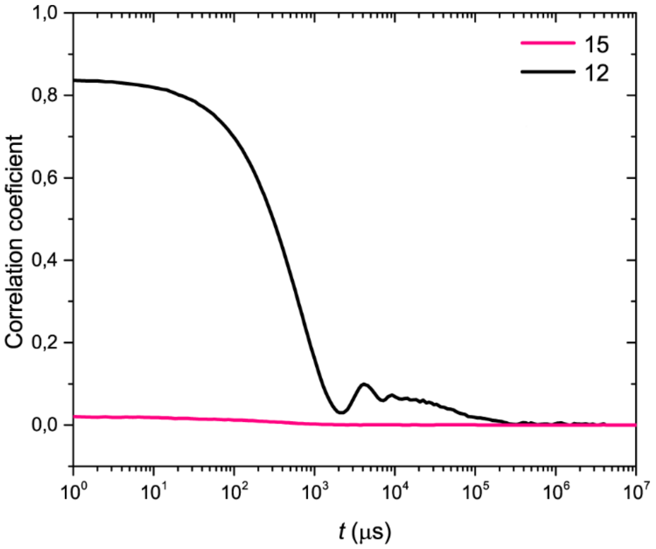

Figure S31. Overlay of autocorrelograms for fluorescent glyco-copolymer **15** and its non-fluorescent analog **12**.

There are hints in the literature that when determining the particle size of fluorescent materials using DLS, even the absorption of laser light does not necessarily rule out a correct measurement,[4] but it definitely contributes to a poorer signal-to-noise ratio due to the non-coherent fluorescence emission.

5. Affinity and selectivity of glycopolymers to Gal-3

5.1. Production and purification of recombinant Gal-3

The N-terminal His-tagged Gal-3 construct carrying an AVI-tag (Gal-3-AVI) cloned into the vector pET-Duet1 (restriction sites *Nco*I/*Asc*I) was expressed in *E. coli* BL21(λDE3) (Takara Bio, Kusacu, Japan) competent cells as described previously.[5] The *E. coli* strain contained an IPTG-inducible plasmid carrying the *birA* gene of biotin ligase for selective *in vivo* mono-biotinylation of the AVI-tag of the Gal-3 construct. The cells were grown in MDO medium (20 g/L yeast extract, 20 g/L glycerol, 1 g/L KH_2PO_4, 3 g/L K_2HPO_4, 2 g/L NH_4Cl, 0.5 g/L Na_2SO_4, 0.01 g/L thiamine hydrochloride) supplemented with ampicillin (150 µg/mL), and chloramphenicol (10 µg/mL) and complemented with 50 µM D-biotin (12 µg/mL) before induction. After induction with 1 mM IPTG at an OD_{600} of 0.6, the cultures were grown for additional 4 h at 37 °C and harvested by centrifugation (8880 × g, 20 min, 4 °C).

The cells were suspended in equilibration buffer (20 mM phosphate/500 mM NaCl/20 mM imidazole, pH 7.4) with 1% phenylmethylsulfonyl fluoride protease inhibitor. The cells were sonicated by UltraSonic Processor UP50 H (Ultrasound Technologies, Caldicot, UK) and centrifuged (20 230 × g, 20 min, 4 °C). Galectins were purified by affinity chromatography on Ni-NTA column (GE Medical Systems, Prague, Czech Republic). The washing step including 0.5% Triton X100 was incorporated to remove traces of lipopolysaccharide. Positive fractions were pooled and were dialyzed against phosphate-buffered saline (PBS), pH 7.5. The protein purity was confirmed by SDS-PAGE (12% gel) and its concentration was determined by Bradford assay calibrated for bovine serum albumin (BSA).

5.2. Biolayer interferometry (BLI)

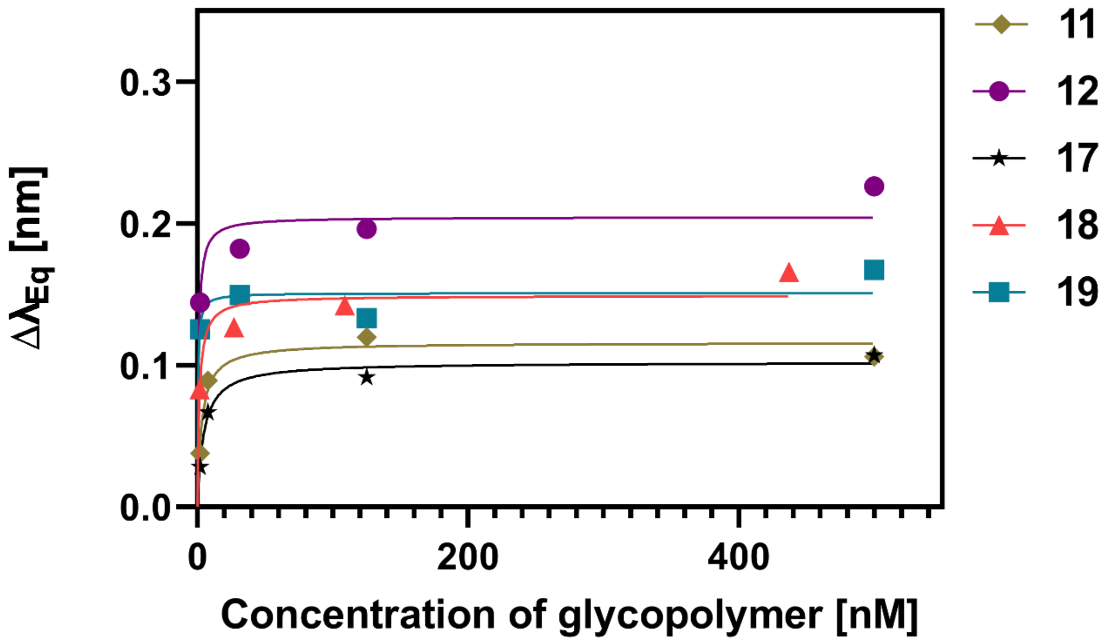

Figure S32. Steady-state analysis of the interaction between Gal-3-AVI and glycopolymers **11** (Lac); **12** (Cou); **17** (Lac); **18** (Lac-high), and **19** (Cou) determined by BLI.

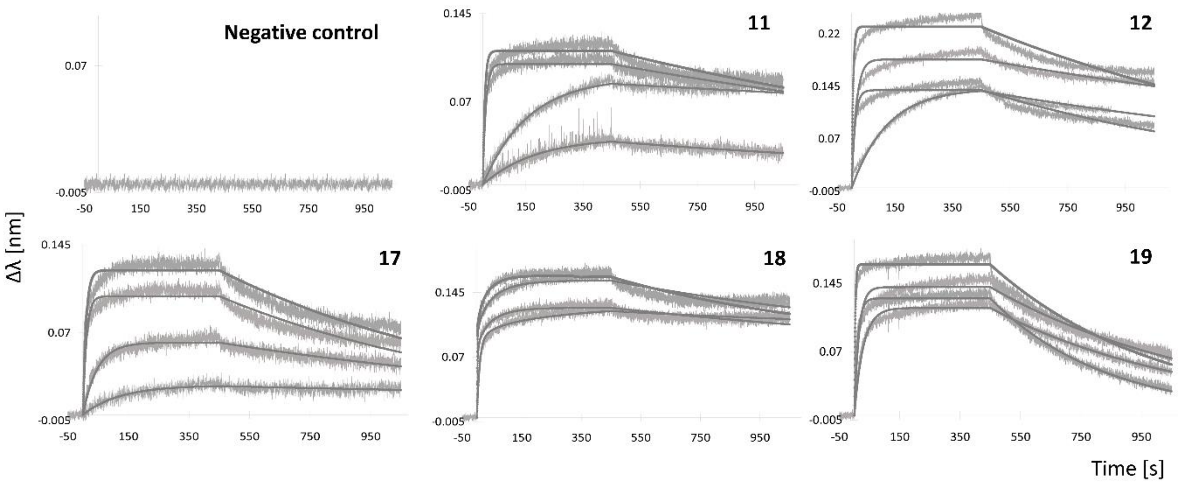

Figure S33. Kinetic analysis of the interaction between Gal-3-AVI and glycopolymers **11** (Lac); **12** (Cou); **17** (Lac); **18** (Lac-high), and **19** (Cou) determined by BLI fitted to one-to-one Langmuir kinetic model.

6. Biological Results

6.1. Metabolic activity assay

The cells in 96-well glass-bottom plates (Cellvis, P96-1.5H-N) were washed with PBS and incubated with 100 μL of fresh cultivation medium (DMEM with 2% FBS; without phenol red) containing resazurin (Sigma-Aldrich, R7017) in a final concentration of 40 μM. The cells were incubated for 4 h at 37 °C. Then, the fluorescence signal (Ex/Em = 530/590 nm) was detected on a Synergy™ HT Multi-Mode Microplate reader.

6.2. Immunofluorescence staining and visualization

The cells in glass 96-well cultivation plates (Cellvis, P96-1.5H-N) were fixed with 4% paraformaldehyde for 10 min at 25 °C. The cells were permeabilized with 0.1% Triton X-100 and 1% albumin in PBS for 20 min and then with 1% Tween-20 (for further 20 min). The cells were stained with the following primary antibodies: mouse monoclonal antibody against α-smooth muscle actin (αSMA, 1:400, Sigma-Aldrich, A2547) and rabbit recombinant monoclonal antibody against calponin 1 (1:400, Abcam, ab46794) for 3 h at 25 °C. The cells were then washed with PBS and incubated with F(ab')2-goat anti-mouse IgG (H+L) cross-adsorbed secondary antibody conjugated with Alexa Fluor™ 488 (Thermo Fisher Scientific, A11017) and goat anti-rabbit IgG (H+L) cross-adsorbed secondary antibody conjugated with Alexa Fluor™ 546 (Thermo Fisher Scientific, A11010), for 1 h at 25 °C (dilution of 1:400). The F-actin cytoskeleton was stained with Rhodamine Phalloidin (500 × diluted, Thermo Fisher Scientific, R415) and cell nuclei were counterstained with Hoechst 33258 (5 μg/mL, Sigma-Aldrich, B1155). The cells were visualized with an Olympus IX 71 epifluorescence microscope connected to a DP 70 digital camera (Olympus).

6.3. Isolation of RNA and qPCR

A Total RNA Purification Plus Micro Kit (Norgen Biotek, 48500) was used to isolate RNA from vascular smooth muscle cells and cardiac fibroblasts. Isolation of RNA from lung and right ventricular tissue was carried out with an Animal Tissue RNA Purification Kit (Norgen Biotek, 25700).

The purity and concentration of the RNA were measured with a NanoDrop One Spectrophotometer (Thermo Fischer Scientific, USA).

For reverse transcription of the isolated RNA, an Omniscript Reverse Transcription Kit (Qiagen, Cat. No. 205113) and a Random Primer Mix (New England Biolabs, cat. no. S1330S) were used according to the manufacturer’s protocol. The reaction mixture for reverse transcription contained 200 ng of the isolated RNA from cell cultures or 750 ng RNA from tissues in a reaction volume of 20 μL. The synthesized cDNA was preserved at -20 °C.

The mRNA expression in cells and tissues was determined with TaqMan Gene Expression Assays (Life Technologies) together with a 5×HOT FIREPol Probe qPCR Mix Plus (ROX) (Solis BioDyne, cat. no. 08-14-00001). FAM-labeled hydrolysis probes specific to ACTA2 (Rn01759928_g1), CNN1 (Rn00582058_m1), LGALS3 (Rn04219572_m1), COL1A1 (Rn01463848_m1), B2M (Rn00560865_m1) and ACTB (Rn00667869_m1) were used. ACTB was used as a reference gene in the cell cultures; B2M was used as a reference gene in the tissues. The polymerase chain reaction was executed in a 96-well plate (LightCycler 480 Multiwell Plate 96, cat. no.: 04729692001, Roche; final reaction volume of 10 μL per well) in a LightCycler® 480 System (Roche). The reaction profile was composed of pre-incubation for

2 min at 50 °C, denaturation/enzyme activation for 10 min at 95 °C followed by 45 cycles of denaturation (15 s at 95 °C) and annealing/elongation (1 min at 60 °C). The relative gene expression levels were calculated using a standard curve-based method.

6.4. Western blotting

The right ventricles or lungs were pulverized to a fine powder in liquid nitrogen and dissolved in eight volumes of ice-cold homogenization buffer - 12.5 mM Tris-HCl, 250 mM sucrose, 2.5 mM EGTA (egtazic acid), 1 mM EDTA (ethylenediaminetetraacetic acid), 6 mM ß-mercaptoethanol, protease inhibitor cocktail (Roche) and phosphatase inhibitor cocktail (Roche), pH 7.4 - followed by Potter-Elvehjem homogenization. The protein content was measured by the Bradford method (Bio-Rad). The samples (right ventricle and lung homogenates or isolated heart fibroblasts) were subjected to SDS electrophoresis on 10% bis-acrylamide gels at 20 mA/gel for 90 min on a Mini-Protean III apparatus (Bio-Rad). After electrophoresis, the resolved proteins were transferred to PVDF membranes (0.2 µm pore size, Bio-Rad). After blocking with 5% blotting-grade blocker (Bio-Rad) in Tris-buffered saline with Tween 20 (TTBS) for 60 min at room temperature, the membranes were probed with appropriate primary and secondary antibodies: anti-α-smooth muscle actin (Sigma-Aldrich, A2547, 1:1000, overnight, 4 °C), anti-calponin 1 (Invitrogen, MA5-11620, 1:1000, overnight, 4 °C), anti-Gal-3 (Abcam, ab2785, 1:1000, overnight, 4 °C), anti-vinculin (Sigma-Aldrich, SAB4200729, 1:5000, overnight, 4 °C), and anti-mouse secondary antibody (ThermoFisher, 31432, 1:10 000, 60 min, room temperature). The membranes were visualized by enhanced chemiluminescence (ECL) substrates (SuperSignal™ West Dura Extended Duration Substrate or SuperSignal™ West Femto Maximum Sensitivity Substrate, Thermo Scientific) using a ChemiDoc™system (Bio-Rad). The relative abundance of analyzed proteins was quantified by ImageQuant software. The amounts of protein applied to the gel varied for each protein to achieve linearity. Each sample was analyzed at least three times. The analysis was normalized to the total protein (fibroblasts and right ventricle homogenate) or µg of the tissue (lung homogenate). The results were recalculated to vinculin used as a housekeeping protein.

6.5. Hydroxyproline assay

Cells were lysed in RIPA buffer (R0278, Sigma-Aldrich), and Pierce BCA assay (23225, ThermoFisher Scientific) was performed according to the manufacturer's instructions to determine the total protein content in the sample. The samples were then proteolytically digested in 6N HCl at 120 °C for 3 h. The amount of collagen deposited in the extracellular matrix was determined as the hydroxyproline content in the sample. The samples were assayed using the Hydroxyproline Colorimetric Assay Kit (MAK008, Sigma-Aldrich), according to the manufacturer's instructions. Both assays were measured using a VersaMax Absorbance Microplate Reader (Molecular Devices, USA) at 560 nm. Data are presented as the relative concentration of hydroxyproline to the total protein content per sample.

6.6. Confocal microscopy

Live cells incubated with fluorescently labeled polyoxazoline polymers (final concentration of 10 µM in the cell culture medium) were observed using an Andor Dragonfly 503 scanning disc confocal microscope equipped with a Zyla 4.2 PLUS sCMOS camera (Andor Technology Ltd.) and objective HC PL APO 63x/ 1.20 W CORR CS2. The cytoplasmic membrane of live cells was stained with CellMask Deep Red Plasma Membrane Stain (1000 × diluted, Thermo Fisher Scientific, C10046) and nuclei with Hoechst 33342 dye (5 µg/mL, Sigma-Aldrich, B2261) for 5 min at 37 °C in PBS.

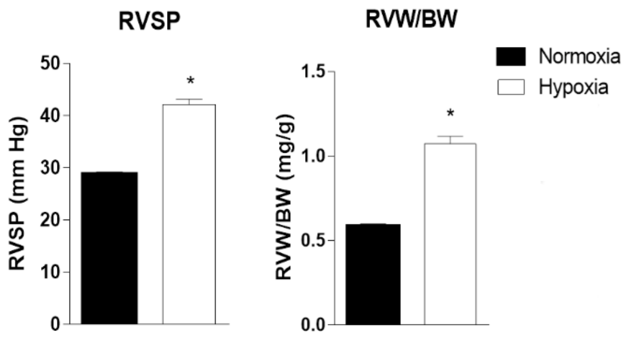

Figure S34. Rats exposed to hypoxia developed severe pulmonary hypertension. Right ventricular systolic pressure (RVSP) and relative right ventricular weight (RVW/BW) of rats exposed to intermittent hypobaric hypoxia simulating an altitude of 7000 m or control rats bred in normoxic conditions. Mean + SEM, $n = 8$. Student's t-test, $p \leq 0.05$. * significant difference compared to normoxia.

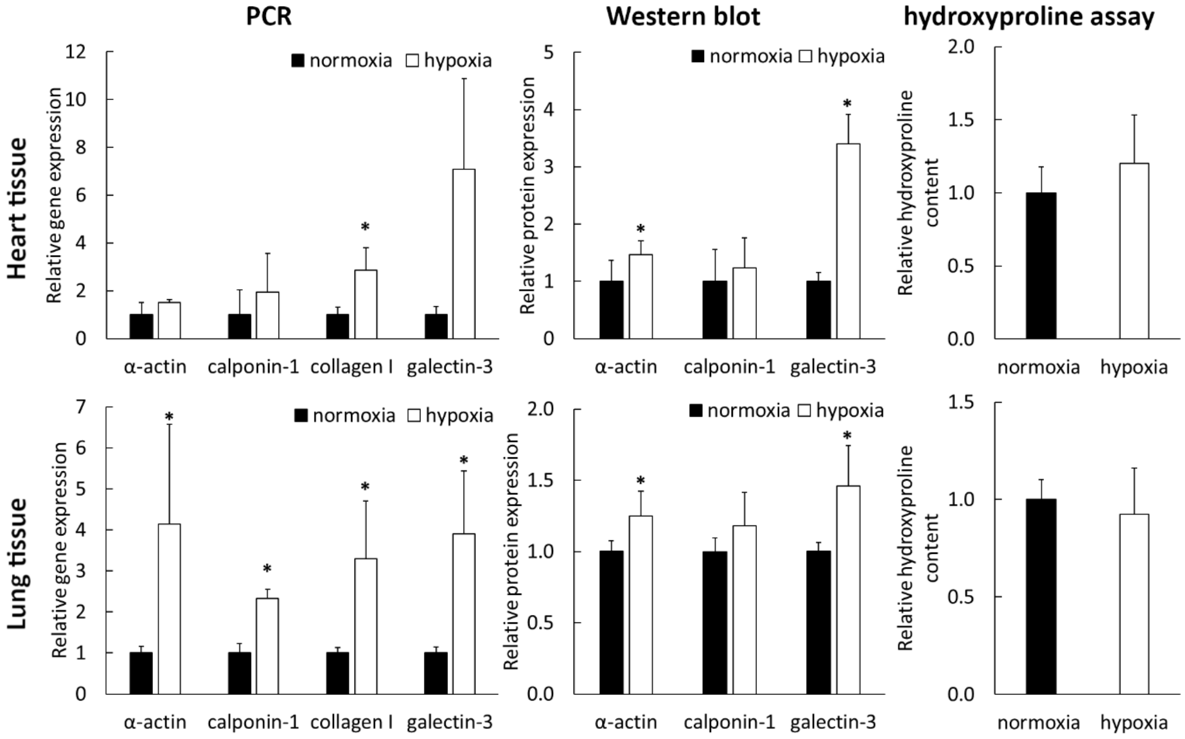

Figure S35. Analysis of gene and protein expression in the tissues of normoxic and hypoxic rats. In heart and lung tissue, the expression of α-actin, calponin-1, and Gal-3 was determined both at mRNA (PCR) and protein level (Western blot). The expression of collagen I was determined only by PCR. Total collagen content was estimated by measuring hydroxyproline concentration in tissue samples (hydroxyproline assay). Mean + SD, $n = 5$. Student's t-test, $p \leq 0.05$. * significant difference compared to normoxia.

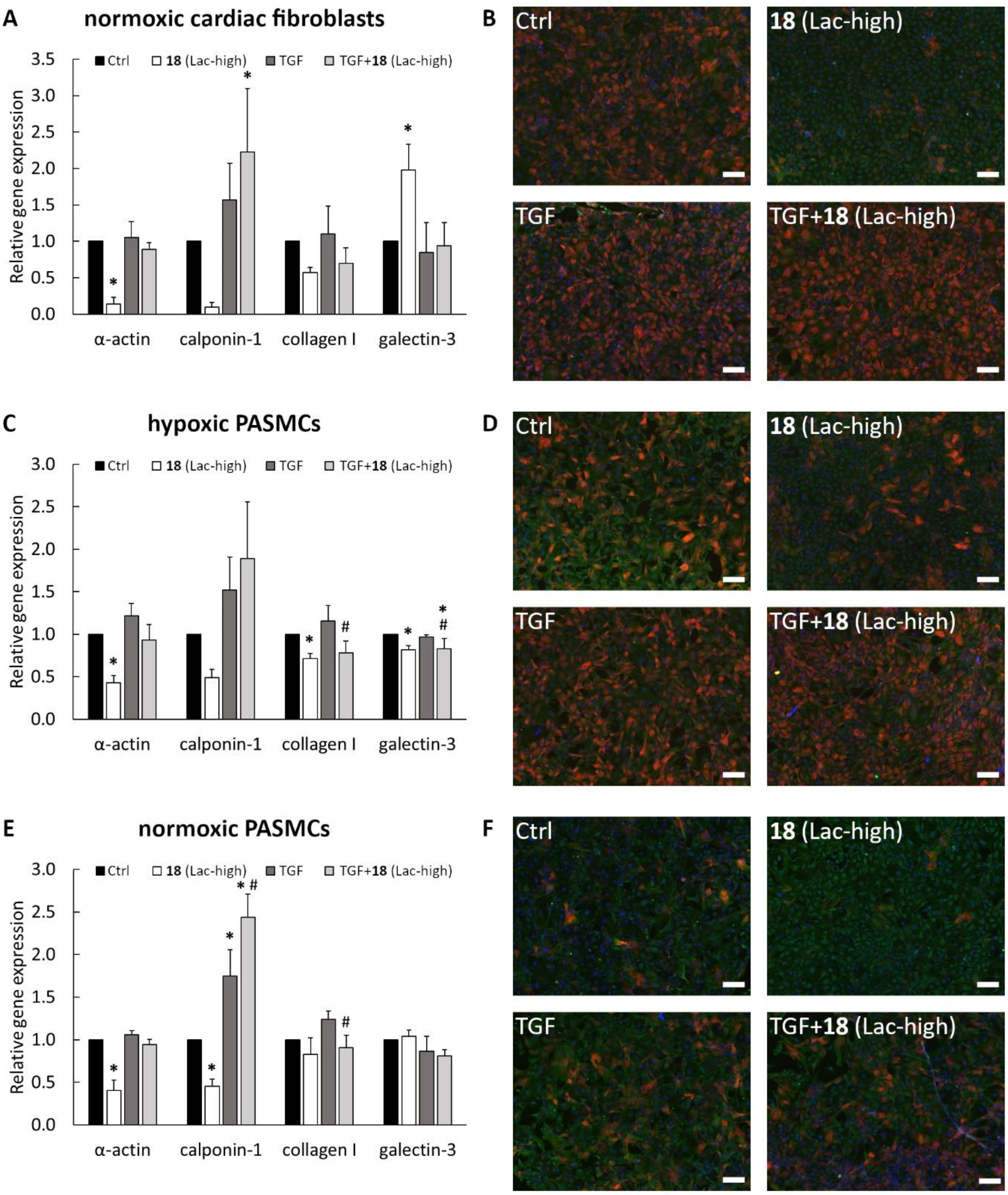

Figure S36. Glycopolymer **18** (Lac-high) affects gene and protein expression also in normoxic cardiac fibroblasts and in hypoxic and normoxic PASMCs. Glycopolymer **18** (Lac-high) was added to the cell culture medium of unaffected or TGFβ-stimulated cells. The qPCR analysis of gene expression of α-actin, calponin-1, collagen 1, and Gal-3 on day 1 after adding polymer in normoxic cardiac fibroblasts (**A**), hypoxic PASMCs (**C**) and normoxic PASMCs (**E**). Mean + SD from three independent experiments. One Way ANOVA, Student-Newman-Keuls test, ($p \leq 0.05$). * significant difference compared to Ctrl, # significant difference compared to TGF. Immunofluorescence staining of smooth muscle α-actin (**green**) and calponin-1 (**red**) on day 3 after adding polymer in normoxic cardiac fibroblasts (**B**), hypoxic PASMCs (**D**), and normoxic PASMCs (**F**); cell nuclei (**blue**). The scale bar represents 200 μm.

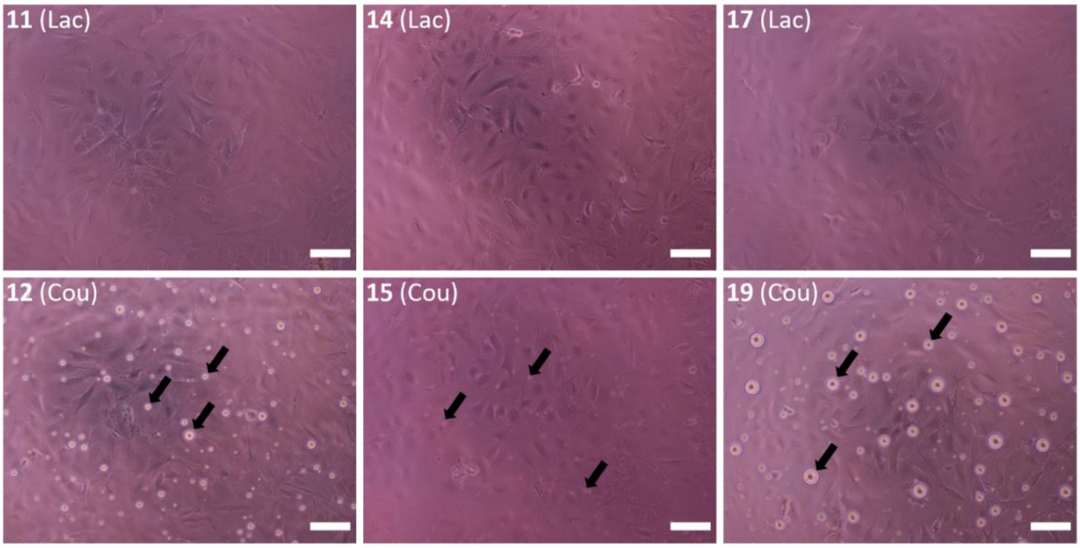

Figure S37. Brightfield images of cell cultures incubated with glycopolymers. Hypoxic PASMCs were incubated in a cell culture medium with polymers loaded with lactosyl ligands (**11, 14, 17**) or loaded with coumaryllactosyl ligands (**12, 15, 19**) for 24 h. **11, 12, 17**, and **19** were added in 100 µM concentration; **14** and **15** in 10 µM concentration. Coumaryllactosyl-loaded polymers **12**, **15**, and **19** formed micellar aggregates in cell culture conditions (indicated by arrows). The scale bar represents 100 µm.

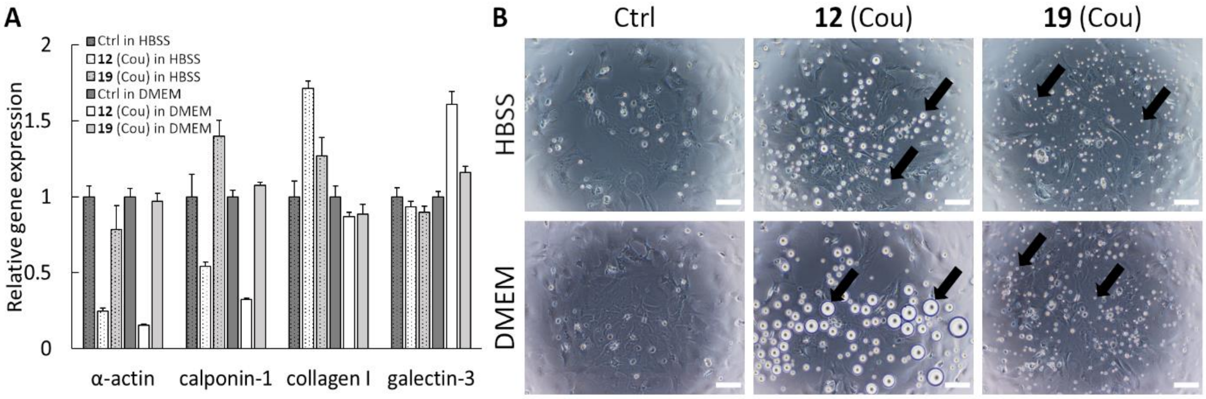

Figure S38. The influence of aggregation behavior of coumaryl-loaded polymers on their biological effect *in vitro*. Hypoxic cardiac fibroblasts were incubated in low-ionic-strength HBSS solution or high-ionic-strength DMEM medium with polymers **12** or **19** carrying coumaryllactosyl ligands for 1 day. The qPCR analysis of gene expression of α-actin, calponin-1, collagen I and Gal-3 (**A**). Brightfield images of cell cultures. Polymer aggregates are indicated by arrows. The scale bar represents 100 µm (**B**).

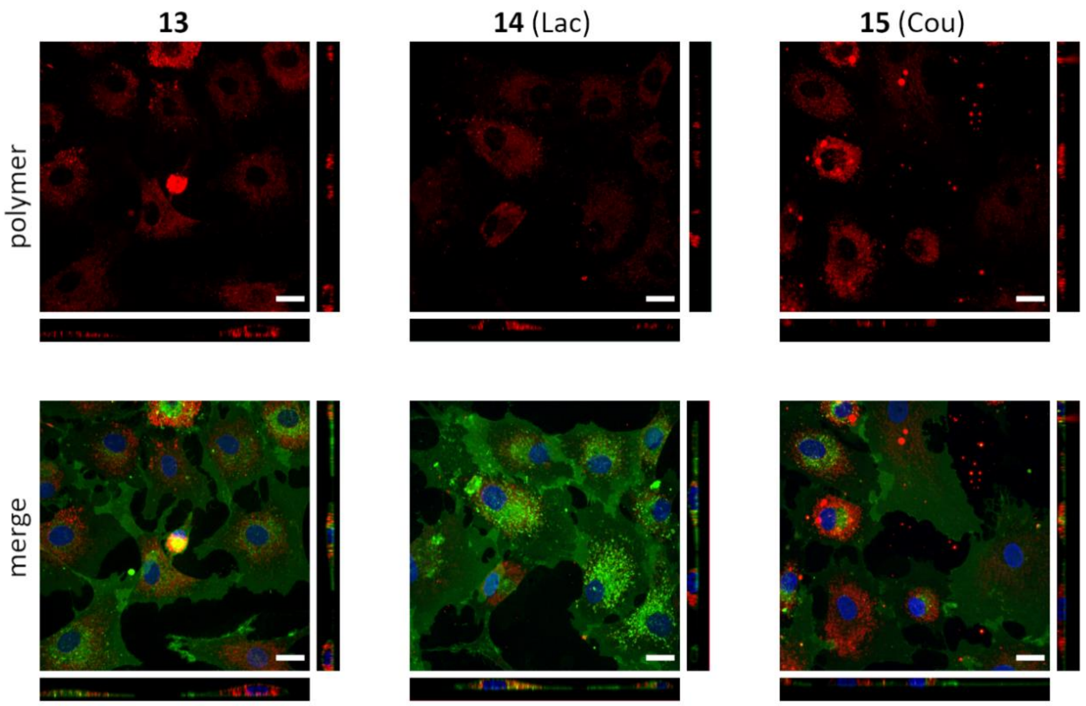

Figure S39. Effect of lactose on the cellular uptake of polyoxazoline polymers in hypoxic rat cardiac fibroblasts. Cells were incubated with the indicated **fluorescent polymer** (10 µM) and lactose (100 mM). After 24 h, confocal images were taken. Polymer - **red**. Merge - cell membranes were stained with CellMask Deep Red Cell Membrane Staining (**green**) and nuclei with Hoechst 33324 (**blue**). The scale bar represents 20 µm.

7. References

1. Vlachová, M.; Tran, V. N.; Červený, J.; Dolníček, F.; Petrásková, L.; Pelantová, H.; Kundrát, O.; Cvačka, J.; Bosáková, Z.; Křen, V.; Lhoták, P.; Viktorová, J.; Bojarová, P. Galectin-targeting glycocalix[4]arenes can enter the cells. *Chem. Commun.* **2023**, *59* (69), 10404-10407.
2. Konvalinková, D.; Dolníček, F.; Hovorková, M.; Červený, J.; Kundrát, O.; Pelantová, H.; Petrásková, L.; Cvačka, J.; Faizulina, M.; Varghese, B.; Kovaříček, P.; Křen, V.; Lhoták, P.; Bojarová, P. Glycocalix[4]arenes and their affinity to a library of galectins: the linker matters. *Org. Biomol. Chem.* **2023**, *21* (6), 1294-1302.
3. Waters | Wyatt Technology. "Characterizing Fluorescent Macromolecules and Nanoparticles". AZoNano. https://www.azonano.com/article.aspx?ArticleID=4812. (accessed May 13, 2024).
4. Geissler, D.; Gollwitzer, C.; Sikora, A.; Minelli, C.; Krumrey, M.; Resch-Genger, U. Effect of fluorescent staining on size measurements of polymeric nanoparticles using DLS and SAXS. *Anal. Methods-Uk* **2015**, *7* (23), 9785-9790.
5. Bumba, L.; Laaf, D.; Spiwok, V.; Elling, L.; Křen, V.; Bojarová, P. Poly-*N*-acetyllactosamine neo-glycoproteins as nanomolar ligands of human galectin-3: binding kinetics and modeling. *Int. J. Mol. Sci.* **2018**, *19* (2), 372.
